# Perspectives on the Role of Enzymatic Biocatalysis for the Degradation of Plastic PET

**DOI:** 10.3390/ijms222011257

**Published:** 2021-10-19

**Authors:** Rita P. Magalhães, Jorge M. Cunha, Sérgio F. Sousa

**Affiliations:** 1 UCIBIO—Applied Molecular Biosciences Unit, BioSIM—Departamento de Biomedicina, Faculdade de Medicina, Universidade do Porto, 4200-319 Porto, Portugal; ritaprata1@hotmail.com (R.P.M.); jorgemcunha97@gmail.com (J.M.C.); 2 Associate Laboratory i4HB—Institute for Health and Bioeconomy, Faculdade de Medicina, Universidade do Porto, 4200-319 Porto, Portugal

**Keywords:** PET, plastic, biodegradation, plastic degradation, PETase, MHETase, LCC, cutinase, *Ideonella sakaiensis*, biocatalysis

## Abstract

Plastics are highly durable and widely used materials. Current methodologies of plastic degradation, elimination, and recycling are flawed. In recent years, biodegradation (the usage of microorganisms for material recycling) has grown as a valid alternative to previously used methods. The evolution of bioengineering techniques and the discovery of novel microorganisms and enzymes with degradation ability have been key. One of the most produced plastics is PET, a long chain polymer of terephthalic acid (TPA) and ethylene glycol (EG) repeating monomers. Many enzymes with PET degradation activity have been discovered, characterized, and engineered in the last few years. However, classification and integrated knowledge of these enzymes are not trivial. Therefore, in this work we present a summary of currently known PET degrading enzymes, focusing on their structural and activity characteristics, and summarizing engineering efforts to improve activity. Although several high potential enzymes have been discovered, further efforts to improve activity and thermal stability are necessary.

## 1. Introduction

Synthetic plastic materials are long-chain polymers with high durability and resistance, typically derived from fossil-fuel by-products [1]. Massive plastic production and usage began in 1950 [2,3], when 2 million tons were produced annually [4]. Production has tripled in the last 20 years [5]. According to a recent Plastics Europe [6] report, 359 million tons of plastic were produced worldwide in 2018. In 2019, this number grew to 368 million tons [6], and is expected to surpass 34 billion tons by 2050 [2], cumulative, of which 12 billion are expected to be deposited in landfills or contaminated natural environments [7]. The industries of packaging and construction amount to over 60% of plastic demand [6]. Most plastics produced are single-use, poorly discarded products, resulting in accumulation in various ecosystems [8] with serious consequences for soil, marine, and terrestrial species [9,10,11,12].

The most common strategy for plastic elimination is landfill deposition [3,13]. This is a hazardous strategy with countless disadvantages [14]. Degradation of plastic waste is slow, and its accumulation leads to environmental contamination of groundwater, marine, and terrestrial environments. Incineration is another harmful and widely used strategy, as it leads to the spreading of toxic and volatile wastes, which are dangerous to the environment and human beings [14,15].

Industrial plastic recycling is the conversion of plastic waste into reusable materials, typically by mechanical or chemical processing. However, currently employed tactics are not sufficient to satisfy the recycling needs of the ever-growing plastic industry [3]. Mechanical recycling does not change the chemical structure of the polymer, and implicates previously collecting, sorting, shredding, grinding, melting, and washing of the material [16,17,18]. These methods have several disadvantages—they are not applicable to temperature-sensitive or multilayer plastics [19], often result in material deterioration, and sorting brings in economical and applicability issues [20]. Furthermore, mechanical recycling is hazardous for the environment and constitutes a public health danger, since it is a common source of toxic organic compounds [21]. Chemical recycling typically leads to the polymer’s degradation into its monomeric building blocks [18], via hydrolysis, methanolysis, and glycolysis strategies. However, it normally involves a high cost and high energy process, consuming active catalysts [22]. In addition, chemical methods are environmentally harmful, as they constitute a source of volatile organic compounds [23,24]. Biodegradation, the cleavage of plastic polymers into monomers by microorganism produced enzymes [25], is an environmentally friendly strategy [26] that has been gaining more attention. These microorganisms can use degradation products as a carbon or energy source through excretion of extracellular enzymes and metabolites [27,28,29]. These enzymes typically degrade the polymers with the assistance of water molecules and are known as hydrolases [30,31]. The by-products of enzymatic processing can be used for different applications, besides being naturally up taken by cells [13,24]. Spontaneous biodegradation is often preceded by oxidizing agents or UV-light [32]. Recent advances have placed plastic biodegradation at the top of promising recycling strategies, even if several limitations still remain to be solved [33].

**Figure 1:**
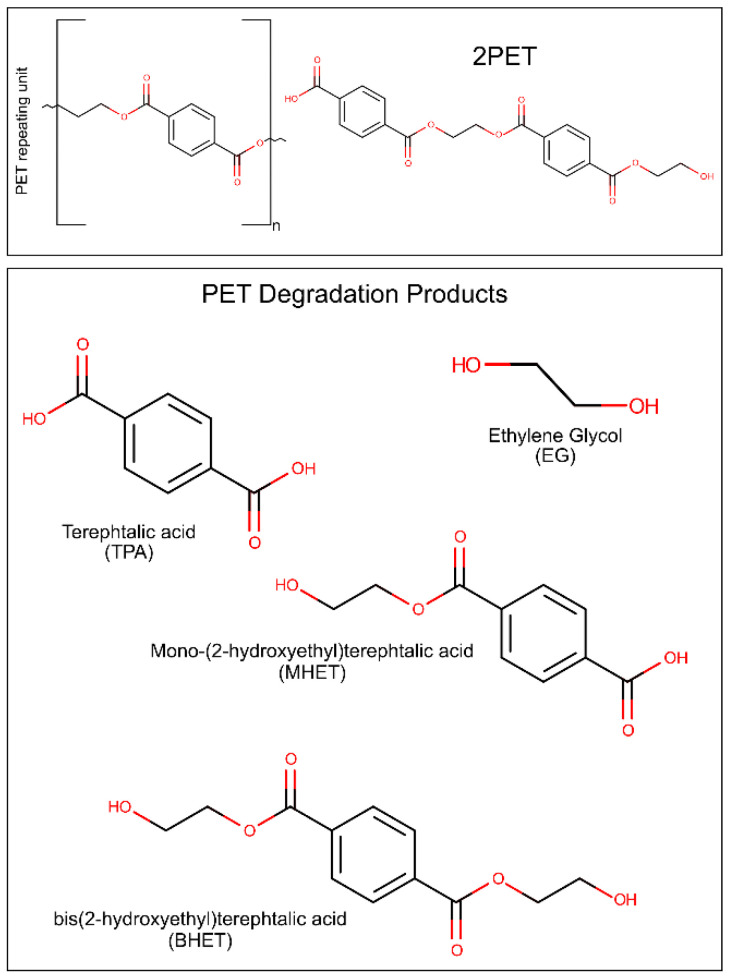
Scheme of the PET repeating unit, a molecule of 2PET, and the main PET degradation products.

Polyethylene terephthalate (PET) is one of the most used and synthesized plastics [9]. PET, derived from crude-oil [34], is composed of repeating units of terephthalic acid (TPA) and ethylene glycol (EG) monomers [35], and exists as an amorphous as well as a semi-crystalline synthetic polymer [36]. It is typically produced by condensation of TPA and EG or bis(2-hydroxyethyl)terephthalic acid (BHET) and EG [37]. The main components and degradation products of PET are represented in Figure 1. The physiochemical properties of PET result in diverse applications in the textile, packaging and bottle producing industries [9]. PET bottle production in 2016 was of 485 billion unities [6]. In 2021, the estimate of production increased to 583 billion bottles [35]. PET fibers are extremely attractive for their high durability, elasticity, strength, and resistance to chemicals [38]. Unfortunately, the same characteristics that make PET an attractive material also result in difficult degradation and consequent accumulation in the environment [39]. Several strategies have been described for PET mechanical [21,40] and chemical [23,24,41,42] recycling, but the high cost and environmental impact of these methods lead to an urgent need for the development of more effective and greener alternatives.

PET is a high molecular weight polyester [43,44], which presents a stable backbone and crystallinity, which inhibits natural breakdown [34,45]. As a semi-crystalline polymer with a glass transition temperature (T_g_) of 76 °C [37], PET presents amorphous and crystalline regions [46]. The T_g_ is the temperature at which a reversible transition between a more tightly packed crystalline state and a more rubber-like amorphous state happens [47]. When PET is mostly in an amorphous stage, the polymeric chain is more readily available for enzymatic mediated biodegradation [48]. This is due to the increased mobility of PET chains in an amorphous stage in comparison with the crystalline state [49].

PET is by far the most studied polymer in terms of biodegradation, and the one with the largest number of degrading enzymes identified and characterized [50]. The first enzyme with PETase-like activity, secreted by *Thermobifida fusca*, was described by Müller et al. [51] in 2005. Since then, several lipases, cutinases, and esterases have been characterized [52,53,54,55,56,57,58]. In 2016, Yoshida et al. [52] identified a bacterium named *Ideonella sakaiensis* that secretes a consortium of PET degrading enzymes—*Is*PETase and *Is*MHETase. The discovery of specific PET hydrolases reinforced the interest for microbial PET degradation and inspired the identification, characterization, and enhancement of multiple enzymes.

The growing usage of enzymes in biodegradation is partially made possible by the evolution of diverse, but complementary, scientific fields. Even though enzymes are natural catalysts, their efficiency, stability, and catalytic turnover is often insufficient to be feasibly applicable [59]. Thus, diverse enzyme engineering approaches to improve catalytic, stability and productivity properties have been developed. Ultimately, the goal of enzymatic genetic engineering is to modify the amino acid sequences through gene alteration [60].

Some of the most employed genetic engineering methods are directed evolution, DNA shuffling, saturation mutagenesis, fusion, site-directed mutagenesis, and truncation [61]. In directed evolution, no data on the protein structure and function are required, and it works by building a library of randomly generated mutants. On the other hand, in rational enzymatic design or semi-rational design, information on protein structure and function is required [60,61].

Enzymatic engineering through rational design, which implies introducing specific and pre-defined changes in the amino acid sequence by site-directed mutagenesis, has been growing as an emerging family of engineering techniques [60,62].

Since rational design implies knowledge of protein function and structure, it frequently begins with computer aided design [60]. The rapid increase of high-resolution protein structures available on the PDB database [63], and the improvement of structure-prediction tools, such as the recently released AlphaFold [64], allows for molecular and atomistic perspectives on the structure and function of enzymes. This pipeline combines computational [65] and experimental [66] techniques to improve enzymatic thermal stability (through, for example, introduction of additional disulfide bonds), filling protein voids, increase enzymatic turnover and efficiency, or attribute new functions to the enzyme.

At this stage, several PET degrading enzymes have been discovered, and as a lot of work has been done on structural solving and characterization, the emerging trend for the future of the field is the engineering and improvement of these found enzymes. Structure resolution and atomistic understanding of an enzyme is tightly related with the ability to perform rational design, and several of the efforts employed on PET enzymes follow this line of thought, as mutagenesis is used both to confirm amino acid roles, and to improve enzymatic characteristics.

This review presents an overview of the current paradigm of PET enzymatic biodegradation in academic research, with a particular structural, mutagenic, and mechanistic perspective. Special attention is dedicated to the most promising PET degrading enzymes. In addition, we provide insight into several promising enzymes with PET degrading potential, which have been the subject of preliminary characterization in recent years, but that still require further biochemical and structural studies to confirm their full potential and explore/modulate their activity. Some microorganisms with the ability to utilize PET as an energy source are also highlighted. Finally, several enzymes with polyesterase activity but no specific PET activity are also discussed.

## 2. Enzymes Involved in PET Degradation

To date, more than 24 different enzymes with PET degrading ability have been identified. All of these enzymes are hydrolases, catalyzing the breaking of the PET polymer into TPA, EG, BHET, and (mono-(2-hydroxyehyl)terephthalic acid (MHET).

Hydrolases are enzymes that catalyze the breaking of chemical bonds through a reaction with water. They constitute a particularly versatile and ample class of enzymes, with an ability to act on diverse substrates of different sizes and complexity, such as proteins, carbohydrates, lipids, and nucleic acids. Hydrolases can be characterized by an enzyme commission number EC3 and can be classified into 13 different subclasses, according to the specific types of bonds that they cleave [31]. In fact, this class of enzymes can act on extremely different bonds, with very different strengths [67]. The rates of uncatalyzed hydrolysis reactions of known substrates for hydrolases at 25 °C have been shown to span over 17 orders of magnitude, with activation free energies in solution differing from 21 to 44 kcal/mol [68]. Hence, the challenge posed by nature to the different hydrolases can be quite diverse [31], and the intrinsic stability of PET, makes its enzyme-assisted hydrolysis certainly a tough challenge. However, despite the challenge, different hydrolases present in different organisms have been shown to exhibit at least some PET degrading ability. For that, these enzymes employ a variety of strategies, some still not fully uncovered. Hydrolases involved in PET degradation act on ester bonds and are part of class 3.1 [69]. Furthermore, most PET degrading enzymes belong to the 3.40.50.1820 superfamily, according to the CATH database [70], since they share a conserved catalytic domain and assume the typical alpha/beta hydrolase fold. Breaking of PET bonds is typically accomplished by a catalytic triad involving a serine, a histidine and a negatively charged residue, usually an aspartate or a glutamate [71]. During the reaction, the product is commonly stabilized by an oxyanion hole made up of two or three residues [72].

**Figure 2:**
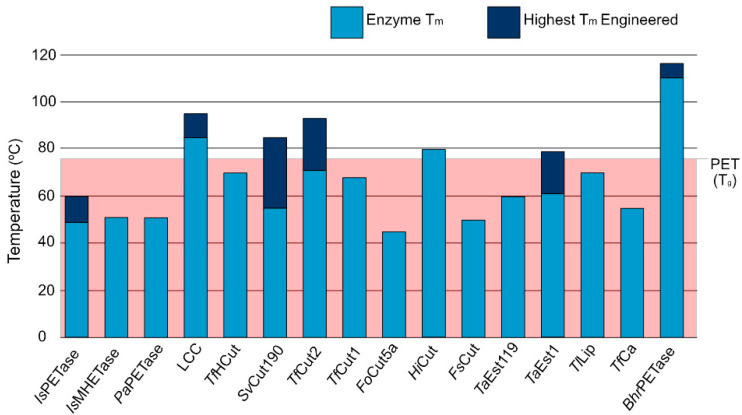
Melting temperature values for PET degrading enzymes and the highest Tm obtained through various engineering efforts in comparison with the glass temperature of PET.

The fact that PET exhibits a glass temperature (T_g_) of 76 °C, which is important to make the polymeric chain more accessible for enzymatic mediated biodegradation, constitutes an additional challenge for PET degrading enzymes, as such, enzymes must also be able to function at such high temperatures. Enzymatic melting temperature (T_m_) is the point when there is an equilibrium between protein folding and unfolding and is strongly associated with stability and catalytic ability [73]. As evidenced by Figure 2, most PET degrading enzymes exhibit T_m_ lower than the T_g_ of PET, even after engineering efforts. While enzymatic thermal stability is a complex problem, PET degrading enzymes use disulfide bonds as a stability strategy. Joo et al. [74] proposed a classification system for PET degrading enzymes that divides them in two major groups: enzymes with one disulfide bond belong to Type I, and enzymes with an additional bridge are Type II molecules. The second group is further divided into Type IIa and IIb depending on the specific amino acid composition of the enzymatic binding site. We followed this classification system when enough information on the enzyme and organism of origin was available.

**Figure 3:**
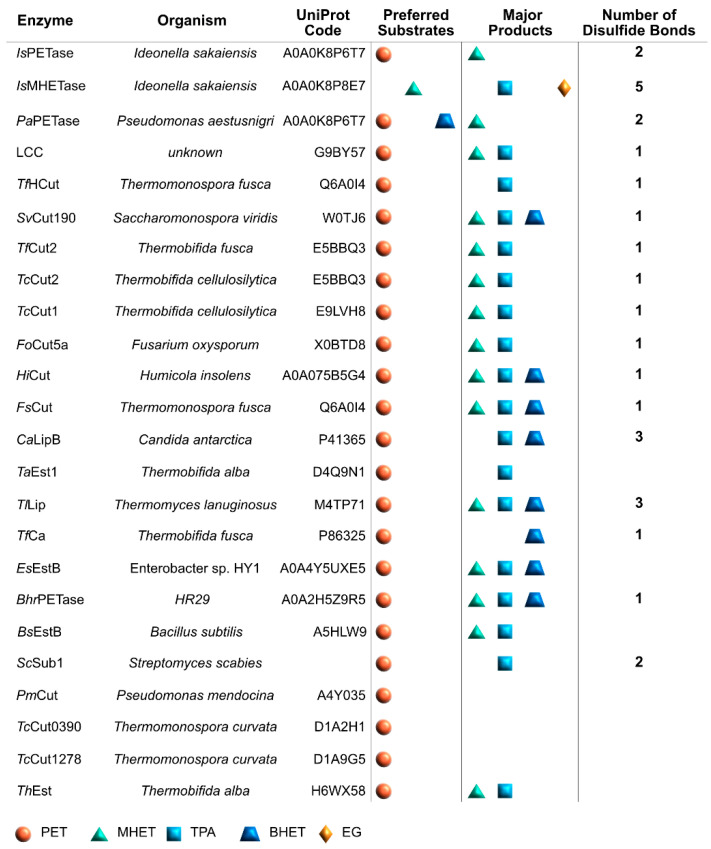
General scheme summarizing the main PET degrading enzymes, their organisms of origin, UniProt Code, confirmed substrates and major products, and number of disulfide bonds.

**Figure 4:**
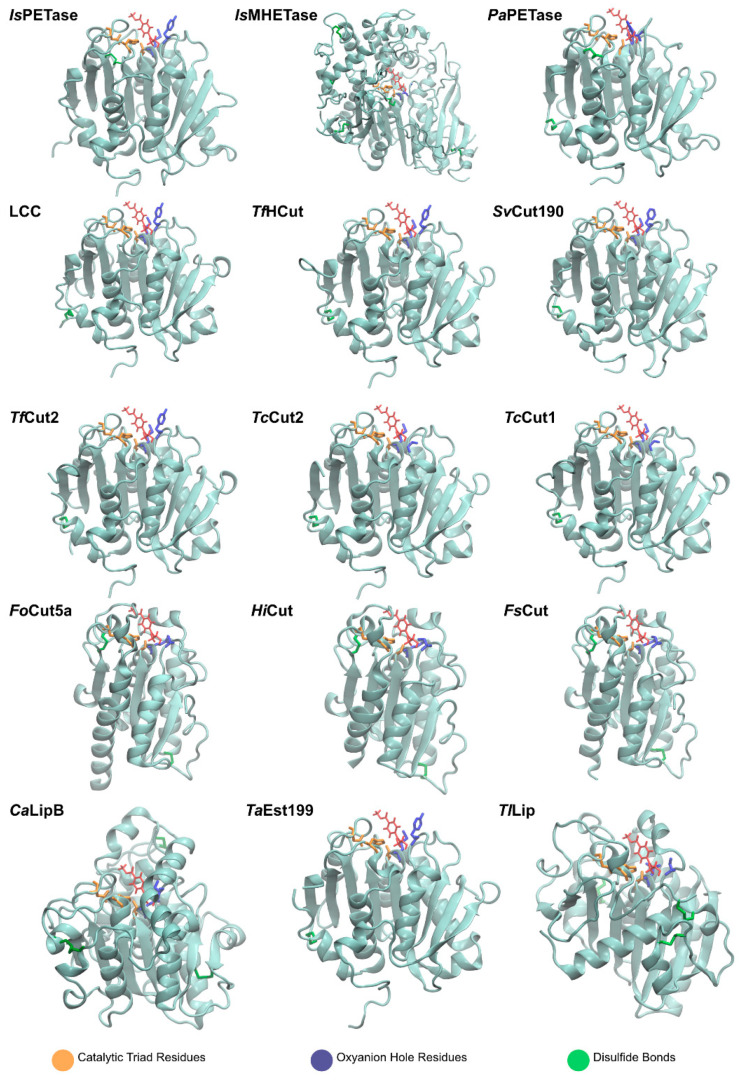
Main PET degrading enzymes known.

The graphical scheme in Figure 3 summarizes the general information known on the main PET degrading enzymes, described in detail in the following sections. Figure 4 presents a structural representation of the most important PET degrading enzymes currently known, illustrating some of the features that characterize these enzymes, such as their catalytic triads, oxyanion hole residues, and presence of disulfide bonds. These characteristics are further explored throughout this review.

### 2.1. Ideonella sakaiensis PETase (IsPETase)

#### 2.1.1. Discovery

*Is*PETase is a PET hydrolyzing enzyme first identified by Yoshida et al. [52] in 2016. This enzyme is responsible for degrading PET to MHET as a major product and EG, TPA, and BHET as secondary products [52,75]. The *Is*PETase producing bacterium, *Ideonella sakaiensis 201*–*F6*, is capable of assimilating PET as a major energy and carbon source. It was identified from a novel microbial consortium formed on PET film, isolated, and characterized as depending nutritionally on PET. Once it was confirmed that *I. sakaiensis* hydrolyzed PET to MHET, TPA, and BHET, two enzymes were identified as responsible, *Is*PETase and *Is*MHETase. *Is*PETase was determined to have higher activity against PET film and BHET than the previously identified enzymes *Tf*HCut, LCC and *Fs*Cut by 120, 5.5 and 88 times (respectively) at 30 °C and pH 7. However, activity was lower against aliphatic esters when compared with the mentioned enzymes, indicating *Is*PETase preference for PET [52].

#### 2.1.2. Structure

*Is*PETase is composed of 290 amino acid residues, exists as a functional monomer [74], and its three-dimensional structure has been explored by several structural, experimental, and computational studies [74,75,76]. The enzyme presents a canonical α/β-hydrolase fold with nine mixed β-strands that make up a central β-sheet, surrounded by seven α helixes [74]. At the active site, a conserved catalytic triad composed of a Serine (Ser160), Histidine (His237) and Aspartate (Asp206) residues is found in a broad active site cleft. Ser160 is inserted in a nucleophilic elbow with a sharp turn conformation [75] at the beginning of α4 [77]. The catalytic Asp206 residue is in the loop region between β7 and α5, while His237 is found in the loop region between β8 and α6 [77].

**Figure 5:**
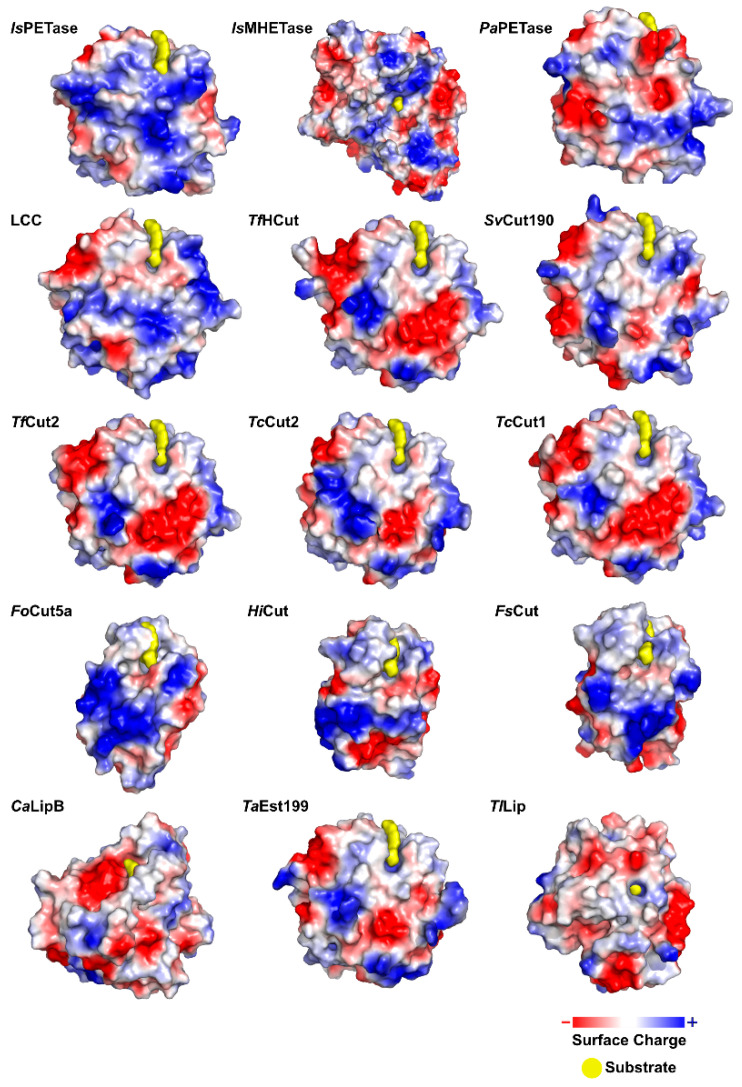
Graphical representations of the charged surfaces of the most important PET degrading enzymes.

*Is*PETase presents a highly polarized surface charge, creating a dipole across the macromolecule and resulting in an isoelectric point of 9.6 [75]. Several PET degrading enzymes present charged surfaces, as evidenced by Figure 5.

The enzyme presents two disulfide bridges: DS1 (Cys203–Cys239) and DS2 (Cys273–Cys289). DS2 is conserved amongst all known PET hydrolyzing enzymes, located near the C-terminal and distant from the catalytic center [74]. This bond does not directly intervene or contribute to hydrolysis, but has a critical role in structural integrity of the enzyme. DS1 is specific to *Is*PETase. Located near the active center, it has an essential role in catalytic activity and active site integrity. In other known PET hydrolases, this bond corresponds to highly conserved alanine residues [76]. In *Is*PETase, the bridge connects β-strand 7 to a loop connecting β-strand 8 and helix α5. This loop, anchored to the DS1 bond, contains the histidine catalytic residue. For its specificity and location, DS1 is thought to be one of the structural motifs responsible for the increased *Is*PETase activity when compared to other PET degrading enzymes [76,78].

**Figure 6:**
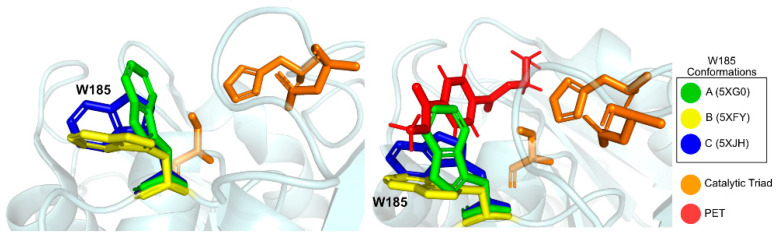
Representation of several conformations of residue Trp185, the wobbly tryptophan. Catalytic triad is represented in orange. Conformation A (PDB: 5XG0) is represented in green; conformation B (PDB: 5XFY) is represented in yellow; conformation C (PDB: 5XJH) is represented in blue. In the right side figure, HEMT, a PET model molecule (PDB: 5XH3), is represented, evidencing the optimal position of Trp185 in position C for catalysis.

A conserved Tryptophan residue (Trp185) assumes several conformations, termed A, B, and C, and is often referred to as the *wobbly* tryptophan, as represented in Figure 6. Even though the presence of this amino acid residue has been observed before in similar enzymes, its different conformations only appear in *Is*PETase, since in other known structures it always assumes the C conformation [76]. The flexibility of Trp185 was further confirmed by induced fit Docking and molecular dynamics studies [75]. In its vicinity is a serine (Ser214) residue (histidine in homologous enzymes), which allows for the A and B Trp185 conformers to be accommodated in the catalytic site [76].

Currently, there are 25 *Is*PETase structures available in the Protein Data Bank [63]. The structures, available in apo and complexed, mutated, and wild type form, are summarized and characterized in Table 1.

The first *Is*PETase structures were determined by Han et al. [76] in 2017. The first apo-structure of *Is*PETase was solved at 1.58 Å (PDB: 5XG0) and revealed the main structural findings already described. Despite several efforts, the authors were unable to obtain co-crystallized structures between wild type (WT) *Is*PETase and different ligands. For that reason, an inactive S160A (catalytic serine) and R159G double mutant was produced, and complexed structures were obtained with substrate (PDB: 5XH3) and product (PDB: 5XH2) analogues. Mutated apo-form enzyme structures were also resolved and used as a control to verify that the overall enzymatic fold and structure were unaffected by the point mutations necessary for obtaining the complexes.

**Figure 7:**
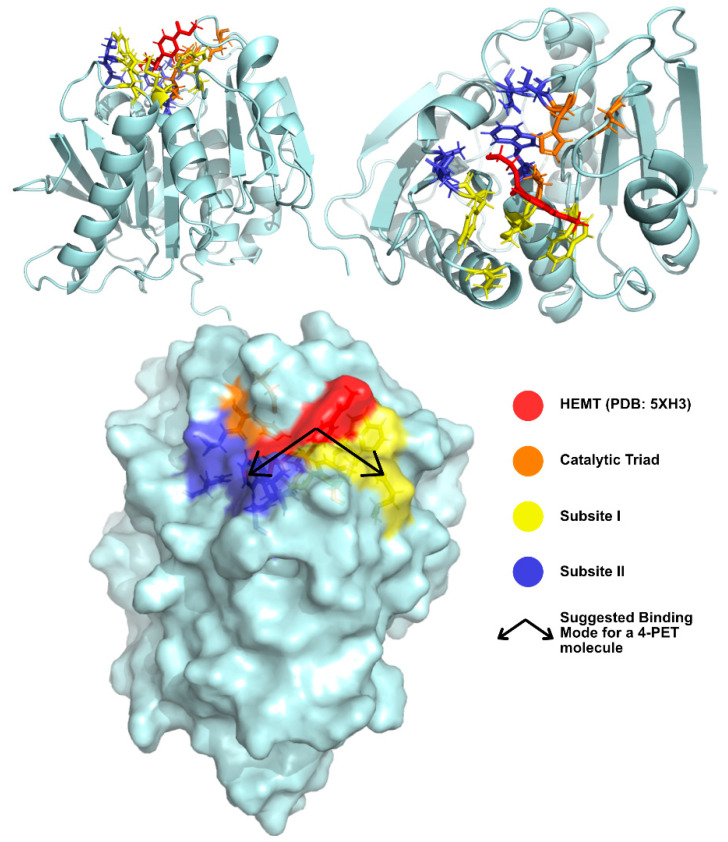
Binding Pocket of IsPETase suggested by Joo et al. [74] from different perspectives. HEMT, a PET model molecule, is represented in red. The catalytic triad is represented in orange. Binding subsites I and II are represented in yellow and blue, respectively.

Joo et al. [74] have solved and published several *Is*PETase structures since 2017 [81,83]. The group’s apo-form enzyme was solved at 1.54 Å (PDB: 5XJH) and was used to perform molecular docking studies with 2-HE(MHET)4, a 4-MHET molecule mimicking a 4-moieties PET chain [74]. The binding mode of the first moiety at the active site confirmed the presence of the conserved catalytic triad (Ser160, His237, and Asp206) and of an oxyanion hole (Tyr87 and Met161) at appropriate distances for stabilization of the reaction intermediates. The docking of a longer molecule led to the conclusion that the binding site is a mostly hydrophobic, with a long and shallow L-shaped cleft on a flat surface. Furthermore, the binding site is divided into two subsites—subsite I (defined by residues Tyr87, Gln19, Met161, and Trp185) and II (residues Thr88, Ala89, Trp159, Ser238, and Asn241), being that subsite II is further partitioned into subsite IIa, IIb, and IIc. One PET moiety binds the catalytic center in subsite I, while subsite II accommodates the remaining three moieties through mostly hydrophobic interactions. The location of the subsites in relation to the catalytic triad and the substrate are represented in Figure 7. The role of Trp185 in subsite I was evidenced by the π–π interactions between this residue and the benzene ring in PET. This stabilization of the first PET moiety is aided by Met161 and Ile208 [74].

#### 2.1.3. Activity

Several mutagenesis and activity studies confirmed the catalytic and active role of many of the amino acid residues on the structural aspects proposed. *Is*PETase engineering assays published to date are summarized in Table S1.

Given the T_g_ of PET, the polymer is more readily available for enzymatic degradation at higher temperatures [84]. However, the melting temperature (T_m_) of *Is*PETase was determined at 48.81 °C [81]. For that reason, engineering of PETase to increase enzymatic activity should be paired with engineering efforts to raise thermal stability and durability [77].

Mutagenesis of the catalytic triad to alanine residues (S160A, H237A, and D206A) consistently resulted in total loss of enzymatic activity, confirming their critical essential role as the catalytic triad [74,78]. Disulfide bridge DS1 disrupting mutations (C203S and C239S) resulted in very low enzymatic activity and lowered T_m_ by over 10 °C, confirming the essential role of the *Is*PETase specific bridge in both activity and stability [74,76]. Most studies targeting the oxyanion hole and substrate interacting residue Tyr87 with various substrates (PET film, PET bottle and BHET monomers) reported diminished activity and lower amounts of product released [74,76,83], although in one study, a slight activity increase is reported [78]. Engineering of the other oxyanion hole amino acid residue Met161 consistently resulted in lower PETase activity against all tested substrates [74,76,78,83]. The *wobbling* Trp185 has also been the target of engineering studies—replacement of this residue with an alanine results in highly diminished to total loss of activity [74,75,76,78]. Residue Ser214, thought to influence Trp185 flexibility, has been engineered to a histidine residue (similar to other enzymes with PET hydrolytic activity), in two different studies. Results were non-consensual, since the mutation has both led to partly compromised activity [76] and slightly increased activity [78], depending on the study.

Several studies focused on engineering substrate binding residues in an attempt to increase enzymatic degradation activity. Han et al. [76] mutated Ile208, Trp159, Thr88 to alanine residues, with consistent decreases in PETase activity, showing the importance of these residues in catalysis. These findings were also corroborated by Joo et al. [74].

In addition, Joo et al. [74] explored residues from the binding subsite II (W159A, S238A, N241A) that resulted in activity reductions. To simulate *Tf*Cut2 binding site, Ser238 was mutated to phenylalanine (S238F) and Trp159 to histidine (W159H), with drastic consequences to *Is*PETase hydrolytic activity. The observation that Arg280 (located in subsite IIc) presented a protruding shape that seemed to destabilize the binding of PET led the authors to produce a R280A variant. This mutant presented augmented PET degrading activity against PET film by 22.4% in 18 h and 32.4% in 36 h when compared with the WT enzyme. The resolved structure (PDB: 5YNS) showed that alanine provided a hydrophobic and non-protruding cleft, resulting in an extended subsite IIc. Interestingly, even though Arg280 is ~23 Å away from the catalytic center, this mutation highly affected PETase activity, since it resulted in structural changes that led to better substrate accommodation. This observation suggests that key structural changes away from the active site have the potential to impact enzymatic activity. This finding inspired the authors in subsequent mutagenic studies [81,83].

In 2019 [81], the group published a manuscript applying the same rational protein engineering strategies to explore different mutations, using PET film as a substrate. Residue Pro181 disrupts the enzyme’s secondary structure, suggesting a negative effect on enzymatic thermal stability and catalytic efficiency. Therefore, the variant P181A was produced, and resulted in diminished activity at both 30 °C and 40 °C, besides having a higher T_m_ value than WT *Is*PETase by 0.5 °C. After resolving and inspecting the P181A *Is*PETase structure (PDB: 6IJ5), it was verified that the mutation led to a collapse of the catalytic site since it shifted catalytic Asp206 away from the His237 residue. The authors, inspired by the structure of *Tf*Cut2, an enzyme with higher thermal stability than PET, designed a S121D/D186H double mutant to increase the stability of the β6–β7 connecting loop since these residues were thought to form an additional hydrogen bond. Indeed, the T_m_ of this variant was of 54.85 °C, 6 °C higher than the T_m_ of WT enzyme. This mutation resulted in an activity increase of 2.3- and 2.0-fold after 24 and 72 h, respectively, at 30 °C, and a 3.4- and 4.4-fold after 24 and 72 h, respectively, at 40 °C. The effect on enzymatic activity is likely due to the increased thermal stability, which allows the enzyme to remain active longer at higher temperatures. *Is*PETaseS121D/D186H, inspired by these findings, was designed to be even more stable—which proved to be true, since this variant had a measured T_m_ of 56.02 °C and an increased enzymatic activity of 2.2- and 2.6-fold after 24 and 74 h, respectively, at 30 °C, and 4.7- and 6.0-fold at 24 and 72 h, respectively, at 40 °C. Even though these variants increased PETase activity and stability by a fair amount, the authors combined these findings with the previous ones and designed two triple mutants—S121D/D186H/R280A and S121E/D186H/R280A. The measured T_m_ for these variants were of 56.41 and 57.62 °C, 7.6 and 8.81 °C higher than WT *Is*PETase. The activity of these variants was also higher than those of previously reported enzymes. S121E/D186H/R280A showed a 4.3 and 5.2 higher fold enzymatic activity after 24 and 72 h, respectively, at 30 °C, and a 9.1 and 13.9 higher fold after 24 and 72 h, respectively, at 40 °C.

Recently, the same group published a third mutagenic study on *Is*PETase [83]. After performing molecular docking experiments with a 2-HE(MEHT)_4_ molecule, several point mutations on substrate binding residues were designed, inspired by conserved and observed residues in other PET hydrolytic enzyme candidates. Most mutations led to slightly (Y87F, I208V, S238T) or drastic (T88L, R90S, I208T, G234N) activity decreases. However, mutations S242T and N246D resulted in significant activity increase. For that reason, efforts to incorporate these mutations into the previously designed triple mutants were developed and a S121/D186H/N246D/280A variant was designed. Unfortunately, this mutated *Is*PETase exhibited a decrease in activity and thermal stability when compared with the previously reported variant. After structural studies, it was discovered that the R280A and N246D mutations were not compatible and could not be employed simultaneously. Therefore, triple mutants incorporating each of these variants were designed, and the results revealed that N246D was the more beneficial mutation for enzymatic activity and thermal stability. For that reason, a quadruple S121E/D186H/S242T/N246D mutant was produced (PDB: 6KUS). Both the T_m_ and hydrolytic activity of these variant were higher than those of previous enzymes. This variant showed a gradual increase of PETase degradation activity up to 20 days, meaning a 58-fold higher activity than WT *Is*PETase at 37 °C. These results are remarkable and extremely promising—a true testament to the potential of rational protein design.

Austin et al. [75] produced a S238F/W159H double mutant with the goal of converting the *Is*PETase binding site into a cutinase-like active site cleft. *Is*PETaseS238F/W159H resulted in higher enzymatic activity (evidenced by an increase in product release) and crystallinity reduction against PET film when compared with WT enzyme. To understand the increase in activity, the authors performed induced fit docking and theorized that the introduced Phe238 performed stabilizing aromatic interactions with the substrate, as well as with other amino acid residues in the active site.

The best performing *Is*PETase variant published to date was developed by Cui et al. [82] through a novel computational strategy termed GRAPE (greedy accumulated strategy for protein engineering). GRAPE employs a strategy for optimization of mutations in a cluster manner, creating several functional variants and selecting the most promising ones [82]. It is a rational and efficient way to combat the tardy strategy of exploring point mutations individually and combining them in various manners, requiring experimental testing at each stage. Initially, the algorithm identified 21 stabilizing mutations; however, a variant containing all of these mutations proved to be inactive, indicating some of them were not compatible when employed simultaneously. Then, a clustering method (*K-means algorithm*) was used to generate promising combinations of mutations. Several variants were tested, but DuraPETase (S214H/I168R/W159H/S188Q/R280A/A180I/G165A/Q119Y/L117F/T140D) was by far the most successful [82]. It had been shown that WT *Is*PETase suffers total loss of activity within 24 h at 37 °C [81]—DuraPETase, having a much higher T_m_ and, therefore, thermal stability, resulted in high activity for longer periods at higher temperatures. Besides exhibiting a 300-fold activity increase in 10 days incubation with crystalline PET at 37 °C, the variant was active and functional for up to 3 days incubation at 60 °C [82], which is a remarkable achievement. The variants’ ability to degrade nano and microplastics, one of the major issues for marine environments [85], was also much higher than that shown by WT *Is*PETase. DuraPETase also presents less specificity than the WT enzyme and can degrade other plastics such as polybutylene terephthalate (PBT) and polyethylene 2,6-naphthalenedicarboxylate (PEN) [82]. The authors solved the three-dimensional structure of DuraPETase (PDB: 6KY5) at 1.63 Å to understand the mechanistic and structural attributes responsible for the enhanced activity. The overall structure of the variant is similar to WT *Is*PETase, and so was the PET binding mode predicted through molecular docking studies. The features highlighted as responsible for the increased hydrolytic activity were novel electrostatic interactions (T140D, I168R, W159H, S188Q), improved hydrophobic packing (Q119Y, A180I, S214H, R280A, L117F), reduction of conformational entropy (G165A). L117F and Q119Y provided additional stabilization of the typical binding mode, similar to the role performed by Trp185, Met161, and Tyr87 in the WT enzyme [82].

Liu et al. [34] tested *Is*PETase enzymatic activity under different conditions. Three-point mutations (S93M, W159F, and N241F) were shown to increase enzymatic degradability of *p-*nitrophenyl esters, contrary to the highly specific WT enzyme. These mutations change the hydrophobicity and reduce the steric effects responsible for enzymatic specificity. *Is*PETase typically performs best under mild temperature conditions (35 °C) and loses stability as the temperature increases, accompanied by a decrease in activity. Immobilization of the enzyme by ammonium sulfate precipitation and glutaraldehyde cross-linkage resulted in maximum activity from 35 to 45 °C and 60% of activity at 65 °C. Regarding physicochemical factors, *Is*PETase was most active in buffer containing 20% glycerol and its activity increased as the buffer salt concentration increased. Activity was proportional to salt concentration from 100 to 500 mM. Na_2_SO_4_ was the highest impacting salt on enzymatic activity. However, *Is*PETase was inhibited by organic solvents, protease inhibitors, and detergents typically used in the industry and laboratory, such as propanol, ethanol, SDS, Tween 20, PMSF, and Triton X-100. The activity increase under high salt concentrations was suggested to most likely be due to a potential cation binding-site and anion-binding site in the enzyme [56].

In order to enhance *Is*PETase activity and stability, Chen et al. [86] built a whole cell biocatalyst of PET functionalized on the surface of *Pichia pastoris* yeast cell. Assays with PET film for 18 h at pH 9 and 30 °C resulted in a 36-fold increase in activity when compared with individual *Is*PETase. Activity was determined by measuring the produced amounts of MHET product. Furthermore, the complex system performed at a stable turnover rate for seven repeated uses under the same conditions, and was further tested with commercial highly crystalline PET bottles, having shown degradation ability. This study presented a promising direction for *Is*PETase activity increase and wider industrial applicability.

Inspired by the advantages of using microalgae for expression and production of plastic degrading enzymes, Moog et al. [87] employed *P. tricornutum* for *Is*PETase production and Kim et al. [88] used *C. reinhardtii* microalgae. In both studies, *Is*PETase activity with morphological changes on PET substrate and production of PET degradation metabolites such as TPA was observed. These studies suggest that using microalgae for *Is*PETase expression and introduction in the environment is an attractive and promising strategy, especially given the natural characteristics of microalgae—their abundance and low maintenance growth in natural aqueous systems with no endotoxin production [88].

#### 2.1.4. Proposed Mechanism

**Figure 8:**
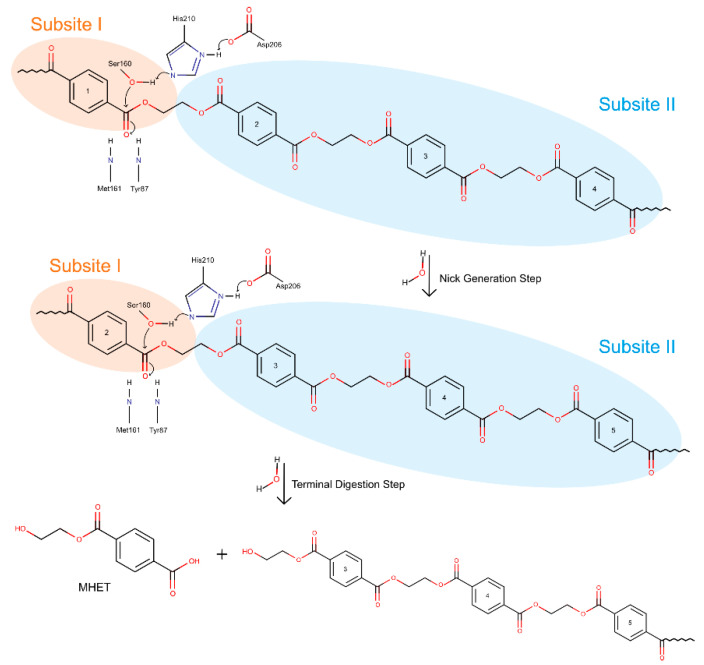
Scheme mechanism of IsPETase.

The structural and mutagenic findings have led to a catalytic mechanism proposal for PET degradation by *Is*PETase. It is consensual that the mechanism follows a classical serine hydrolase family mechanism, similar to cutinase [89]. The only difference seems to be the role of Trp185, the *wobbly* tryptophan near the catalytic center. The movement of this residue is thought to have an essential role in both substrate binding and product release [90]. The current main mechanistic proposals are presented by the same groups that resolved the first known *Is*PETase three-dimensional structures. While Han et al. [76] focused on a detailed proposal on the role of each amino acid residue in the catalytic triad and the movements of atoms and electrons, Joo et al. [74], inspired by their findings on the enzyme’s binding site, proposed a broader mechanistic view, considering the several possible products of PET hydrolysis. Since both proposals fit together to yield a complete mechanism, they will be integrated and explained simultaneously in this work and represented in Figure 8.

The presently accepted mechanism initiates with the binding of *Is*PETase to the substrate. The shallow cleft on apo-form enzyme allows for substrate binding onto the protein surface [74,76]. This is the beginning of the nick generation step, in which four moieties bind the protein—one to subsite I, where the catalytic reaction takes place, and the remaining three onto subsite II [74]. Upon binding, the cleavable ester bond is in optimal position to be attacked by Ser160 and stabilized by the oxyanion hole (Met161 and Tyr87) [76]—this cleavage leads to a nick in PET, resulting in two PET chains with distinct terminals released from each subsite: a TPA-terminal from subsite I and a hydroxyethyl terminal (HE terminal) from subsite II [74].

The considered mechanistic proposal suggests the first step to be a charge-relay system between catalytic residues Asp206 and His237. This system allows His237 to deprotonate the hydroxyl group on Ser160, which becomes a stronger nucleophile and attacks the carbonyl group of the PET ester [77]. The attack initiates catalysis and leads to the formation of the first tetrahedral intermediate, stabilized by the oxyanion hole. The intermediate’s negative charge is highly unstable, and breaks down, resulting in the release of the first product and in an acyl-enzyme intermediate [76,77]. A second nucleophilic attack by a water molecule (deprotonated by the same His237–Asp206 charge-relay system) to the carbonyl carbon results in the second tetrahedral intermediate. Equally unstable, the intermediate breaks down and releases the second product. This step finishes with the full regeneration of the catalytic triad, so that another catalytic cycle can begin [76,77].

In the terminal digestion step, the two cleaved chains are digested into MHET (and BHET, TPA, and EG, in residual amounts) in different ways depending on the terminals of each molecule. For the HE terminal PET, once the four moieties were bound to subsite I and II, the already described ester bond breakage results in the production of a MHET monomer (first product released) and a HE-PET(n-1). The digestion of this HE-PET molecule follows the same steps as the first ester bond cleavage process [74]. The TPA-terminal PET molecule positions itself in the binding site with the TPA terminal at subsite I and the remaining three PET moieties in subsite II. In this case, the cleavage of the ester bond produces one TPA molecule (first product) and a HE-PET(n-1), which suffers a similar breakage as described previously [74]. The different bindings and terminals generated result in a series of PET monomers and dimers that are eventually digested to the final products in a combinatorial manner, leading to the accumulation of four molecules—MHET, TPA, EG, and BHET. Finally, BHET can be degraded into MHET and EG, and the final products of *Is*PETase activity are MHET, TPA, and EG [74].

The binding mode proposed by Joo et al. [74], of 4-MHET moieties binding *Is*PETase simultaneously, was recently contested by Wei et al. [91]. The authors performed NMR analysis of amorphous PET at 30 °C and observed that the polymer does not acquire the conformation necessary to fit the docking predictions presented by Joo et al. [74] and, therefore, reject the binding mode and the overall mechanistic proposal based on it. Furthermore, Wei et al. [91] performed comparative activity assays with *Is*PETase and LCC at their respective optimal activity temperatures (30 °C and 70 °C, respectively) for 24 h and observed a 40-fold higher weight loss of PET material with LCC when compared with *Is*PETase. This suggests LCC activity on PET is much higher than that of *Is*PETase, which is in disagreement with the original activity reports by Yoshida et al. [52]. Recently, Joo et al. [92] responded to Wei et al.’s [91] publication, arguing that the docking experiments did not consider temperature and, therefore, it is possible that the 4-MHET moiety polymer acquires the proposed conformation at higher temperatures. Moreover, the authors argued that even though *Is*PETase has a low thermal stability, several variants with higher melting temperatures have resulted in increased activity, which means that at higher temperatures the proposed binding mode and mechanism are plausible. Even though it is true that at higher temperatures amorphous PET content increases and, consequently, so does conformational freedom, it now seems plausible that the proposed binding mode and mechanism are neither likely nor favorable at a 30 °C temperature. This apparent low availability of PET chains at lower temperatures might also be the reason for the several reports on lower *Is*PETase activity than originally described by Yoshida et al. [52]. Similarly, Kawai et al. [93] questioned the classification of *Is*PETase as a PET degrading enzyme, arguing that PET degradation implies that the amorphous region of the polymer be attacked first, and only then the crystalline region becomes susceptible to hydrolysis. Therefore, since at 30 °C the amorphous content of PET polymer is residual, *Is*PETase would not be able to attack the polymer and degrade it in the described manner. Although further and more expansive studies on *Is*PETase activity on different PET substrates and at different temperatures are necessary, it seems too drastic to claim no PET hydrolytic activity can be attributed to *Is*PETase, given the exhaustive and multiple studies published, and hereby summarized on this enzyme. In conclusion, *Is*PETase activity, mechanism of action, and power is still not fully consensual, and future studies will be important to clarify some of the issues raised in recent years regarding *Is*PETase potential.

#### 2.1.5. Future Perspectives

At this stage, knowledge about th *Is*PETase structure, function, and mechanism is quite extensive. Moreover, with the increasing need for PET degrading alternatives, new studies on this enzyme continue to appear. Several studies have resulted in X-ray structures of *Is*PETase with different variations, in terms of mutations and bound-molecules, and proposed what specific characteristics led to the augmented *Is*PETase enzyme when compared with other PET hydrolases. However, and even though several engineered *Is*PETase variants present higher T_m_ values and preserve activity at higher temperatures, and for longer, these variants are still far from being usable in industrial contexts. Furthermore, the recent doubts cast on *Is*PETase activity and mechanism posed by Wei et al. [91] and Kawai et al. [93] highlight the need for additional analysis of this enzyme’s activity and potential as an effective PET hydrolase. Even so, in line with the generally accepted activity reports, the strategies developed by Son et al. [83] and Cui et al. [82] constitute a promising methodology for the development of more active and durable plastic degrading enzymes. Furthermore, the non-engineering strategies explored by Liu et al. [34] represent an interesting preliminary evaluation of *Is*PETase as a broader plastic degrading enzyme and introduces valuable information on its performance in industrial settings.

### 2.2. Ideonella sakaiensis MHETase (IsMHETase)

#### 2.2.1. Discovery

*Is*MHETase is the second PET hydrolyzing enzyme identified in *Ideonella sakaiensis 201*–*F6* by Yoshida et al. [52] in 2016. It is an intracellular enzyme with a molecular weight of 65 kDa and 603 amino acids. According to ESTHER database, *Is*MHETase is labeled as a tannase that belongs in the α/β-hydrolase Block X family [94]. This superfamily is comprised not only by fungal and bacterial tannases, but also by feruloyl esterases. Its properties as a PET biocatalyst were firstly described by Yoshida et al. [52] in 2016, when it was shown to degrade MHET to EG and TPA, catalyzing the subsequent step of PET degradation initiated by *Is*PETase. Later studies confirmed that *Is*MHETase had no activity on BHET [77].

#### 2.2.2. Structure

**Figure 9:**
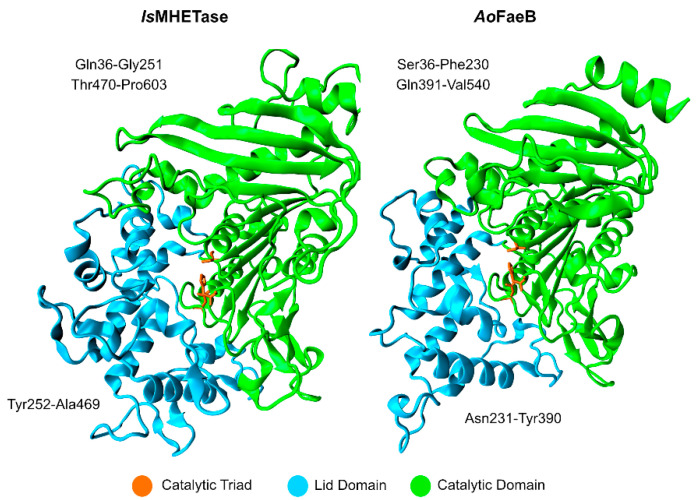
Lid and catalytic domains of IsMHETase in comparison with those of AoFaeB. The catalytic triad for each enzyme is represented in orange.

Studies on the *Is*MHETase structure show that it exists as a monomer [95], where the catalytic domain adopts an α/β-hydrolase fold, similar to serine hydrolases, and the lid domain is larger than the average lid domain of α/β-hydrolases. *Is*MHETase lid domain is composed of ~240 amino acid residues, while the average length in α/β-hydrolases is about ~100 residues, as evidenced by Figure 9. The lid domain partially involves the active site and a Ca^2+^ binding-site, similar to *Aspergillus oryzae* FaeB (*Ao*FaeB), known to increase lid domain stability [96].

Contrary to *Is*PETase, *Is*MHETase has a more heterogenous and acidic surface, resulting in a lower isoelectric point (5.11) [89]. The enzyme contains five disulfide bonds (Cys51–Cys92, Cys224–Cys529, Cys303–Cys320, Cys340–Cys348, and Cys577–Cys599), which are conserved in tannase family members [89]. The one binding Cys224 to Cys529 is in the active site and flanks the catalytic triad, which is formed by Ser225, His528, and Asp492, and the oxyanion hole, which is composed of the backbone amide nitrogen atoms of Gly132 and Glu226. Due to the properties of disulfide bonds, it is most likely that this bond tightens the catalytic triad, increasing *Is*MHETase stability.

Currently, there are nine three-dimensional structures of *Is*MHETase, including the WT enzyme in complexed and apo-form. These structures are summarized in Table 2.

The first crystal structures of *Is*MHETase were determined in 2019 by Palm et al. [80]. The authors showed that *Is*MHETase binds to MHETA, a non-hydrolyzable substrate analogue of MHET (PDB: 6QGA), through hydrophobic contacts between the phenyl ring and α/β-hydrolase residues Phe495, Gly132, and Ala494. In a similar way, the lid domain residues Phe415, Leu254, and Trp397 also establish hydrophobic contacts with the phenyl ring. Further studies confirmed the same binding mode with MHET [89,95]. Phe415 undergoes an induced-fit conformational change, pointing in the opposite way (open position) from the active site when the enzyme is in its free form; thus, promoting substrate binding and pointing towards the active site (closed position) when the enzyme is bound to a substrate. Arg411, which is sustained by Ser416, Ser419 and the backbone amide of Gly258, connects the two oxygens of the MHET free carboxylate.

**Figure 10:**
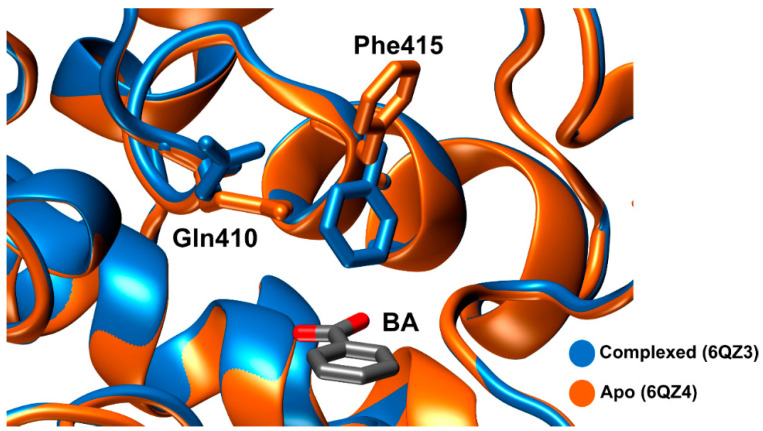
Schematic representation of Phe415 and Gln410 conformational change upon binding to benzoic acid (BA). Apo-form is represented in orange (PDB: 6QZ4) and the complexed form is represented in blue 8PDB: 6QZ3).

Lastly, Knott et al. [89] solved four structures. Here, the group demonstrated that the carboxylate motif of MHET establishes hydrogen bonds with Arg411 and Ser416. Just like Phe415, Gln410 also shows a concerted movement, where the side chain pivots towards the active site, when Phe415 is pointing in the opposite way from the active site, as can be seen in Figure 10.

#### 2.2.3. Activity

*Is*MHETase has a high affinity and activity towards the substrate MHET, resulting in a K_M_ and k_cat_ of 7.2 µM and 27.6 ± 2.6 s^−1^ [75,89]. However, the enzyme is incapable of efficiently degrading the other PET intermediate BHET, resulting in a k_cat_ of 0.0011 ± 0.0002 s^−1^ [80]. The substitution of the catalytic triad by alanine mutants resulted in total loss of enzymatic activity, confirming their essential role in catalysis [80,89]. The oxyanion hole residue Glu226 was mutated to threonine (E226T). The variant resulted in a ~50% activity reduction [89]. Having five disulfide bonds, *Is*MHETase was expected to have a relatively high thermostability. However, when analyzing *Is*MHETase melting temperature, Sagong et al. determined that its T_m_ value was only 50.61 °C.

Several engineering efforts to increase *Is*MHETase performance and stability were conducted. The amino acid residue Trp397, reported to be involved in MHET substrate binding, was replaced by the hydrophobic residue alanine (W397A). The resulting variant manifested an increase of enzymatic activity at high substrate concentration towards MHET and MpNPT (mono-4-nitrophenyl terephthalate) and a slight decrease of substrate affinity of MpNPT [80]. Arg411, reported to establish hydrogen bonds with substrates was replaced by alanine (R411A), positive charged amino acid lysine (R411K) and polar amino acid glutamine (R411Q). As expected, the variants R411A and R411Q have low affinity and activity towards MHET and MpNPT. However, R411K variant has a 1.7-fold activity increase for BHET as a substrate [80,95].

The residue that undergoes induced-fit conformational change, Phe415, has been mutated to alanine, serine, and histidine (F415A, F415S, and F415H, respectively). Interestingly, the variant F415H exhibits an increased turnover rate towards MHET and has no effect on MpNPT, while F415A resulted in a lower turnover rate and affinity towards MHET and MpNPT and F415S resulted in a lower hydrolysis activity towards MHET [80].

Phe424 is a residue that is located at the inner substrate-binding site and is potentially hindering the optimal BHET binding. Thus, mutagenesis was applied replacing the residue for glutamine, asparagine, histidine, aspartate, glutamate, threonine, valine, leucine, isoleucine, alanine, and serine (F424Q, F424N, F424H, F424D, F424E, F424T, F424V, F424L, F424I, F424A, and F424S, respectively). Overall, all variants resulted in low turnover rates against MpNPT and MHET and, as expected, higher turnover rates and activity towards BHET, where F424N, F424V, F424I, and F424Q display better results. The possibility of *Is*MHETase having a catalytic tetrad was ruled out when the variant His488 resulted in an unaltered turnover rate [80].

Knott et al. [89] have an interesting approach when it comes to the lid domain and the disulfide bond presented in *Is*MHETase. The lid domain of *Is*MHETase (Gly251–Thr472) was removed and replaced by the *Is*PETase loop residues (Trp185–Phe191), which possibly confer activity against PET. However, the resulting enzyme was unable to degrade PET and the turnover rate value towards MHET was 1000-fold lower when compared to WT *Is*MHETase. The active site disulfide bond (Cys224–Cys529) was then removed from similar lidless *Is*MHETase variants, being replaced with tryptophan and serine (C224W–C529S) or histidine and phenylalanine (C224H–C529F). These variants conferred almost a total loss of activity against MHET, being the k_cat_ values 0.10 ± 0.06 s^−1^ and 0.06 ± 0.03 s^−1^, respectively. Lastly, the group included disulfide bonds from PETase-like (G489C/S530C) and *A*oFaeB, giving *Is*MHETase a total of seven disulfide bonds. The resulting variant had a very low activity towards MHET (k_cat_ = 0.16 ± 0.14 s^−1^). More variants were tested but failed to express.

Besides the described assays, many other engineering efforts have been done on MHETase, as summarized in Table S2.

#### 2.2.4. Proposed Mechanism

A proposal for the catalytic mechanism of *Is*MHETase was made by Knott et al. [89], based on quantum mechanical/molecular mechanical (QM/MM) calculations with 2D umbrella sampling.

The structures determined by the group suggest that the best fitting mechanism for *Is*MHETase hydrolysis is the one characteristic of the serine hydrolase enzymes. According to this proposal, catalysis involves a two-step reaction, where the formation of an acyl-enzyme intermediate (acylation) occurs first, and is followed by its hydrolytic release. For acylation, His528 is thought to deprotonate Ser225, which becomes a nucleophile and attacks the carbonyl carbon of MHET, resulting in the liberation of EG. This exits the active site within 4 s of the formation of the acyl-enzyme intermediate (AEI). The minimum free-energy path (MFEP) calculated from the C–O bond that was formed between the MHET carbonyl carbon and Ser225 and the broken MHET C–O bond, predicts an acylation free-energy barrier (ΔG_‡_) of 13.9 ± 0.17 kcal/mol and an overall reaction free energy (ΔG_r_) of −5.2 ± 0.04 kcal/mol. The departure of EG allows a better access for water molecules to interact with charged His528, marking the start of the second step. AEI is subjected to nucleophilic attack by a water molecule, where His528 deprotonates the catalytic water and transfers the proton to the catalytic Ser225, regenerating the former for a new catalytic cycle, thus releasing TPA. The MFEP calculated from the C–O bond that was formed between MHET and water and the broken AEI C–O bond predicts a diacylation ΔG_‡_ = 19.8 ± 0.10 kcal/mol and ΔG_r_ = 2.60 ± 0.07 kcal/mol. This step is thought to be the rate-limiting step, with a k_cat_ of 7.1 ± 1.1 × 10^−2^ s^−1^, while for acylation it is about 1.02 ± 0.28 × 10^3^ s^−1^ [89]. Overall, the reaction is exergonic (−2.60 ± 0.08 kcal/mol).

#### 2.2.5. Future Perspectives

Many key factors need to be studied for a better understanding of *Is*MHETase. The enzyme’s thermal stability is significantly low when compared with the known necessary values in the industry of plastics, and engineering efforts to solve this issue are essential for further development. Furthermore, confirmation of the catalytic mechanism is needed, which provides needed information for the application of mutagenesis, and lastly, development of new mutant variants is needed to increase the hydrolytic activity of *Is*MHETase to the plastic-degrading industry standard levels.

### 2.3. Pseudomonas Aestusnigri PETase (PaPETase)

#### 2.3.1. Discovery

*Pa*PETase, commonly known as PE–H, is a PET hydrolase produced by the bacterium *Pseudomonas aestusnigri,* first identified by Bollinger et al. [97] in 2017. *P. aestusnigri*, a gram-negative rod shaped bacteria, is a novel species isolated from a marine area affected by a large oil spill in the last decade [98]. Motivated by the evidence that these bacteria had polyester degrading activity [99], the bacterial genome sequence [100] revealed a likely hydrolase coding gene [101] that coded for functional PET hydrolase. Wild type *Pa*PETase actively degrades amorphous PET film and BHET to MHET, with no production of TPA.

#### 2.3.2. Structure

**Figure 11:**
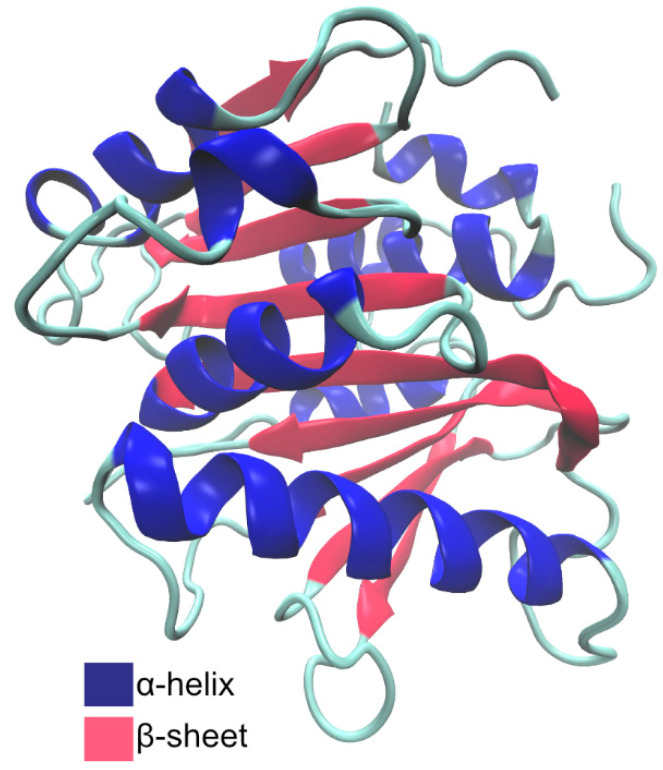
Schematic representation of PaPETase secondary structure motifs.

*Pa*PETase is composed of 304 amino acid residues, with a total molecular weight of 32 kDa, plus a signal peptide of 25 amino acids. Bollinger et al. [97] solved two *Pa*PETase structures, summarized in Table 3. The structure revealed a functional monomer with a conserved α/β-fold composed of a nine β-strand central twisted β-sheet and seven α-helices on both sides, as represented in Figure 11.

**Figure 12:**
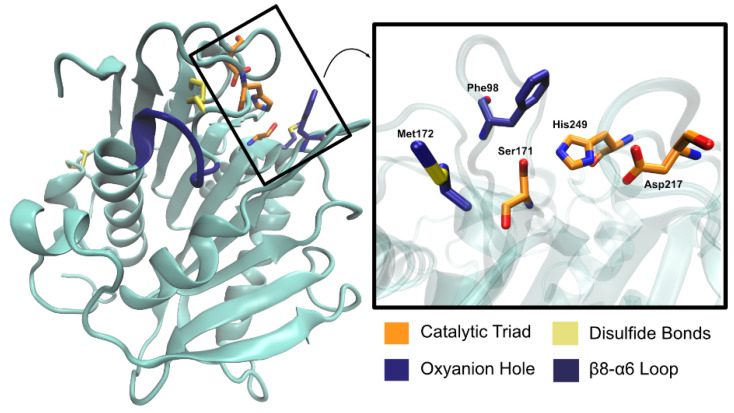
Relevant PaPETase amino acid residues. The catalytic triad, oxyanion hole, and disulfide bond residues are represented in licorice, while the loop is represented in new cartoon.

The catalytic triad (Ser171, Asp217, and His249) is found right below the surface, with Ser171 occupying the traditional position in the nucleophilic elbow. An oxyanion hole composed of Met172 and Phe98 stabilizes the substrate during the reaction. The enzyme has two disulfide bonds—Cys214–Cys251 and Cys285–Cys302, as is common for type II PET hydrolyzing enzymes. Characterization of *Pa*PETase as a Type II PET hydrolase is due to the additional disulfide bond and novel amino acid residues near catalytic histidine residue, not present in Type I PET hydrolyzing enzymes, which are typically cutinases. An extended loop region connecting β8–α6 like the one observed in *Is*PETase [74] was identified in *Pa*PETase and is defined by residues 254–259. The amino acid content of this loop defines this enzyme as a type IIa PET hydrolytic enzyme. These relevant structural aspects are represented in Figure 12.

#### 2.3.3. Activity

Bollinger et al. [97] verified that WT *Pa*PETase degraded BHET and amorphous PET to MHET, with almost no TPA production registered. *Pa*PETase degradation of PET resulted in 4.2 (±1.6) mg/L MHET after 40 h at 30 °C. No hydrolysis on commercial bottle film PET was observed.

*Pa*PETase and *Is*PETase are both defined as Type II PET hydrolyzing enzymes. However, *Pa*PETase is further classified as a type IIa enzyme, while *Is*PETase belongs in group IIb. This distinction is due to the amino acid content of a structurally conserved extended loop region made up of six amino acid residues. In *Pa*PETase, these are Gly254, Gly255, Ser256, Ile257, Tyr258, and Asn259. On the other hand, the *Is*PETase loop is made up of Ser242, Gly243, Asn244, Ser245, Asn246, and Gln247. Besides, Tyr250 was identified as the equivalent *Pa*PETase residue to *Is*PETase Ser238, a relevant amino acid in *Is*PETase catalysis. *Pa*PETase variants containing single-point and entire loop replacements in these positions with *Is*PETase residues were produced and tested for activity against BHET and PET. Variants G254S, Y258N, N259Q, and the full loop mutation resulted in significantly decreased activity and lower T_m_ than WT enzyme by 5–10 °C. *Pa*PETase S256N, I257S, and also resulted in diminished activity but less drastic lowering of T_m_, by 1–3 °C. Only Y250S resulted in higher activity against *p*NPB, BHET, and PET, resulting in a higher production of MHET than wild type enzyme. Particularly, variant Y250S yielded 5.4 (±0.6) mg/L of MHET after 48 h at 30 °C, higher than the 4.2 mg/L produced by WT enzyme. Variants Y250S and S256N were active on film PET derived from a commercial bottle, unlike WT *Pa*PETase enzyme, although with a relative low yield.

Structure determination of variant Y250S (PDB: 6SCD) revealed a more accessible and spacious active site cleft when compared with WT *Pa*PETase (PDB: 6SBN). Two loop regions (the loop connecting β3–α2, composed of residues 98–104, and loop connecting β4–α3, made up of residues 123–128) are mostly responsible for these differences. In the WT enzyme, these loops are parallel to each other in a “closed” conformation, while in the engineered variant they are shifted against each other, which allows for more space in the catalytic site. The loops responsible for the tightening of the active site in the WT enzyme are stabilized by the interaction between Tyr250 and Glu102. Through the disruption of this interaction, variant Y250S induces structural rearrangements, which enlarge the volume of the active site cavity from 153 Å^3^ to 362 Å^3^, resulting in a deeper and more substrate accessible cleft, which contributes to increased hydrolytic activity. All mutations explored in this section are summarized in detail in Table S3.

The enzyme-binding mode was predicted through molecular docking studies with PET tetramer 2-HE(MHET)_4_, MHET, and BHET on both structures. BHET and MHET were predicted to bind in a groove adjacent to the catalytic site, stabilized by interactions with Ser256, Ser248, Asp106, and Ser104. This binding position is not optimal for catalysis, suggesting a necessary conformational change in the active site for interaction between catalytic serine residue and substrate molecules. Furthermore, no valid binding mode for 2-HE(MHET)_4_ was found, due to the narrowness of the active site cleft. Molecular docking with engineered variant Y250S revealed a more favorable binding mode for MHET and BHET, at an appropriate distance for Ser171 attack. Yet again, no valid binding mode for 2-HE(MHET)_4_ was obtained.

#### 2.3.4. Proposed Mechanism

Inspired by their structural and computational studies, Bollinger et al. [97] proposed a mechanism for *Pa*PETase hydrolysis of PET. The proposal suggests a three-moiety substrate-binding mode—one unit would bind the catalytic site adjacent groove, and the other two would bridge the distance to the catalytic site, where the third unit would bind at optimal distance for serine mediated cleavage. The substrate-length dependent catalytic mechanism is similar to the *nick and digestion* mechanism suggested for *Is*PETase.

#### 2.3.5. Future Perspectives

The similarity to *Is*PETase and increased activity of *Pa*PETaseY250S variant is a strong argument in favor of the proposed binding mode, yet further validation through experimental and computational studies is required. Furthermore, given *Pa*PETase similarity to *Is*PETase, reproducing the successful mutations tested on this enzyme could be a promising strategy for enhancing *Pa*PETase activity, complemented with computational strategies such as molecular dynamics simulations. However, this mechanistic proposal should be strengthened by experimental evidence on the binding mode (e.g., three-dimensional structures of complexed *Pa*PETase), and computational studies on the active site conformational flexibility through molecular dynamics, in addition to quantum mechanics studies using, for example, a hybrid QM/MM methodologic approach [102,103,104].

### 2.4. LC-Cutinase (LCC)

#### 2.4.1. Discovery

LCC, a cutinase homologue, was first isolated in 2011 from a leaf-branch compost through a metagenomic approach [58]. Even though the source organism for this enzyme remains to be identified, it is presumed to be thermophilic bacteria. This assumption results from the high sequence identity between LCC and bacterial cutinases (in comparison to fungal cutinases), and the temperature of the compost from which LCC was firstly isolated (67 °C) [105]. LCC is a secretory protein with high sequence identity to lipases and cutinases. Specifically, the highest amino acid sequence identity identified is to *Thermobifida fusca* (57.4%) [58]. The enzyme was found to have hydrolytic activity against various fatty acid monoesters and is efficient in degrading PET and depolymerizing poly(ε-caprolactone) (PCL) [58,106].

#### 2.4.2. Structure

LCC is composed of 258 amino acid residues (amounting to a molecular mass of 28 kDa), plus a 34-residue signal peptide at the N-terminus [58,105]. The overall LCC structural fold belongs to the α/β-hydrolase superfamily and is made up of nine-stranded β-sheet and eight α-helices [105]. The enzyme was determined to be a monomer in the absence of substrates [105]. The surface of LCC has been described as highly charged [107].

**Figure 13:**
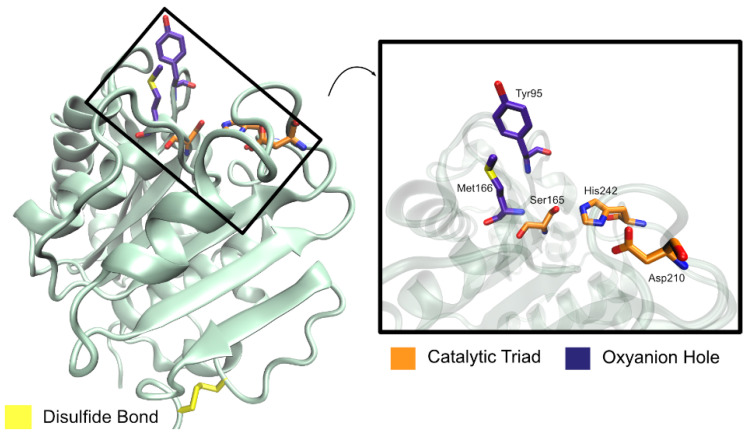
Schematic representation of LCC catalytic triad, oxyanion hole, and disulfide bond amino acid residues, and relative position in overall protein structure.

In the active site, three residues were found to form a catalytic triad (Ser165, Asp210, His242) and two residues constitute an oxyanion hole (Met166 and Tyr95) [58], as represented in Figure 13. The catalytic serine residue is found in a nucleophilic elbow, a sharp turn between strand β5 and helix α5 [105], within a typical GxSxG (GHSMG in LCC) motif [58,105]. Comparisons with *Ta*Cut1, whose binding mode is known, resulted in the prediction that a hydrophobic patch made up of residues Tyr95, Thr96, Ala97, Phe125, Tyr127, Met166, Trp190, Thr211, Val212, and Phe243 constitutes a protruding long groove from the catalytic pocket, accommodating the binding of long-chain substrates [105]. Even so, a specific binding mode remains to be fully understood.

A disulfide bond (Cys275–Cys292) is found to anchor the LCC C-terminus to a strand β9–helix α8 loop, similar to other PET hydrolytic enzymes. This bridge is typically responsible for a higher thermal stability of the enzymes since it exists in thermophilic bacterial cutinases, but not in fungal cutinases [105].

The currently known three-dimensional structures of LCC are summarized on Table 4. All structures exist in apo-form, so no crystallographic data of complexed enzymes are presently available. The first structure was solved at the time of enzyme identification with a resolution of 1.5 Å in 2012 by Sulaiman et al. [58], and allowed authors to confirm the structured features predicted from its sequence and similarity to other enzymes. The remaining available structures, deposited in 2019 by Tournier et al. [106], are engineered versions of LCC.

#### 2.4.3. Activity

LCC activity was first measured against *p*NP-butyrate by Sulaiman et al. [58], to determine optimal hydrological pH and temperature. The enzyme exhibited highest activity at pH 8.0 (with ~70% of maximal activity at pH 7.0 and 9.5) and temperature of 50 °C (with 70% of maximal activity at 30 and 70 °C). Measured activity was not changed in the presence of CaCl_2_ or EDTA. To analyze substrate specificity, several *p*NP monoesters of fatty acids with different lengths acyl chains of 2 to 12 were used as substrates. The enzyme showed preference towards *p*NP-butyrate (C4) and almost equal efficiency with *p*NP-caprylate (C8) and *p*NP-hexanoate (C6). Acyl chain lengths above 12 resulted in much lower rates—enzymatic activity decreases as chain lengths of substrate increases. The enzyme was found to degrade cutin at a similar rate to *T. fusca*.

LCC ability to degrade PCL and PET was also tested by Sulaiman et al. [58]. Specific enzymatic activity against PCL was determined to be 300 mg/h/mg enzyme at pH 8.0 and 50 °C. Regarding PET degradability, the enzyme degraded 1.45 mg of PET film after 24 h incubation, resulting in a specific activity of 12 mg/h/mg enzyme at pH 8.0 and 50 °C. TPA was identified as the major degradation product, with residual MHET also registered. No BHET monomers were detected. This means that LCC completely hydrolyses PET to TPA and EG. This study reported LCC activity to be 230–970-fold higher than that of other cutinases with PET hydrolyzing activity.

As mentioned, LCC showed highest activity at 50 °C. However, the enzyme remains stable up to 75 °C. Sulaiman et al. [105] concluded that activity decreases as temperature increases above 50 °C, even before enzymatic denaturation. To further investigate this, binding affinity constants (K_M_) and turnover numbers (k_cat_) were determined at 30, 50, and 70 °C. K_M_ values were similar, independently of the temperature (0.21–0.24 mM), while k_cat_ numbers differ with the change in temperature. This means that a lowering in activity at 60 and 70 °C is not due to a decrease in binding affinity but to a decrease in the turnover number. Using *p*NP-butyrate as substrate, K_M_ and k_cat_ of LCC are 0.21 mM and 343 s^−1^ at 50 °C, considerably higher than known values for other cutinases. Unlike *p*NP-butyrate, PET is a long-chain substrate that interacts with various binding site residues, differing in this regard from short chain monomers. Activity assays against PET film showed that activity increased at 70 °C—optimal enzymatic activity is higher at higher temperatures. This effect is likely due to changes in crystallinity PET suffers at higher temperatures and stabilization of the active by the long-chain substrate.

The role of catalytic serine residue Ser165 was confirmed by mutagenesis to alanine (S165A), leading to an almost total loss of activity [58,106].

A double mutant disrupting the disulfide bridge (C275A/C292A) was produced by Sulaiman et al. [105] to investigate the predicted stabilizing role of this structural attribute. Spectroscopy studies showed the overall fold of LCC was not affected by this mutation. Measurement of denaturation curves showed a destabilization of approximately 15 °C when compared to WT enzyme, enforcing the essential role of the disulfide bridge in thermal stability. LCC(C275A/C292A) measured activity at 30 °C was comparable to that of WT LCC at 50 °C, indicating that active site stability is affected by the overall enzymatic fold stability.

Tournier et al. [106] compared LCC activity against commercially available amorphous PET with several know PET hydrolyzing enzymes (*Tf*HCut and BTA-2, *Fs*Cut and *Is*PETase). LCC outperformed all tested enzymes in PET degradation, proven to be at least 33 times more efficient with an optimal activity temperature of 65 °C. Furthermore, high thermal stability was confirmed by determination of T_m_ at 84.7 °C. Even so, once again activity decreased as temperature increased, despite high thermal stability, motivating the authors to attempt to increase enzymatic stability and activity simultaneously. Molecular docking and molecular dynamics calculations were performed to predict binding mode of a 2-HE(MHET)_3_ chain. Predicted binding site was consistent with the conserved protruding hydrophobic groove previously described, and inspired 11 site-specific mutations, with production of 209 novel variants. Of these variants, F243I and F243W resulted in improved activity, while T96M, Y127G, N246D, and N246M showed similar activity but higher melting temperature values. In an attempt to further increase thermal activity, an additional disulfide bond (D238C/S283C) was introduced, leading to a T_m_ of 94.5 °C (increased by 9.8 °C) with only 28% loss of activity. Two variants combining the disulfide bridge and the activity increasing mutations were engineered: ICC (F243I/D238C/S283C) and WCC (F243W/D238C/S283C). ICC and WCC presented similar activity to WT enzyme but higher melting temperature values. Finally, eight new variants combining ICC and WCC with previously described T96M, Y127G, N246D, and N246M single-point mutations were generated. Of these, four variants (ICCG, ICCM, WCCG, and WCCM) resulted in similar or higher activity than WT-LCC and improved T_m_ up to 13.4 °C. Exact measurements for these variants and all known LCC mutated enzymes are summarized in Table S4. A three-dimensional structure of variant ICCG was solved at 1.14 Å (PDB: 6THT). Molecular dynamics and MM/GBSA calculations with this structure and 2-HE(MHET)_3_ showed increased affinity and facilitated productive catalytic binding of substrate, further confirming the efficiency of this variant.

Shirke et al. [107] attempted glycosylation to avoid LCC aggregation, under the rationale that reduced aggregation would result in higher kinetic stability. LCC presented aggregation tendencies in its native state resulting from ionic interactions. Glycosylation (covalent binding of an oligosaccharide to protein) may stabilize enzymatic conformation and impose steric constraints inhibiting protein-protein interactions, reducing aggregation. Glycosylated LCC (LCC-G) showed higher thermal stability and increased activity against PET at higher temperatures when compared with non-glycosylated protein. Furthermore, unlike what had been observed for WT enzyme, with LCC-G activity did not decrease with increasing enzymatic concentration, but remained constant once a given activity maximum was attained. LCC-G T_m_ is reportedly 12 °C higher than non-glycosylated LCC.

Yan et al. [108] developed a thermophilic whole-cell biocatalyst with high LCC expression to efficiently degrade PET film. Whole-cell biocatalysis allows for simultaneous enzyme production and hydrolysis in a single step—meaning, the system can produce functional LCC and degrade substrates simultaneously with consistent reaction conditions. *Clostridium thermocellum* (thermophilic anaerobe bacterium with an optimal growth temperature of 60 °C) was used to generate a whole-cell biocatalyst with high secretory expression of LCC at high temperatures. PET hydrolytic activity was registered. Over 60% weight loss of an amorphous PET sample was observed over a 14-day incubation period, resulting in a degradation rate higher than 2.2 mg/day, which is higher than previous whole-cell PET hydrolyzing systems reported. This strategy is frequently simpler, less expensive, and more efficient than biodegradation with purified free enzymes, and this study is highly promising for the future of biocatalysts in plastic biodegradation.

#### 2.4.4. Future Perspectives

Although LCC was first isolated in 2012, several questions remain to be answered. The lack of a protein-ligand three-dimensional structure and accurate binding mode characterization hinders the suggestion of a solid catalytic mechanism. Even so, in recent times this enzyme has received high attention from the scientific community, given the evidence of its high-level activity and thermal stability when compared with other PET hydrolases. The high performance of the few engineered versions developed is promising, and it is to be expected that further mutagenic and activity studies will be successful in enhancing enzymatic activity. Furthermore, since LCC is stable at much higher temperatures than, for example, *Is*PETase, potential for activity above PET’s glass temperature is highly attractive.

### 2.5. Thermomonospora fusca Hydrolase (TfHCut) and Thermomonospora fusca BTA Hydrolase 2 (BTA-2)

#### 2.5.1. Discovery

*Tf*HCut, *Thermomonospora fusca* hydrolase, also referred to as TfH, BTA-hydrolase 1 (BTA-1) [109] or Tfu_0883 [110] is a type I PET degrading cutinase-like enzyme. The ability of *T. fusca*, a thermophilic filamentous soil bacterium [111], to degrade polyester-like substrates, was first identified by Kleeberg et al. [109] in 1998 with BTA, a copolyester of 1,4-butanediol, TPA, and adipic acid.

The initial ability to degrade BTA earned *Tf*HCut the alternative name of BTA-1 [112]. Another enzyme expressed by *T. fusca* also showed BTA and PET degradation activity and was therefore termed BTA Hydrolase-2 (BTA-2). Even though this enzyme has received considerably less attention than BTA-1, they share 92% amino acid identity and the genes that express these proteins are nearly identical [112].

*Tf*HCut was later identified, purified, expressed, and characterized as a thermophilic hydrolase with ability to degrade aliphatic-aromatic copolyesters [113]. The different names this enzyme is known as are due to the uncertainty in classifying it as a lipase or cutinase. When Chen et al. [110] identified specific cutinase-like activity, they renamed *Tf*HCut as *Tfu_0883*, and to this day both designations, as well as BTA-1, are used.

Specific PET degrading activity was first shown by Müller et al. [51] with two different PET samples—commercial bottle PET and PET-B in pellets. *Tf*HCut ability to hydrolase PET was further analyzed and enhanced by Silva et al. [55] and Then et al. [114].

#### 2.5.2. Structure

*Tf*HCut is composed of 261 amino acids with a total molecular weight of 28 kDa [113]. It is, similar to most PET hydrolases, a serine hydrolase with Ser170, His248 and Asp216 as the catalytic triad [110]. Catalytic Ser is found in a G-H-S-M-G conserved sequence [113]. in a nucleophilic elbow [110]. A homology model built by Chen et al. [110] based on *Se*Lip, a *S. exfoliates* lipase, revealed an oxyanion hole formed by Met171 and Tyr100, and confirmed the α/β-hydrolase general fold up made up of a central β-sheet with α-helices on both sides. These findings were later confirmed by Silva et al. [55] that also produced a homology model with a similar protocol.

Kleeber et al. [113] predicted an exposed and not buried binding site, facilitating the attack to the cleavable ester bonds, which Silva et al. [55] later confirmed.

The only three-dimensional structure of *Tf*HCut available was recently released by Dong et al. [115] (PDB: 5ZOA). This structure resolved at 1.54 Å contains all the 261 amino acids, suggests the enzyme is a functional monomer, and confirmed the catalytic triad and oxyanion hole residues predicted by homology years earlier. The authors were unable to obtain structures crystalized with BHET and several cutin mimics. Therefore, molecular docking and molecular dynamics calculations were employed to study the active site and binding mode. Molecular Docking with an oligo-polyester (C_24_H_42_O_8_, CAS number 10061-30-0) that mimics cutin revealed that part of the substrate was inserted in the hydrophobic shallow groove and part was exposed to the bulk solvent environment. An ester bond was located near the catalytic triad, in an optimal binding mode for catalysis to occur. Furthermore, the docked ligand formed hydrogen bonds with the oxyanion hole residues. Given the bulky residues located at either side of the binding groove, it was theorized that availability for substrate accommodation was not optimal. Therefore, these and other structural findings revealed by the structure were used as rationale for protein engineering and activity assays, described below.

Visual inspection of the available PDB structure and comparison with similar PET degrading cutinases suggests a disulfide bridge between residues Cys299 and Cys281.

#### 2.5.3. Activity

Kleeberg et al. [113] showed that the ability of *Tf*HCut to degrade BTA was enhanced by the addition of pectin, peptone, and tryptic soy broth to the medium, and was unaffected by the addition of polysaccharides. Maximum growth temperature for *T. fusca* was determined at 55 °C, and maximum *Tf*HCut activity was registered at 65 °C and pH 6.0 to 6.5, which is a typical behavior for extracellular enzymes. Activity rapidly decreased at 70 °C, likely due to enzymatic denaturation. Ester bond cleavage ability of *Tf*HCut was demonstrated with several triglycerides of varying chain lengths. This activity was higher for shorter chain polymers, decreasing as length increased. Hydrolytic activity on aliphatic polyesters (PCL, commercial biodegradable plastic Bionelle, and SP3/13 and Bayer Tir 1874) was also demonstrated, but no degradation of PHB was attained. Activity assays and predicted characteristics derived from sequence determination led to the definition of *Tf*HCut as a lipase, since specific cutinase-like activity was not confirmed.

PET hydrolytic activity was analyzed by Müller et al. [51] using two different PET samples—commercial bottle PET and a PET-B sample obtained as a pellet. Rates of depolymerization were similar for both samples over a three-week incubation period. The erosion rate was 8–17 μm week^−1^ per film side. Degradation of PET was performed at 55 °C, and DSC experiments confirmed low crystallinity of the PET samples used at this temperature, facilitating enzyme binding and activity rates. Assays with PHB and PBT were also conducted, with no sample weight loss registered.

Silva et al. [55] engineered two new variants of *Tf*HCut with different active site residues, as a strategy to promote active site enlargement (I218A) and to increase hydrophobicity (Q132A/T101A). PET degrading activity was measured with PET fabric at 60 °C and activity rate was determined by measuring amounts of TPA release, since the enzyme can fully degrade PET to TPA. The engineered variants degraded PET to TPA with a two-fold activity increase when compared with the WT enzyme. The mutants achieved a higher catalytic efficiency and higher levels of protein adsorption than WT *Tf*HCut, probably due to the increased active site binding space and enforced hydrophobic character. Further catalytic assays revealed Q132A/T101A to be the better-performed mutant, with a much higher catalytic rate than WT enzyme.

Chen et al. [110] determined esterase, cutinase, and lipase activity of *Tf*HCut. Esterase activity was measure with *p*NPB as a substrate at pH 8.0. Cutinase activity was determined in similar conditions using apple cutin as a substrate, and for lipase activity measurement, triolein was selected as substrate. Enzymatic thermal stability was explored by repeating *p*NPB activity assays at temperatures ranging from 20 to 60 °C. Through verification of high cutinase-like activity, *Tf*HCut, at the time known mostly as TfH or BTA-1, was renamed *Tfu_0883* and considered a cutinase.

Then et al. [114], inspired by the effect Ca^2+^ had on LCC and *Tf*Cut1 activity, explored the activity of *Tf*HCut and BTA-2 in the presence of Ca^2+^ and Mg^2+^ ions. The PET degrading assays were performed on PET film substrate at pH 8.5 and 50 °C for 48 h on a shaker at 125 rpm. The effect on the cations on PET hydrolase activity was dependent on the reaction temperature. At 55 °C, the activity was similar to the WT enzyme. On the other hand, activity at 65 °C was only possible in the presence of Ca^2+^ or Mg^2+^ ions. *Tf*HCut had a 4.5-fold activity increase at 65 °C in the presence of the ions when compared to the WT enzyme performance at 60 °C. Molecular dynamics simulations to determine probable ion binding sites were performed, and concluded that Ca^2+^ was likely to bind Asp174 and Asp204, via the carboxyl groups, and to Gly205 via the amide hydrogen. Mg^2+^ was suggested to bind to five residues—Glu253, Asp174, Asp246, Glu26, and Thr41. Given the positive effects that these cations have on the activity and thermal stability, and the recently known structure of Ca^2+^ bound *Tf*HCut, further computational studies through molecular dynamics, free energy, and QM/MM calculations could be performed to understand PET-degradation activity.

Recently, Dong et al. [115], inspired by the *Tf*HCut structure solved (PDB: 5ZOA), performed several mutations to increase activity on *p*NPB and tomato cutin. Two sets of bulky residues were mutated to alanine residues. The first set included residues near the catalytic triad that might hinder availability of substrate to the catalytic residues. The second set (contained residues near the substrate-binding site in order to increase accessibility for longer chain substrates. In the assays with *p*NPB, Y100A, T247A, and F249A from the first set and L130A and I253A from the second set resulted in enhanced activity. Regarding the tomato cutin experiments, only L130A and I253A (and the combined mutation L130A/I253A) resulted in activity increase. Therefore, molecular docking experiments with the L130A/I253A variant were performed, revealing that the mutation resulted in better substrate accommodation and increased binding site flexibility. These mutations and their effects on enzymatic performance are summarized in Table S5.

Alisch-Mark et al. [116] showed *Tf*HCut ability to modify PET fibers surface, something essential for biodegradation in the textile industry. The role of the catalytic triad was demonstrated by Chen et al. [110]; the effect of PMFS (mechanism-based serine hydrolase inhibitor) on enzymatic activity was measured, with almost no activity registered. Furthermore, the mutation of catalytic serine to alanine also resulted in total loss of activity, confirming the predicted catalytic activity.

*Tf*HCut is an extracellular acting enzyme, as demonstrated by Su et al. [117], by showing hydrolytic activity of phospholipids. The over-expression of the enzyme in various organisms, to achieve optimal quantity and purity levels, has been achieved in several studies [112,118,119], which is a promising experimental milestone for further enzymatic engineering studies.

#### 2.5.4. Proposed Mechanism

Chen et al. [110], inspired by the predicted homology model, suggested a classical α/β-hydrolase catalytic mechanism performed by the catalytic triad (Ser170, His248, Asp216) stabilized by an oxyanion hole (Met171 and Tyr100) and involving formation of two tetrahedral transition states and an acyl-enzyme intermediate.

#### 2.5.5. Future Perspectives

*Tf*HCut was the first PET hydrolase identified and characterized, and has inspired countless organism and enzyme identification campaigns, leading to the discovery of many PET degrading cutinases. The many designations that the enzyme has acquired through the years hinder a good overview of activity and performance. On the other hand, the multiple available studies mean there is more available information on this enzyme than for many other potential PETase-like enzymes. The recent deposition of a three-dimensional structure coupled with experimental evidence of *Tf*HCut ability to fully degrade PET to TPA are promising factors. A deeper understanding of the PET or PET-like substrate binding mode to *Tf*HCut through molecular docking [120,121,122,123,124] and molecular dynamics simulations [125,126,127,128,129,130] could be used not only to understand the molecular rationale behind the performance of the many variants developed, but also to rationally inspire the design of further mutations.

### 2.6. Saccharomonospora viridis AHK190 Cutinase (SvCut190)

#### 2.6.1. Discovery

*Saccharomonospora viridis* AHK190 Cutinase (*Sv*Cut190, also referred to as Cut190) is a PET degrading-enzyme from *Saccharomonospora viridis* AHK190, first identified for its PETase-like activity by Kawai et al. [56] in 2014. *S. viridis* is a thermophilic bacterium with an optimal temperature for growth of 55 °C [131]. The enzyme contains 304 amino acids residues and is only active in the presence of Ca^2+^ ions.

#### 2.6.2. Structure

*Sv*Cut190 exists as a monomer and adopts an α/β-hydrolase fold, with a central twisted β-sheet of nine β-strands and six α-helices. No lid domain was identified covering the active site. Short helices α1, α4, η1, and η2 have also been identified. A conserved pentapeptide sequence motif and a typical catalytic triad of the lipase family, composed of Ser176, His254, and Asp222, which are located on the loops β5–α5, β7–α6, and β8–α7, respectively, have been observed [132]. Residues Phe106 and Met177 have been shown to define an oxyanion hole. A disulfide bond (Cys287–Cys302) that connects the terminal loop to the α8–β9 loop and three Ca^2+^ binding sites, which are located on the β1–β2, β7–β9 and β6–β7 loops, respectively [133] are also present. After binding to Ca^2+^, the enzyme undergoes large conformational changes, which induces the pocket to open providing an easier access for substrate binding [133]. Oda et al. [134] identified the residues involved in the three Ca^2+^ binding sites. The Ca^2+^ binding site located in the β1–β2 loop (Site 1) has an important role on enzymatic activity, and is made up of Ser76, Ala78, and Phe81. Site 2 is in the β7–β9 loop, contributes to thermostability, and involves the amino acids Glu220, Asp250, and Glu296. Site 3, located in the β6–β7 loop, contains amino acid residues Asp204 and Thr206, and is related to both activity and thermostability.

The first crystallographic structures were determined in apo-form by Miyakawa et al. [132] in 2014, complexed with Ca^2+^ (1.75 and 2.35 Å resolution, PDB: 4WFJ and 4WFK, respectively) and without Ca^2+^ (1.45 Å resolution, PDB: 4WFI). The three structures have a mutation on the position 226, where Ser226 was replaced with proline (S226P). In a later report, the same group determined the crystal structures of *Sv*Cut190S176A/S226P/R228S bound to Ca^2+^ and Zn^2+^ (1.60 and 1.22 Å resolution, PDB: 5ZNO and 5ZRQ, respectively) and complexed with Et-succinate and Et-adipate (1.34 and 1.40 Å resolution, PDB: 5ZRR and 5ZRS, respectively) [133]. When the mutated enzyme is complexed with Et-succinate, conformational changes on β3–α2 and β4–α3 loops (designated as the engaged form) are observed. Trp107, which forms a hydrogen bond with Arg135 in the ligand free form, undergoes induced-fit conformational change and interacts with the ligand ester group.

Other *Sv*Cut190 crystal structures available in the Protein Data Bank are summarized and characterized in Table 5.

#### 2.6.3. Activity

The role of Ser176 as a catalytic triad amino acid residue was confirmed when the its mutation to alanine (S176A) totally abolished the enzymatic activity [133]. Kawai et al. [56] determined the T_m_ of WT *Sv*Cut190 at 55.4 °C.

To date, several engineering efforts have been performed on *Sv*Cut190. As summarized in Table S6, *Ta*Est119 has a conserved proline in position 219 (corresponding to 226 in *Sv*Cut190), and due to the similarities of *Sv*Cut190 to *Ta*Est119, the variant S226P was developed, resulting in an enhancement of T_m_ value by 3.7 °C (59.1 °C) and enzymatic activity towards *p*-nitrophenyl butyrate. The positively charged Arg228 can potentially form a salt bridge with a negatively charged residue and influence the reaction. Therefore, Kawai et al. [56] replaced Arg228 with neutral serine, producing the variant S226P/R228S in the presence of Ca^2+^ (300 mM), which resulted in an increase of thermostability and activity (76.8 °C and 0.896 mg/mL, respectively). Thr262 was mutated to the positively conserved lysine, to improve the salt-bridge formation, which is reported to contribute to thermal stabilization. The resulting triple mutation (S226P/R228S/T262K) also in the presence of Ca^2+^ (300 mM) demonstrated an activity increase but a lower T_m_ (76.2 °C) when compared to the double mutant. F106Y mutation appears to cause a steric hindrance of the additional hydroxy group with the catalytic residues, Ser176 and His254 [56].

Mutations on the residues around the catalytic triad, oxyanion hole and the Ca^2+^ binding sites were built to analyze the effects on the catalytic activity, allowing for a better understanding of the enzyme [137]. In this study, a three-dimensional model was developed based on the first reported structure *Sv*Cut190S226P, which was further mutated to *Sv*Cut190S226P/R228S (k_cat_ = 27 ± 0.2 s^−1^; K_M_= 0.089 ± 0.001 mM) with PBSA as substrate. The residues neighboring the oxyanion hole (Phe106, Thr107, Gln138, and Trp201) were mutated to alanine and several other amino acid residues. Overall, the resulting variants decreased the k_cat_ value, except for Q138D variant (increased k_cat_ to 61 ± 0.3 s^−1^), and increased affinity towards PBSA, except for W201A and Q138D [137]. Senga et al. [135] further mutated *Sv*Cut190S226P/R228S by deleting the C-terminal residues (Lys305/Leu306/Asn307). The variant resulted in a k_cat_ of 100 s^−1^ and a K_M_ of 0.282 mM.

Using the same *Sv*Cut190S226P/R228S 3D model [137], Oda et al. [134] applied in-site mutagenesis to the residues involving the Ca^2+^ binding sites, using PBSA as substrate, as 3D modeling revealed similar behavior between PBSA and PET. When compared to other cutinases, *Sv*Cut190 lacks an amino acid in the β1–β2 loop (Site 1); therefore, a serine was introduced between Phe77 and Ala78 (78Ser). This amino acid addition remarkably enhanced the enzymatic activity (k_cat_ = 151 s^−1^); however, its expression was exceptionally low and the thermostability was slightly decreased. In Site 2, a disulfide bond was introduced (D250C–E296C), which significantly increased T_m_ to 79 °C.

Hantani et al. [138] analyzed the catalytic reaction of *Sv*Cut190S226P/R228S and *Sv*Cut190S226P/R228S/Q138A/D250C–296C towards BHET. Kinetic parameters could not be calculated for the later variant due to low solubility of BHET. The final product of BHET degradation was MHET, with no further conversion to TPA. As for Site 3, many mutations were engineered being N202H the variant with better resulting k_cat_ and thermostability. *Sv*Cut190S226P/R228S/Q138A/D250C–E296C/Q123H/N202H is the most thermostable variant of the enzyme determined to date (T_m_ = 85.7 °C). In the presence of 2.5 mM of Ca^2+^ the variant showed a decrease in the thermostability of the enzyme to 82.6 °C [134].

Kawai et al. [56] and Oda et al. [138] performed studies on PET hydrolysis, where different PET films were utilized as substrates. In both studies, *Sv*Cut190S226P/R228S showed the highest activity between 60 and 65 °C. The first group analyzed the degradation rate of PET-GF and PET-S by *Sv*Cut190S226P/R228S at 63 °C for 3 days. The weight losses measured from the molar ratio of TPA included in PET and TPA produced from PET showed a degradation rate of approximately 10.9 ± 1.5% and 26.2 ± 0.6% for PET-GF and PET-S. The analyses by hybrid ion trap/time-of-flight mass spectrometry coupled with liquid chromatography (LC-IT-TOF-MS) and high-performance liquid chromatography (HPLC) detected TPA as the major peak, and no BHET was detected, presumably due to further hydrolysis to TPA [56]. Later, Oda et al. [134] studied the degradation rate products of PET-GF (6.3% of crystallinity) by *Sv*Cut190226P/R228S and variants with the highest melting points at 65 °C and 70 °C, respectively, for 3 days. *Sv*Cut190226P/R228S showed a degradation rate of 16.0 ± 1.4 and expectedly the variants showed higher degradation rate at 70 °C, being the variant with the highest thermostability (*Sv*Cut190S226P/R228S/Q138A/D250C–E296C/Q123H/N202H) the variant with the highest degradation rate (33.6 ± 3.0), resulting in an increase by over 30%, compared with that of SvCut190 at 63 °C.

#### 2.6.4. Proposed Mechanism

Kawabata et al. [137] proposed a catalytic mechanism through molecular docking of partial structure PET model and PBSA substrate with the variant S226P/R228S. His254 deprotonates Ser176, which interacts with the carbon of a carbonyl group by nucleophilic attack, thus splitting the ester bond and forming an acyl-enzyme intermediate. This intermediate is subjected to nucleophilic attack by a water molecule, which is deprotonated by His254, and the resulting proton is transferred to Ser225, creating the conditions for a new catalytic cycle.

#### 2.6.5. Future Perspectives

*Sv*Cut190 has great potential as a PET biocatalyst due to high thermostability of the resulted variants. However, its activity is much too low for its usage in the plastic-degrading industry. Therefore, computational confirmation of the catalytic mechanism should be developed for a better understanding of the reaction.

### 2.7. Thermobifida Genus Cutinase 1 and Cutinase 2 (Cut1 and Cut2)

#### 2.7.1. Discovery

Cutinase 1 (Cut1) and cutinase 2 (Cut2) are two enzymes from the *Thermobifida* genus species, which includes *Thermobifida fusca*, *Thermobifida alba,* and *Thermobifida cellulosilytica*. The enzymes are identical, differing in only 18 amino acid residues. While *T. fusca* and *T. cellulosilytica* are both thermophilic bacteria and can grow at temperatures up to 55 °C and 45 °C, respectively, *T. alba* is a mesophilic bacterium that can only grow at temperatures up to 37 °C. All of these cutinases have the same number of amino acids (262), apart from Cut2 of *Thermobifida fusca* (261). These cutinases were firstly reported as PET biocatalysts by Herrero et al. [139] in 2011.

#### 2.7.2. Structure

Cut1 from *Thermobifida fusca* DSM44342 (*Tf*Cut1 (DSM44342), aka Thf42_Cut1) has high similarity with *Thermobifida fusca* KW3 (*Tf*Cut2), aka ThfKW3_Cut2), differing only in four amino acids [140]. However, structure for *Tf*Cut1 is yet to be determined.

The Cut2 from *Thermobifida fusca* KW3 (*Tf*Cut2) was the first cutinase with a resolved crystallographic structure, determined by Roth et al. [141] in its free state with resolutions of 1.4 Å and 1.55 Å and complexed with the inhibitor phenylmethylsulfonyl fluoride (PMSF) with resolution of 1.44 Å in 2013. All crystallographic structures known for *Tf*Cut2, *Tc*Cut2, and *Tc*Cut1 are summarized in Table 6.

The enzyme adopts a classical α/β-hydrolase fold with a central nine-stranded β-sheet enveloped by 11 α-helices on both sides. The catalytic triad is in a cleft on the surface of the enzyme and is comprised of Ser130, His208, and Asp176. The oxyanion hole is formed by Met131 and Tyr60. The residues Ala55 to Ala65, Leu175 to Pro180 and His208 to Pro211 form the active site [141]. Trp155 and Phe209 interact with the aromatic rings of the dimer PET model, 2PET.

A highly flexible region is located around the amino acids Arg245 to Gly247 and the disulfide bond Cys241–Cys259 is located near this region [141]. The relatively high melting point (71.2 ± 0.2 °C) may be explained by the presence of this disulfide bond and the optimized hydrogen bond network.

The crystallographic structures of Cut1 and Cut2 from *Thermobifida cellulosilytica* (*Tc*Cut1 and *Tc*Cut2, aka Thc_Cut1 and Thc_Cut2, respectively) in their free state were later determined by Ribitsch et al. [142], with resolutions of 1.5 Å for *Tc*Cut1 (PDB: 5LUI) and 1.45, 2.2, and 1.9 Å for*Tc*Cut2 (PDB: 5LUK, 5LUJ, and 5LUL, respectively) in 2016, as described in Table 6. Overall, it is identical to the previously reported *Tf*Cut2, apart from the number of α-helices (11 vs. 10) that flanks the β-sheet. The catalytic triad and oxyanion hole of both cutinases are the same, namely Ser131, His209, Asp177, Tyr61, and Met132, respectively.

Cut1 from *Thermobifida alba* (*Ta*Cut1 aka Tha_Cut1) and *Tc*Cut1 have high similarity, differing in only four amino acids in the outside region of the active site [140]. However, no crystallographic structures have been determined so far.

#### 2.7.3. Activity

Both Cut1 and Cut2 from the previously described organisms are able to degrade PET films. The major product released from the PET hydrolysis by *Tc*Cut1 and *Tf*Cut1 (DSM44342) was TPA [139]. However, MHET was the major product released by *Tc*Cut2, revealing a limiting rate on the MHET hydrolysis to TPA [139]. In addition, an inhibitory effect of BHET on PET hydrolysis by *Tc*Cut1 has been reported [143]. Activity of *Tc*Cut1 on semi-crystalline and amorphous PET films (24 and 12% crystallinity, respectively) demonstrated that the reaction is faster towards semi-crystalline PET [143].

*Tf*Cut2 also demonstrated low capability for MHET hydrolyzation to TPA [144,145,146]. Kinetic parameters of the hydrolysis of MHET and BHET substrates were measured by Barth et al. [144]. The low k_cat_ and high K_M_ towards MHET (0.31 ± 0.01 s^−1^ and 7.33 × 10^−3^ ± 3.62 × 10^−4^ mol L^−1^, respectively) confirm the inhibitory effect of MHET. BHET also has inhibitory effects resulting in k_cat_ and K_M_ of 26.76 ± 0.85 s^−1^ and 3.97 × 10^−2^ ± 2.50 × 10^−3^ mol L^−1^, respectively.

Wei et al. [145] demonstrated that when *Tf*Cut2 is complexed with the dimer PET model 2PET, the residues Gly62, Thr63, Ile178, and Ile213 become in close vicinity to the substrate. The studies of enzymatic degradation calculated with PET amorphous films weight loss revealed that the variant G62A had the highest weight loss of amorphous PET films (42.6 ± 2.9%), while the WT resulted in a weight loss of only 15.9 ± 1.8%. The variant I213S resulted in the highest thermal stability [145].

The substitution of Asp174, Asp204, and Glu253 with arginine confirmed their roles as residues related to the Ca^2+^ binding site in *Tf*Cut2 [114]. The resulting variants (D204A and E253A) increased T_m_ values from 71.2 ± 0.2 °C to 85.2 ± 0.5 °C and 86.2 ± 0.8 °C. Substitution of the Ca^2+^ binding site residues of *Tf*Cut2 to replicate a disulfide bond was studied by Then et al. [147]. The resulting variant (D204C–E253C) increased the melting point to 92.8 ± 0.3 °C. Further engineered variants (D204C/E253C/D174R and D204C/E253C/D174R) increased the PET films weight loss (25.0 ± 0.8% and 24.2 ± 0.8%, respectively), when compared to WT *Tf*Cut2 (16.3 ± 2.2%). The other hydrolase from the same strain *Tf*Cut1 (KW3) has a T_m_ value of 68.6 °C ± 0.6 °C and its thermostability increases in the presence of Ca^2+^ [114]. These mutations are summarized in Table S7.

**Figure 14:**
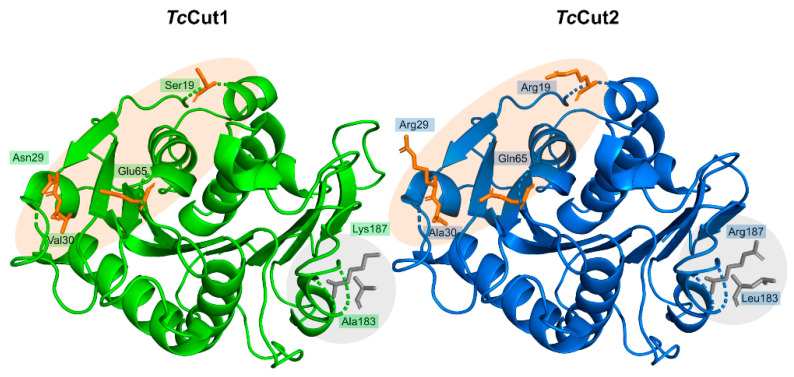
Comparison between different regions in TcCut1 (PDB: 5LUI), in green, and TcCut2 (PDB: 5LUJ), in blue. Region 1 and region 2 are represented in orange and grey, respectively. Amino acid residues are identified by their three-letter code.

*Tc*Cut1 and *Tc*Cut2 differ in only 18 amino acids; however, the hydrolysis of PET films by *Tc*Cut1 was much more effective than that of *Tc*Cut2 [139]. Herrero et al. [139] observed electrostatic potential and hydrophobicity differences in two regions. The first region of *Tc*Cut1 is composed of Ser19, Asn29, Val30, and Glu65, whereas in *Tc*Cut2 it is composed of the positively charged Arg19 and Arg29, Ala30, and Gln65. The second region of *Tc*Cut1 is made up of Ala183 and Lys187, whereas in *Tc*Cut2 the residues are the more hydrophobic Leu183 and Arg187, as evidenced by Figure 14. In a later study, the group applied in-site mutagenesis in regions 1 and 2 of *Tc*Cut2 to resemble *Tc*Cut1 [148]. The double mutant R29N/A30V proved to be the most active variant, with a four-fold activity enhancement for amorphous PET films degradation.

The hydrolysis of a PET model bis(benzoyloxyethyl) terephthalate (3PET) by *Ta*Cut1, which has high similarity to *Tc*Cut1, resulted in a product release of mainly MHET and HEB, and also marginal amounts of TPA and BA [140]. Other methods for enhancement of enzymatic activity of *Tc*Cut1, such as fusion of binding domains, covalent fusion to hydrophobins, and ultrasound have also been reported [149,150,151].

#### 2.7.4. Future Perspectives

The Cut1 and Cut2 of the *Thermobifida* genus have great potential as biocatalysts. A better understanding of the interaction between the PET substrate and the cutinases is needed for PET hydrolysis enhancement. Therefore, studies on the enzymatic mechanism and in-site mutagenesis should be developed.

### 2.8. Fusarium oxysporum Cutinase 5 (FoCut5a)

#### 2.8.1. Discovery

*Fo*Cut5a is a cutinase 5 from *Fusarium oxysporum,* an ascomycete fungus found mainly in soil [152]. FoCut5a was firstly expressed and characterized in 2015 by Dimarogona et al. [153], which confirmed *Fo*Cut5a ability to degrade PET into MHET.

#### 2.8.2. Structure

**Figure 15:**
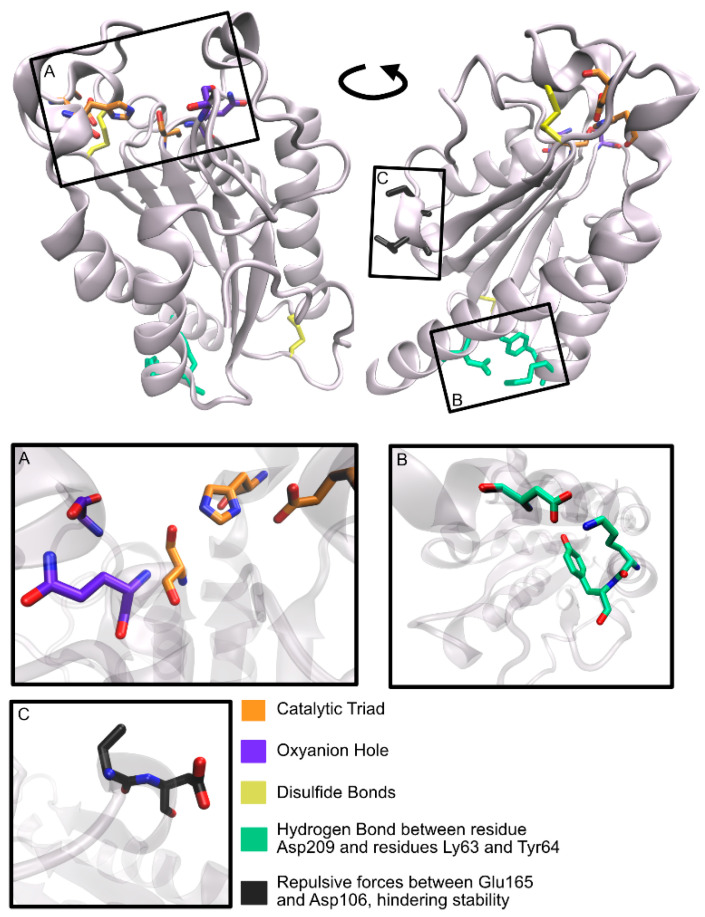
Schematic representation of main structural aspects of FoCut5a. (**A**) Detailed view of the catalytic triad and oxyanion hole residues. (**B**) Schematic representation of the hydrogen bond between residues Lys63 and Asp209, suspected to confer extra thermal stability to the enzyme. (**C**) View of the two repulsing amino acid residues Glu165 and Asp106, thought to hinder enzyme stability.

The enzyme adopts the typical α/β-hydrolase fold with a central β-sheet of 5 parallel β-strands and 11 α-helices, and has a molecular weight of 23 kDa and 230 amino acids [153]. The catalytic triad is made up of Ser121, His189, and Asp176 (Figure 15A). The catalytic serine is located in the nucleophilic elbow, between strand β5 and helix α5. References on the residues comprising the oxyanion hole are yet to be reported [153,154], but an analysis of its X-ray structure suggests these residues are Ser43 and Gln123 (Figure 15A).

Two disulfide bonds are present in the enzyme. The first disulfide bond (Cys32–Cys110) connects the loop α1–β1 to loop α4–β3 and the second (Cys172–Cys179) connects the loop β5–α8 to helix α8, where the catalytic triad residue Asp176 is located [153,154]. The polar residues Lys63 and Tyr64, located at the end of helix α2, form a hydrogen bond with Asp209, which is in the helix α11 (Figure 15B). The interaction between the two is thought to increase the overall thermal stability of the enzyme. Furthermore, the helices α10 and α11 are bridged by the hydrogen bond between the amino acid Gly193 and the backbone oxygen of Leu190. Due to repulsive forces between the Glu165 and Asp166, the stability of the enzyme can be compromised [153] (Figure 15C).

So far, only one crystallographic structure of the enzyme has been reported, having been determined by Dimarogona et al. [153] in its free state with a resolution of 1.9 Å (PDB: 5AJH), in 2015.

#### 2.8.3. Activity

The optimal parameters for *Fo*Cut5a activity are 40 °C, pH 8 and 1.92 mg enzyme loading per gram of PET fabric [153,154,155]. Nikolaivits et al. [154] studied the influence of different *Fo*Cut5a expression hosts on the formation of the disulfide bond and therefore the enzymatic thermostability. The expression of the cutinase gene in the oxidative cytoplasm of Origami 2 competent cells resulted in the highest thermostability, with a melting point of 44.9 ± 0.5 °C.

This cutinase has been shown to hydrolyze the *p*NP esters *p*NPA, *p*NPB, and *p*NPL where the hydrolysis of *p*NPB resulted in the highest catalytic activity and affinity (k_cat_= 111.9 ± 10 s^−1^ and K_M_= 0.7 ± 0.2 mM) [153].

PET hydrolytic activity was confirmed when model 3PET was used as a substrate [153]. *Fo*Cut5a was able to hydrolyze the model substrate, being the products released TPA, BHET and benzoic acid (BA) (0.19, 0.20 and 1.09 mM, respectively). The enzyme was confirmed to further hydrolyze BHET to MHET, when BHET was used solely as the substrate, resulting in a 46% abundance of MHET. The cutinase successfully depolymerized PET fabrics, resulting in a release of 26 μΜ TPA and derivatives [153]. The application of in-site mutagenesis studies is yet to be reported.

#### 2.8.4. Future Perspectives

*Fo*Cut5a is a promising enzyme for PET hydrolysis application, albeit, determination of the X-ray structure complexed with PET substrate should be attempted to enable a better understanding of the key residues involved in the reaction and the catalytic mechanism. In-site mutagenesis could also be applied for enhancement of both activity and thermostability.

### 2.9. Humicola insolens Cutinase (HiCut)

#### 2.9.1. Discovery

*Hi*Cut, aka HiC, is a cutinase produced by *Humicola insolens*, a thermophilic fungus capable of growing at temperatures up to 58 °C [156], first reported in 1964. *Hi*Cut ability to degrade PET substrates was first reported by Ronkvist et al. [157] in 2009. Castro et al. [158] determined that *Hi*Cut has the highest activity towards PET bottle when compared to other 15 biocatalysts, being the amount of released products 29 times higher than the second-best enzyme, *Ro*Lip (commonly known has RoL).

#### 2.9.2. Structure

The enzyme presents an α/β-fold with a central five-stranded β-sheet, which is surrounded by six α-helices on both sides. The enzyme has a molecular weight of 32 kDa and 194 amino acids. The central β-sheet is well conserved in the lipase-cutinase family [159]. The conserved catalytic triad is composed of Ser105, His173, and Asp160 [159]. In the hydrophobic loops adjacent to the active site, three amino acid residues (Leu66, Leu167, and Ile169) may be restricting the entrance of larger substrate [160].

According to UniProtKB [161], *Hi*Cut also exhibits two disulfide bonds (Cys17–Cys94 and Cys156–Cys163). References on the residues comprising the oxyanion hole are yet to be reported, but upon visual inspection of the X-ray structure, residues Gln106 and Ser28 likely make up this motif. The two structures available for *Hi*Cut are described in Table 7. The enzyme in its free form (resolution of 3 Å, PDB: 4OYY) and in complex with mono-ethyl phosphate (resolution of 2.05 Å, PDB: 4OYL), determined by Kold et al. [159], in 2014.

#### 2.9.3. Activity

The enzyme has a relatively high thermostability, showing maximum initial activity at 80 °C and pH 8.5 and becomes totally inactive at 90 °C, presumably due to thermal-induced denaturation [157]. However, Hunsen et al. [162] and Fabbri et al. [163] suggest an optimal temperature of 65–70 °C. High selectivity of *Hi*Cut’s towards long-chain alcohols and acids was revealed, with conversion rates up to 90% when using a 12-chain alcohol, 60 to 70% when using an 8 carbon-chain acid and only 30% when using small acid chains [163,164].

Ronkvist et al. [157] determined that *Hi*Cut fully catalyses low crystallinity PET (7% crystallinity) to TPA at 70 °C, resulting in a 97 ± 3% weight loss in 96 h. However, Castro et al. [158] and Carniel et al. [165] determined a limiting rate on the MHET hydrolysis to TPA when PET and its intermediate BHET were used as substrates. A combination of *Hi*Cut with an enzyme that can properly hydrolyze MHET into TPA (*Ca*LipB) was therefore implemented, resulting in a 7.7-fold increase of TPA concentration [165]. Expectedly, *Hi*Cut reveals a decrease in activity when exposed to higher crystallinity PET films, as determined by Ronkvist et al. [157], where *Hi*Cut activity decreased 10-fold when exposed to PET film with 35% crystallinity compared to the PET films with 7% crystallinity.

While studying the enzymatic hydrolysis of two phthalic acid based polyester coatings (PTa and PTb) by *Hi*Cut and *Tl*Lip, Greimel et al. [166] determined that only the cutinase from *Humicola insolens* was able to hydrolyze the substrates. The unexpected inactivity by *Tl*Lip indicates that the ester bonds between TPA and EG are more accessible to enzymatic hydrolysis than ester bonds between phthalic acid and trimethylolpropane (like in PTa and PTb).

Quartinello et al. [167] developed a two-step system, involving depolymerization and enzymatic hydrolysis. The first step is the depolymerization of the virgin PET fiber, where the optimal conditions were set to be 250 °C and 39 bar. The process completely reduced the substrate to powder consisted of 85% TPA and small PET oligomers. The second step is the introduction of a catalytic enzyme (*Hi*Cut) to further hydrolyze the PET oligomers, which resulted in a total of 97% TPA production.

In addition to temperature and pH, other conditions were shown to increase the hydrolysis product concentrations. Carniel et al. [168] studied the hydrolysis reaction of post-consumer PET by *Hi*Cut and determined that the unbuffered reaction (alkaline water pH 8.95) with NaOH 0.5 M to control the reaction pH showed a 2.39-fold improvement in the released monomers when compared to the Tris-HCl-buffered (pH 8.95). The temperature was constant for both reactions (50 °C). Eugenio et al. [169] determined that the best fitting enzymatic concentration for optimal reaction rate is about 1.68 mg_protrein_ mL^−1^.

Many other methodologies that lead to increased enzymatic activity are reported, including DNA immobilization [170] and optimization of process variables [171]. No studies regarding enzymatic activity and thermostability enhancement towards PET substrate with application of in-site mutagenesis are yet available. However, variants of the enzyme are reported to improve the enzymatic activity of cellulose acetate deacetylation and polyacrylates and poly(vinyl acetate) hydrolase [172,173].

#### 2.9.4. Future Perspectives

Although *Hi*Cut has promising results for PET degradation, due to higher thermostability and activity when compared to other enzymes, it is still far away from being usable in a large scale. Even though no mechanistic proposals for *Hi*Cut degradation of PET-like substrates is available, the existence of a three-dimensional structure complexed with a ligand provides an important starting point for molecular docking and molecular dynamics simulation studies. Studies on the key residues involved in the reaction should be developed for a better understanding of the catalytic mechanism. To increase the enzymatic activity, studies with in-site mutagenesis in these residues should be applied. Kinetic parameters such as k_cat_ and K_M_ should be calculated.

### 2.10. Fusarium solani Cutinase (FsCut)

#### 2.10.1. Discovery

*Fs*Cut or FsC, *Fusarium solani* cutinase, is a fungal cutinase [174] and type I PET hydrolase. This fungus is a widely spread soil pathogen [175]. It was first characterized by Lin et al. [176] in 1980, and several structural and biochemical studies have followed.

#### 2.10.2. Structure

*Fs*Cut is a 230 amino acid residue protein with a molecular weight of 23 kDa. The enzyme is a compact functional monomer with a typical α/β-serine hydrolase overall fold. It is made up of a central β-sheet with five parallel β-strands and five α-helices on either side [174,177,178]. The residues Ser120, His188 and Asp175 make up the catalytic triad [174], stabilized by oxyanion hole residues Gln121 and Ser42 [177]. Catalytic serine is inserted in a Gly-Tyr-Ser-Gln-Gly motif in a β-hairpin [174].

The extended binding site is mostly hydrophobic, but solvent accessible. It is partly covered by two thin “bridges”, formed by amino acid residues Leu81, Val184, Leu182, and Asn84 [174,177,178]. A conserved water molecule bound to Leu179, Ala185, Ile183, and catalytic residue Asp175 has a role in properly orienting side-chains and facilitating catalysis [177]. Binding site amino acids have overall higher B factor than the remaining protein residues, suggesting conformation changes and rearrangements upon binding and catalytic attack [174].

The 46 three-dimensional structures of *Fs*Cut available on PDB are summarized in Table 8. There are currently five apo-form and five complexed WT structures available, and thirty-six engineered variants. The first structure was deposited by Martinez et al. [174] in 1994, at 1.25 Å (PDB: 2CUS). In the same year, a structure complexed with a covalent inhibitor (PDB: 2CUT) revealed the displacement of a conserved water molecule upon binding, with no other structural aspects affected [177]. Longhi et al. [179] produced 31 *Fs*Cut variants with different mutated residues and complexed ligands, and studied the effects of these alterations on the enzymatic structure, having concluded that the binding of a non-hydrolysable substrate analogue did not induce structural rearrangements. The same authors [180] later obtained a complex of *Fs*Cut with a triglyceride analogue similar to the first tetrahedral intermediate (TI) in the reaction pathway (PDB: 1CEX), further confirming the unaffected overall structural and catalytic fold.

#### 2.10.3. Activity

Heumann et al. [183] have demonstrated *Fs*Cut’s ability to hydrolyze BHET, and to modify the surface of synthetic PET fabric with positive yields.

In order to enhance enzymatic activity towards PET, Araújo et al. [184] tested several active site point mutations that aimed to enlarge the binding site, providing a better fit for larger polymer chains. Through computational studies of molecular dynamic simulations and free energy calculations with a substrate mimicking the TI, five point mutations were predicted as favorable for activity and model TI stabilization—L182A, V184A, L189A, L81A, and N84A. Experimental testing of these variants with PET fabric as a substrate for 48 h at pH 7.5 and 37 °C showed L182A, L81A, and V184A resulted in increased activity, while L189A and N84A resulted in activity decreases. Subsequent structural and hydrophobicity studies confirmed that the better performing variants resulted in a less restrained active site, allowing for better accommodation of substrate and higher stabilization of TI, without affecting the hydrophobicity and therefore the grip on PET fabric surface.

Ronkvist et al. [157] studied the effect of PET crystallinity percentage on cutinase activity and compared *Fs*Cut with *Hi*Cut and *Pm*Cut. The substrates were two samples of PET—low crystallinity PET (7%) and a bi-oriented sample of PET with 35% crystallinity. Of the three enzymes tested, *Fs*Cut showed the lowest affinity for PET and the poorer thermal stability, with a maximum activity at 50 °C, far from PET T_g_. *Fs*Cut ability to degrade PET decreased as sample crystallinity increased, as expected. The mild temperature for *Fs*Cut maximum activity combined with its rapid denaturation as temperature increases constitutes a meaningful impairment to its PET degrading action.

Erbel et al. [185] tested *Fs*Cut activity on PET fabrics and 3PET in the presence of Triton X-100 and plasticizer N,N-diethyl-2-phenylacetamide (DEPA). Plasticizers enhance polymer chain mobility by reducing T_g_ and inner chain interaction. Even though Triton-X-100 had no effect on *Fs*Cut ability to modify PET, DEPA resulted in enhanced hydrolysis rates, further confirming that FsC biggest limitation in degrading PET is the polymer’s high T_g_ and low chain mobility at the enzyme’s optimal temperature.

#### 2.10.4. Future Perspectives

*Fs*Cut, similar to many known and described cutinases, is regarded as a promising PET hydrolase. However, thermal stability and low optimal activity is a main issue in the application of this enzyme for efficient PET depolymerization. There have been no mechanistic proposals for the *Fs*Cut degradation mechanism of PET. Since the structure and activity of this enzyme is extensively characterized, several computational studies could be confidently employed to resolve the enzymatic mechanism, particularly QM/MM [102,103,104]. The existence of high-resolution three-dimensional structures with a TI model substrate is a relevant clue for mechanistic studies and could be used to validate a QM/MM study. Finally, strategies to improve thermal stability should be employed.

### 2.11. Candida antarctica Lipase B (CaLipB)

#### 2.11.1. Discovery

*Ca*LipB, CalB, or lipase B from *Candida antarctica*, was first isolated in 1988 [186,187], and has been the target of various studies. *Ca*LipB is a multi-purpose enzyme, with various roles attributed to it, such as resolution of alcohols [188] and amines, desymmetrization of diacetates and diols, synthesis of intermediates for substrate, and pharmaceuticals production [189]. This large number of applications arises because *Ca*LipB presents a high thermal stability, low substrate specificity, high enantioselectivity, and stability in diverse solvents [190].

#### 2.11.2. Structure

*Ca*LipB is made up of 317 amino acid residues and has a molecular weight of 33 kDa. Optimal catalytic pH was determined to be 7. In addition, *Ca*LipB was shown to remain stable in aqueous media from pH 3.5 to 9.5 [191]. The overall structure follows the typical α/β-globular protein fold. The catalytic site contains the typical Ser-His-Asp triad (Ser105, His224, Asp187), stabilized by an oxyanion hole (Thr40 and Gln106). Ser105 is introduced in a TWSQG motif, differing from the typical motif characteristic of PET hydrolyzing enzymes [192]. *Ca*LipB exists as a monomer, mostly composed of β-sheets surrounded by α-helices, similar to the typical α/β-serine hydrolase fold [188,192].

The catalytic triad is located at the carboxy-terminal hedge of the main parallel β-sheet, with Ser105 located in a tight turn between α4 and β4. His224, located in helix α9 with a protruding sidechain into the active site, is at optimal catalytic distance from Asp187, located in a turn after the sixth strand. Ser105 is surrounded by polar residues (Thr40, Asp134, Gln157) that form hydrogen bonds and are fully accessible to solvent interactions [192]. Stauch et al. [191] revealed the existence of a conserved water molecule shared between catalytic His224 and Ser105, while Strzelczyk et al. [189] identified a water bound to catalytic Asp187 and nearby Ser227. The binding pocket is divided into two compartments with different affinities—one that accommodates the acyl moiety and the other that preferably binds the substrate alcohol part [188,189,192]. The alcohol-binding subsite is further divided into a large and a medium pocket [193].

There are currently 25 *Ca*LipB three-dimensional structures in the Protein Data Bank, including apo forms of the enzyme and forms co-crystallized with ligands of relevance. These are summarized in Table 9.

Uppenberg et al. [188,192] solved the first X-ray structures of this enzyme in 1992. These structures revealed that all six cysteine residues in the protein form disulfide bridges: Cys22–Cys64 and Cys216–Cys258 contribute to the overall protein fold, and Cys293–Cys311 stabilizes the enzymatic C-terminal. A possible N-glycosylation site at Asn74 was also identified.

From the resolved structures, amino acid sequence, and similarity to other well categorized lipases, the existence of a lid domain responsible for an open and closed conformations was predicted [192]. However, the proven existence of a lid and a precise characterization of it was only achieved in 2016, when Stauch et al. [191] resolved a two-monomer structure representing both conformations at 0.91 Å (PDB: 5A71). The two monomers, A and B, assume different conformations. Monomer A takes on the classical open conformation, similar to previously reported structures, while monomer B reveals a huge conformational change. Residue range Leu140–Leu147 is an α-helix (α5) in monomer A, but in monomer B, it becomes an unfolded loop, corresponding to a closed conformation. The closing of the lid hinders substrate accessibility to the binding site, preventing catalytic activity.

Regarding the binding mode, Uppenberg et al. [192] obtained *Ca*LipB in complex with Tween 80 (PDB: 1LBT), a monoester prior to hydrolysis, which remained highly exposed to solvent and did not disrupt the overall fold; and a complex with a phosphonate inhibitor (PDB: 1LBS), which revealed disruption of the optimal distances for catalysis. Qian et al. [194] solved a suicide-inhibitor-complexed structure after circular permutation, and Xu et al. [197] resolved a complex structure with a synthesized stereoisomer product in order to understand stereoselectivity of *Ca*LipB.

#### 2.11.3. Activity

**Figure 16:**
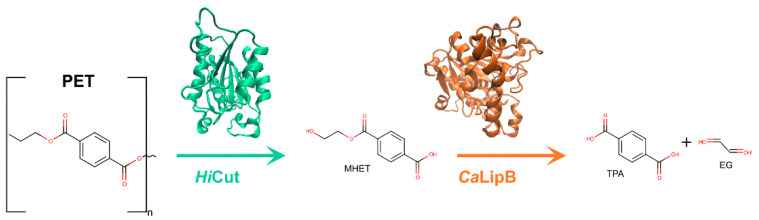
Cumulative action of HiCut and CaLipB to fully degrade PET to TPA and EG.

*Ca*LipB activity towards PET is not consensual [51,199,200]. The most promising report was the evidence found by Carniel et al. [165] that *Ca*LipB has MHETase and BHETase activity, efficiently degrading BHET and MHET to TPA and EG. These studies were conducted synergistically with *Hi*Cut (as represented in Figure 16), which showed high affinity and activity in PET depolymerization. Using BHET as a substrate, Carniel et al. [165] screened 10 commercial enzymes and identified *Ca*LipB and *Hi*Cut as potential biocatalysts. Preliminary screening was conducted at pH 7.0 and 37 °C and revealed that *Ca*LipB led to a fast conversion of BHET to MHET and, consequently, MHET to TPA with higher extent than previously tested lipases. Activity assays with PET bottle after different pre-treatments showed a better performance achieved by *Hi*Cut than *Ca*LipB. After 3 weeks of activity on the PET bottle and pellet, a maximum of 0.4% weight loss, but almost total degradation of MHET and BHET to TPA, was achieved when the reaction environment was high on these substrates. Therefore, further studies to enhance *Ca*LipB activity as a promising MHET and BHET hydrolyzing enzyme are essential to confirm this enzyme as an essential player in the PET bioremediation landscape. To our knowledge, there are no reported studies aiming at enhancing *Ca*LipB hydrolysis of PET, MHET, and BHET.

Due to the diverse applications of *Ca*LipB, countless engineering efforts towards increased activity and higher stability have been reported with various substrates, unrelated to PET or plastic polymers in general [201,202,203,204,205,206,207]. The ability of *Ca*LipB to play an essential role in polyester and bioplastic synthesis has also been the target of interesting activity and catalytic studies [208,209].

Even though these studies are not specific to PET, some may result in valuable suggestions and strategies. Qian et al. [194] redesigned *Ca*LipB by circular permutation, a technique that changes connectivity and sequence order but does not affect overall fold. Circular permutation occurs naturally throughout organism evolution and is frequently used as an artificial engineering technique. Variant CalB-cp283 was generated and its three-dimensional structure solved (PDB: 3ICV). Activity assays showed increased activity and a high-rate enhancement against *p*NP-butyrate, and structural studies suggest CalB-cp283 suffered dimerization. Xie et al. [195] followed the rationale of mutating residues within 10 Å and a high B factor and obtained a double mutant D223G/L278M (PDB: 4K5Q) with higher activity against *p*NP-caprylate and higher stability than the WT protein.

#### 2.11.4. Future Perspectives

The recent discovery that *Ca*LipB has degradation activity towards BHET and MHET and can act synergistically with PET degrading enzymes that are unable to complete degradation (such as *Is*PETase and *Hi*Cut), is a very promising approach. However, so far, no significant additional studies have been conducted. Given the high number of three-dimensional *Ca*LipB structures, many of them recent and with high-resolution values, computational mechanistic studies aimed at understanding *Ca*LipB as probable (yet disputed) role as a PET degrading enzyme could be conducted. Specific understanding of the binding mode of these molecules and mechanistic solving and characterization would allow for the development of engineered variants with enhanced activity. *Ca*LipB has received little attention from the community as a PET degrading enzyme, but its promising experimental behavior justifies further investment.

### 2.12. Thermobifida alba Esterase 1 (TaEst1)

*Ta*Est1 is a 260 amino acid residues cutinase from *Thermobifida alba* identified in 2013 [45,210]. *Ta*Est1 has an increased activity on *p*NPB of two-fold in comparison with to *Ta*Est119 and is able to fully depolymerize 3PET to TPA and hydrolase the surface of PET film [45,210].

Efforts to obtain a three-dimensional structure of *Ta*Est1 have been reported by Kitadokoro et al. [211], but no resolved structure has been deposited on the PDB database to date. Thumarat et al. [210] built a homology model based on a known structure of *Ta*Est119, and identified the catalytic triad as residues Ser169, His247, and Asp215 and the oxyanion hole as made up of Met170 and Tyr99, similar to *Ta*Est119. The two enzymes differ in the residues of a loop near the substrate binding-site, which might explain the higher activity of *Ta*Est1 compared with *Ta*Est119. The overall fold is consistent with the typical α/β-serine hydrolase structure, with a central, nine-stranded parallel β-sheet flanked by eight α-helices on either side.

Kitadokoro et al. [211] determined the T_m_ of *Ta*Est1 to be 61 °C. Activity assays with *p*NPB in the presence and absence of Ca^2+^ cations at pH 6.0 and 50 °C revealed that in the presence of calcium ions T_m_ increased up to 76 °C. Inspired by a conserved valine residue in most cutinases, variant A68V was tested and resulted in higher activity.

Thumarat et al. [210] engineered several *Ta*Est1 variants and tested them for activity on *p*NPB substrate, as described in Table S8. The better performing variant (A68V/T253P) was later tested with 3PET and PET film as substrates. Variants with point mutations and combined modified residues A68V, T253P, and M256K were constructed based on typical cutinase residues absent in *Ta*Est1. Variant A68V resulted in increased activity (similar to what had been observed by Kitadokoro et al. [211], but no increase in T_m._ Therefore, variant A68V/T253P was constructed since proline residues typically stabilize overall enzymatic structure. This double mutant resulted in higher activity and an increased T_m_ of 79 °C in the presence of calcium. Furthermore, *Ta*Est1A68V/T253P conserved 100% of activity at 50–55 °C after 1 h, 90% of activity at 60 °C for 1 h and 70% activity at 65 °C for 30 min. Therefore, this variant showed remarkably improved activity and thermal stability when compared with WT *Ta*Est1. Variants A68V/M259K and A68V/T253P/M259K resulted in similar activity to the A68V/T253P double mutant but lower T_m._

Following these results, Thumarat et al. [210] tested the best performing variant, A68V/T253P double mutant, with 3PET and PET film as substrates in the presence of Ca^2+^ at pH 7.0 and 50 °C for 3 h. After 4 min of incubation, 3PET was depolymerized to TPA and EG almost to completion, confirming *Ta*Est1 PET depolymerization activity. In the assays with PET film, even though no weight-loss was recorded, evidence of hydrolyzation of ester bonds in the film surface with release of hydroxyl and carboxyl groups was observed. Less efficiency in PET film degradation compared with assays with substrate 3PET is likely due to the higher crystallinity of PET, and less availability for enzymatic degradation. Further efforts to increase *Ta*Est1 T_m_ to higher value than the T_g_ of PET are essential to enhance enzymatic activity and applicability in large-scale PET depolymerization.

### 2.13. Thermomyces lanuginosus Lipase (TlLip)

#### 2.13.1. Discovery

*Thermomyces lanuginosus* lipase (*Tl*Lip, aka *TlL*) is produced by a basophilic fungus previously known as *Humicola lanuginosa* discovered by Tsiklinskaya et al. [212] in 1889. TlLip consist of a single chain of 269 amino acids, with a molecular weight of 31 kDa and an isoelectric point of 4.4, being firstly purified in 1972 by Arima et al. [213]. The enzyme was applied in various industrial areas [214,215,216,217], including in the hydrolysis of PET, firstly reported by Brueckner et al. [218] in 2008.

#### 2.13.2. Structure

The enzyme adopts the α/β-hydrolase fold with a central β-sheet containing eight predominantly parallel β-strands, and five α-helices. The catalytic triad is made up of Ser146, His258, and Asp201 and the oxyanion hole by Ser83 and Leu147 [219,220]. Three disulfide bonds (Cys22–Cys268, Cys36–Cys41, and Cys104–Cys107) have also been reported.

The residue Glu87 was suggested to take part in the electrostatic stabilization of the lipase active open-lid conformation and Trp89 was proposed to play an important role in hydrolytic reactions by interacting with the substrate [221,222,223].

Derewenda et al. [220] determined the first crystallographic structure of *Tl*Lip in its free state with a resolution of 1.84 Å (PDB: 1TIB), in 1993. Presently, 25 crystallographic structures have been deposited in the Protein Data Bank. All structures are summarized and characterized in Table 10.

#### 2.13.3. Activity

The optimal activity temperature and pH of *Tl*Lip were reported to be 45 °C and 7, respectively [230]. This enzyme was shown to be stable for 20 h at 60 °C and 1 h at 65 °C, losing its activity at temperatures higher than 70 °C, being the melting temperature around 65 to 70 °C [227,230].

In-site mutagenesis in the amino acid Ser146 (S146A) resulted in an inactive form of the enzyme, confirming its role as part of the catalytic triad [231]. Replacing Glu87 with alanine (E87A) and Trp89 with phenylalanine and glutamate (W89F and W89E, respectively) resulted in lower specificity towards tributyrin, suggesting that these residues play a role in the substrate binding [221,222]. The mutation of Ile186 and Ile255 to cysteine was applied to introduce a new disulfide bond (I186C/I255C). The resulting disulfide bond decreased the thermostability by 5 °C, meaning that the addition of a new disulfide bond destabilizes the secondary structure of *Tl*Lip [227]. These engineering efforts are summarized in Table S9.

*Tl*Lip was able to degrade both amorphous and semi-crystalline PET fibers (5 and 40% crystallinity, respectively) releasing about two times more TPA and MHET from amorphous fibers. However, the amount of product released is much too low when compared with *Thermobifida fusca* cutinase [218]. Interestingly, the enzyme released higher amounts of MHET than TPA, whereas the cutinase released similar amounts of both products. This shows that both enzymes have different reaction mechanisms [218].

The hydrolysis of the PET model substrate 3PET resulted in the release of TPA, BHET, MHET, BA, and HEB [185]. When in presence of the non-ionic detergent Triton X-100, conformational changes involving lid-opening of the lipase occur, resulting in a 7-fold increase of the overall degradation products [185]. When incubated solely with BHET, the lipase did not liberate significant amounts of TPA, showing that *Tl*Lip preference towards the other intermediate product MHET [185].

#### 2.13.4. Future Perspectives

*Tl*Lip is a promising enzyme for application in PET degradation. However, the activity and thermostability of the wild type enzyme are too low for direct usage in the plastic-degrading industry. Determination of its structure complexed with PET substrate or its analogue could provide a better understanding of the key residues involved in the reaction and their conformation, yielding important clues for a rational understanding of its catalytic mechanism. Calculation of the kinetic parameters k_cat_ and K_M_ for the wild type enzyme and different mutants, following in-site mutagenesis would also be of great importance for enhancing the activity and thermostability of this enzyme.

### 2.14. Thermobifida fusca Carboxylesterase (TfCa)

#### 2.14.1. Discovery

*Tf*Ca is a highly hydrophobic carboxylesterase produced by *Thermobifida fusca*, a meso-thermophilic bacterium known for its ability to degrade cellulose. This organism produces many biocatalysts, being a powerful resource for synthetic polymer degradation [112,139,144]. The enzyme’s properties as a PET biocatalyst were firstly described by Oeser et al. [232] in 2010.

#### 2.14.2. Structure

*Tf*Ca is composed of 497 amino acids and exists as a monomer. Identical sequences with other carboxylesterases are also observed [233]. The catalytic triad includes amino acid residues Ser185, His415, and Glu319. The catalytic serine is inserted in a motif containing residues Glu184 and Ala186 [233]. There are currently no X-ray structures available for this enzyme, so no further structural information is known.

#### 2.14.3. Activity

*Tf*Ca presents a low isoelectric point (4.8) and a thermostability between 30 and 50 °C [233]. When incubated with *p*NPB at temperatures above 55 °C, the enzyme completely loses its activity [234]. However, after immobilization of *Tf*Ca on SulfoLink resin (where an oligopeptide of glycine-serine-cysteine was added at the C-terminus), the enzyme exhibited an improvement of thermostability, maintaining 94% of its initial activity at 60 °C [234]. The carboxylesterase is capable of hydrolyzing PET nanoparticles at 50 °C and cyclic PET trimers at an optimum temperature of 60 °C and pH 6 [232,233]. The products released towards cyclic PET trimers are 1,2-ethylene-mono-terephthalate-mono(2-hydroxyethyl terephthalate (EMT) (95%), MHET (3%), and BHET (2%). The enzyme also showed a K_M_ value of 0.5 ± 9% mM for cyclic PET trimers. Belisário–Ferrari et al. [235] analyzed the hydrolysis of 2PET and BHET by *Tf*Ca. The hydrolysis of the PET intermediate resulted in a 3.5-fold higher k_cat_ (0.35 min^−1^) and a 2-fold higher K_M_ (0.085 mL/mg) when compared to 2PET (0.10 min^−1^ and 0.042 mL/mg of k_cat_ and K_M_, respectively). These results revealed a preferential hydrolysis of smaller esters by the carboxylesterase

Barth et al. [234] built a dual enzyme system with LCC and *Tf*Ca, employing the latter for the hydrolysis of MHET to TPA. In fact, the dual system resulted in a 2.4-fold increase of product release (10.42 ± 1.85 mM) when compared with that of *Tf*Cut2 (4.44 ± 0.57 mM). A significant increase of product release was also reported when the enzyme was coupled with *Tf*Cut2 [234]. Mutations on the sequence motif (E184Q, A186M, and E319D) of the carboxylesterase resulted in activity losses up to 88%, confirming their important roles for the catalytic activity [233], as described in Table S10.

#### 2.14.4. Future Perspectives

As little information on *Tf*Ca is currently available, determining the crystallographic structure of the enzyme would provide a deeper understanding of its activity at the molecular level. To reach the plastic-degrading industry standard levels, in-site mutagenesis should be applied for enhancement of thermostability and enzymatic activity.

### 2.15. Lesser-Known Enzymes

In addition to the 16 enzymes described in detail above, over the years, several other enzymes have been reported to exhibit some PET degrading ability. These are portrayed in this section.

#### 2.15.1. *Enterobacter* sp. HYI Esterase B (*Es*EstB)

*Es*EstB, or EstB, is an esterase from *Enterobacter* sp. HYI isolated and characterized by Qiu et al. [236] in 2020. The enzyme was shown to be able to degrade BHET to MHET and TPA. The bacterial strain was isolated from a plastic waste treatment station and identified as an esterase after assays confirming esterase-like activity.

Qiu et al. [236] characterized *Es*EstB as sharing 99.7% sequence similarity with esterase from *Enterobacter hormaechei*. However, no BHET or PET degrading activity has been reported for this enzyme. Sequence-based structure predictions revealed the typical α/β-hydrolase fold, with a catalytic triad composed of residues Ser110, Asp158, and His190.

Activity assays showed maximum activity was attained at an optimal pH of 8.0 and temperature of 40 °C. *Es*EstB degraded 80.8% of BHET in 120 h at a degradation rate of 6.73 mg/L·h to produce MHET and further degrades MHET to TPA. The ability for complete degradation of BHET, frequently used as the PET commercial model, is promising for future applications of *Es*EstB in PET degradation.

#### 2.15.2. HR29 Hydrolase (*Bhr*PETase)

*Bhr*PETase, a hydrolase from thermophilic bacteria *HR29*, was recently overexpressed in *Bacillus* s*ubtilis* by Xi et al. [237] and shown to fully degrade PET. HR29 is involved in the biochemical nitrogen cycle and was identified by Kato et al. [238] in a long-term cultivation of thermophiles. Xi et al. [237] expressed *Bhr*PETase in *B. subtilis* and characterized it. The enzyme shares 94% sequence identity with LCC, differing in 16 amino acid residues only.

*Bhr*PETase is a 275 amino acid residue enzyme with a conserved α/β-hydrolase fold and one disulfide bridge (Cys275–Cys292). The catalytic triad is made up of residues Ser165, His242, and Asp210.

Given the advantages of expressing enzymes in *B. subtilis* due to this bacterium being non-pathogenic, robust, and highly studied, Xi et al. [237] expressed LCC, *Is*PETase, and *Bhr*PETase to compare activity and thermal stability. Enzymatic activity was firstly characterized with a series of monoesters of varying lengths, and then specific PET degrading activity was measured using BHET and low crystallinity PET powder as substrates. Depolymerization of PET substrates yielded TPA as the major product, and BHET and MHET in lower amounts. Similar to other PET degrading enzymes, *Bhr*PETase exhibited higher activity on shorter acyl-chain length substrates, and maximum activity at a pH of 6 to 8. *Bhr*PETase showed remarkable thermal stability by linearly increasing activity from 30 to 90 °C, contrary to LCC that, although highly stable, suffered an activity decrease above 80 °C. Furthermore, *Bhr*PETase T_m_ reached 110 °C, the highest known to PET degrading enzymes, and increased by 6.4 °C in the presence of Ca^2+^ ions. Comparison with LCC suggests Glu208, Asp238, and Ser283 as probable calcium binding residues. *Bhr*PETase proved highly active on PET powder complete degradation to BHET, MHET, and TPA, with the highest conversion occurring at 70 °C.

Although there is no three-dimensional structure available for *Bhr*PETase, the high similarity with LCC justified the design of an LCC-based homology model. The differences found in the sequence might explain the higher thermal stability of *Bhr*PETase regarding LCC. Xi et al. [237] proposed that Ser175 and Gln202 (which occupy the positions of Ala175 and Val202 in LCC) establish hydrogen bonds with nearby residues, replacing the hydrophobic interactions in LCC. Furthermore, two proline residues in *Bhr*PETase (Pro199 and Pro248) occupy the positions of a serine and an asparagine in a loop region in LCC. It has been argued [237] that these proline residues might result in higher rigidity of the loop contributing to thermal stability of the overall structure.

Overall, *Bhr*PETase is a novel enzyme with high PET degrading activity, thermal stability, and a T_m_ higher than the T_g_ of PET, features that make it a very promising enzyme for further studies on the biodegradation of PET.

#### 2.15.3. *Bacillus subtilis* Lipase (*Bs*EstB)

*Bs*EstB, a *Bacillus subtilis* lipase, was firstly isolated by Eggert et al. [239] in 2000. This enzyme was demonstrated to have PETase-like activity by Ribitsch et al. [53] in 2011. *Bs*EstB is an intracellular p-nitrobenzylesterase with a molecular weight of 55.2 kDa and an isoelectric point of 4.9. It was identified as belonging to the α/β-hydrolase family, similar to most PET hydrolytic enzymes, and the catalytic triad is made up of residues Ser189, His399, and Glu310. The enzyme shares a 99% similarity with typical *B. subtilis* and carboxylesterase type B [53].

Activity for PET degradation was firstly identified in a large screening of 250 enzymes with 3PET on agar plates. Subsequently to identification, isolation and expression, *Bs*EstB was submitted to activity assays with PET film and BHET [53]. The enzyme is able to degrade PET to completion, as the major products of hydrolysis are TPA and benzoic acid. MHET was identified as a minor product and a rapidly degraded intermediate. Maximum activity was registered at 40 °C and pH 7 [53] The ability to completely depolymerize PET to its constituent monomers while being an intracellular enzyme is uncommon, and suggests this lipase is an ideal target for further PET degrading assays and studies.

#### 2.15.4. *Streptomyces scabies* Sub1 (*Sc*Sub1)

*Sc*Sub1, commonly known as Sub1, is a cutinase-like enzyme from *Streptomyces scabies*, a gram-positive plant pathogen bacterium [240]. The protein was first discovered per identification of the gene *sub1*, which showed specific expression in the presence of cutin and suberin [241], and was recently characterized and subjected to specific activity assays [240]. Sequence similarity to fungal cutinases led to the classification of *Sc*Sub1 as a cutinase-like enzyme.

The structure for *Sc*Sub1 [240] was predicted using the ESyPred3D [242] server with a cutinase from *Aspergillus oryzae* (PDB: 3GBS) as a template. The resulting structure suggests a canonical α/β-cutinase-like fold, made up of a five parallel strands central β-sheet surrounded by ten α-helices. The same prediction suggests the existence of a catalytic triad composed of Ser114, His195, and Asp182, and two disulfide bonds, Cys31–Cys103 and Cys178–Cys185. The total molecular weight of *Sc*Sub1 is estimated to be 25 kDa, which includes mature *Sc*Sub1 with 18.7 kDa and a His-tag of 4.9 kDa.

PET degradation by *Sc*Sub1 was assessed by measuring the amount of TPA produced, at pH 7.5 and 37 °C for 20 days. The substrate used was commercially available PET granules, and TPA production remained constant during the assay time, resulting in a proportional relationship between incubation time and TPA release. Furthermore, the stability of *Sc*Sub1 at 50 °C was evaluated, and even though *Sc*Sub1 kept active, TPA production diminished with incubation time, suggesting progressive loss of stability and catalytic activity.

Interestingly, *Sc*Sub1 showed esterase activity by successfully degrading different substrates such as *p*-nitrophenyl butyrate, *p*-nitrophenyl octanoate, *p*-nitrophenyl decanoate, and *p*-nitrophenyl dodecanoate. Activity of *Sc*Sub1 on cutin and suberin was also confirmed and showed to be stable at room temperature over a 20-day period.

#### 2.15.5. *Pseudomonas mendocina* Cutinase (*Pm*Cut)

*Pm*Cut, aka PmC, is a cutinase from *Pseudomonas mendocina* first described as a PET degrading enzyme by Kellis et al. [243] in a patent proposition for enzymatic modification of polyesters in 2002. Inspired by this finding, Ronkvist et al. [157] included *Pm*Cut in a comparative study with *Fs*Cut and *Hi*Cut towards the understanding of the effect of PET crystallinity percentage on activity. Using two PET samples as substrate, the authors concluded that *Pm*Cut had the highest affinity for low crystallinity PET, having resulted in 5% PET film weight loss after 96 h at 50 °C. Degradation rate of *Pm*Cut was lower than that of *Hi*Cut but higher than *Fs*Cut, presumably due to differences in thermal stability and optimal activity temperature. From the available data, it is to be expected that *Pm*Cut has a high potential as a PET degrading cutinase. However, further biochemical, and structural studies are essential to confirm enzymatic structure, sequence, and specific role in PET degradation.

#### 2.15.6. PET1–PET13

Danso et al. [244] identified 504 possible PET hydrolytic enzyme candidates through an automatic search algorithm. Of these, 13 enzymes were selected and named PET1 through PET13. Activity of PET2, 5, 6, and 12 was verified through assays on agar plates containing PET nanoparticles—all four enzymes showed activity.

Furthermore, PET2 and PET6 were subjected to additional activity assays and biochemical characterization. PET2 showed high thermal stability, since it maintained PET degrading activity up to 90 °C, which is highly promising given the amorphous state of PET polymer at this temperature [244].

The initial search across several databases revealed the most promising candidates, selected from similarities with experimentally proven PET degrading enzymes, are mainly from three phyla: Proteobacteria, Actinobacteria, and Bacteroidetes. Furthermore, most sequences with high similarity originate from samples containing crude oil, similar to how *Sc*Sub1 was first discovered [244].

Database query for novel PET degrading enzymes followed a set of criteria inspired by enzymes with well characterized activity on PET, namely the presence of a conserved Ser-His-Asp catalytic triad in the vicinity of an oxyanion hole made up of a Met residue and an aromatic residue, a terminal disulfide bridge essential for enzymatic thermostability, and a conserved DxDxR(Y)xxF(L)C sequence near the first cysteine of the disulfide bridge. Even though no further characterization of these enzymes has been published, the experimentally validated metagenomic methodology search used and the most promising enzymes identified suggest an encouraging path for identifying novel enzymes and microorganisms with relevant PET degrading activity.

All 13 PET hydrolytic enzymes identified by Danso et al. [244] are described in the UniProt database—Table 11 summarizes the UniProt access codes and general sequence characterization for these enzymes. There are no three-dimensional structures available for these proteins, and therefore specific structural motifs cannot be characterized and described with certainty. However, given the privileged knowledge of the amino acid sequence of each enzyme, several in silico sequence-based structure prediction methods could be used to combat the lack of an experimentally solved structure and allow for a deeper understanding of the determined activity rates and thermostability.

From the 13 PET enzymes, PET2, 5, 6, and 12 were tested for PET degrading activity against agar plates also containing nanoparticles of PCL. PCL was used as a model substrate, as the hydrolase ability for this substrate correlates with activity on higher complexity PET substrates. All four enzymes exhibited activity and produced halos in the plates. Furthermore, PET2 retained 80% of relative activity at 90 °C after an incubation period of 5 h with a series of *p*NP esters, meaning it is even more stable than LCC.

PET2 ability to remain active at higher drastic temperatures makes it an extremely attractive enzyme for PET degradation. When 100 μg of enzyme were tested with 14 mg amorphous PET as substrate, 900 μM of TPA were produced after a 24 h period incubation, confirming PET2 demonstrates interesting PET degrading activity and is able to fully degrade it to TPA.

#### 2.15.7. *Thermomonospora curvata* Cutinases 0390 and 1278 (*Tc*Cut0390 and *Tc*Cut1278)

*Tc*Cut0390 and *Tc*Cut1278, aka Tcur0390 and Tcur1278, are two cutinases from *Thermomonospora curvata* sharing 82% sequence identity [245]. *T. curvata* is a facultative aerobic thermophilic bacterium first isolated from plant composts [246,247,248]. Bacterial optimal growth conditions are pH from 7.5 to 11 and a temperature of 50 °C, but weak growth has been registered up to 65 °C [245].

Wei et al. [245] isolated and expressed these proteins and characterized their structure from an homology model. Further molecular docking and molecular dynamics studies allowed for binding site and structural stability characterization. Both *Tc*Cut0390 and *Tc*Cut1278 assume a canonical α/β-hydrolase fold with a catalytic triad composed of residues Ser130, His207, Asp176. The catalytic residues and the substrate-binding groove are solvent-exposed and located in the surface of the proteins. *Tc*Cut1278 is more thermally stable than *Tc*Cut0390, and they both hydrolyze *p*NPB and PET nanoparticles. Given the low thermal stability of both enzymes at temperatures higher than 55 °C, their applicability in PET degradation campaigns is highly conditioned and dependent on future engineering efforts to increase enzymatic catalytic turnover and melting temperatures.

The ability of *Tc*Cut0390 and *Tc*Cut1278 to hydrolase polyurethane (PU) has recently been characterized by Islam et al. [249] and Schmidt et al. [250].

#### 2.15.8. *Thermobifida halotolerans* Esterase (ThEst)

*Th*Est, aka Thh_Est, is an esterase from *Thermobifida halotolerans*, which was characterized as a PET degrading enzyme by Ribitsh et al. [54]. This 262 amino acid enzyme is highly similar to *Ta*Est119 and several cutinases from *T. cellulosilytica*. Sequence analysis led to the prediction of an α/β-serine hydrolase fold, with residue Ser131 as the catalytic triad serine, inserted in the typical GxSxG motif. Polyester hydrolysis ability of *Th*Est was firstly tested with 3PET in a 2 h activity assay at 50 °C and pH 7.0. Degradation of 3PET resulted in the release of benzoic acid (BA) and 2-hydroxyethyl benzoate (HEB) as the main products, and MHET and TPA in residual amounts, which suggests an exo-type hydrolysis mechanism. Consequently, degradation of PET film resulted in TPA and MHET release with no BHET detected, indicating *Th*Est was able to degrade PET to completion. Comparisons with *Thermobifida* cutinases revealed similar degradation potentials and rates, differing from the trend of esterases being less efficient PET hydrolases than cutinases.

## 3. Microorganisms with PET Degradation Activity

The evidence that a given organism can degrade PET or other polyester materials and use them as a carbon source frequently leads to the identification of a PET degrading enzyme. However, in many cases it is not possible (or it has not yet been done) to identify or isolate the specific enzyme responsible for PET depolymerization. In this section, some of these microorganisms are described.

Liebminger et al. [251] screened for microorganisms with PET degradation activity by incubating environmental samples with 3PET as the only carbon source for 10 days. From the active samples, a fungal strain identified as *Penicillium citrinum* showed the best growth. Although an enzyme was purified, characterization and identification of this presumed polyesterase was not possible. The experiments with PET fabric and 3PET as substrates revealed optimal conditions for enzymatic activity to be 30 °C and pH 8.2. The enzyme successfully altered the surface of the PET fabric and degraded 3PET to TPA, MHET, BHET, and BA. Given the described results, it is to be expected that further biochemical and structural characterization of this novel polyesterase may reveal a new promising PET degrading enzyme.

Costa et al. [252] showed *Yarrowia lipolytica*, a widely used yeast, had PET degrading activity. *Y. lipolytica* was cultured with amorphous PET, PET oligomer 3, post-consumer PET (with high crystallinity), and PET monomers TPA, monoethylene glycol (MEG), and BHET. Highest activity was registered for the amorphous PET samples, with MHET as the main product and TPA as a residual released product. This suggests MHET has an inhibitory effect on the yeast, hindering complete degradation of PET to TPA. Although the yeast was able to use the monomers as a carbon source in the absence of other substrates, the accumulation of PET degradation products seemed to be toxic for yeast growth, and consumption rate was higher for PET oligomers than for the monomers. The described results show that *Yarrowia lipolytica* is able to express PET degrading enzymes, as evidenced by the release of MHET in large quantities, and further isolation and expression of these enzymes is of high importance for future PET degrading yeast studies.

Arguing that using individual purified enzymes to break down PET might not be the most promising strategy, Roberts et al. [253] showed that a bacterial consortia containing *Bacillus* and *Pseudomonas* species able to use PET as a sole carbon source resulted in more effective PET degradation and surface modification activity. The consortia were identified in a screening of soil samples for lipase activity, and several consortia were built and further analyzed after initial lipase activity was confirmed. PET hydrolytic activity was demonstrated by the depolymerization of amorphous PET to BHET, TPA, and EG by the bacterial consortia. Individual strains identified within the consortia were tested as well, but activity and product release were always slower and lower than in the assays with the full consortia. It was therefore concluded that the bacterial strains in the consortia acted on PET in a synergistic manner, and complete degradation was only possible in the presence of all bacterial strains and respective secreted enzymes. Identification of individual secreted enzymes was not possible, which the authors attributed to biofilm formation within the consortia, and natural tendency of the secreted enzymes to bind the PET sample, hindering isolation.

## 4. Potential PET Hydrolases with Activity to Be Confirmed

*Ao*Cut, a cutinase from *Aspergillus oryzae*, has been shown to degrade PCL and theorized to have a similar effect on PET [254,255]. The two three-dimensional structures available for *Ao*Cut (PDB: 3GBS and 3QPD) reveal an α/β-serine hydrolase fold, with a central β sheet flanked by α helixes on either side. The catalytic triad (Ser126, Asp181, His194) and oxyanion hole (Ser48 and Gln127) are similar to the reported residues in typical enzymes with PET degrading activity. The two major structural differences that differentiate *Ao*Cut are *Fs*Cut the additional disulfide bridge (Cys63–Cys76, in addition to the two well conserved Cys37–Cys115 and Cys177–Cys184) and a longer and deeper groove near the active site. These features could explain the higher thermal stability of *Ao*Cut, and also higher activity on PCL [255]. Efforts by Shirke et al. [254] to increase thermal stability via rational mutation design have been successful in increasing T_m_ by 6 °C, but with a negative effect on PCL degrading activity. It is widely suggested that *Ao*Cut will have a hydrolytic effect on PET polymer, but only specific experimental activity assays can confirm this assumption.

Almeida et al. [256] built a reference data set with 15 highly studied PET degrading enzymes to scan a 52 genome sequence dataset of the *Streptomyces* genus. An esterase from *Streptomyces* sp., SM14est, was identified as having the highest potential as PETase-like enzyme, and was isolated, expressed and subjected to activity assays with PCL. Having degraded PCL, and given the presence of PETase-like characteristics, it is suggested that this enzyme is a potential PET hydrolase. A homology model revealed a structure similar to most PET degrading cutinases, with nine β-sheets and seven α-helixes. The catalytic triad was identified as Ser156, Asp202, His234; however, no disulfide bridge was predicted for the enzyme. Comparisons with *Is*PETase structures revealed a similar binding subsite I, but different binding subsite II. Molecular docking calculations predicted a binding mode for BHET like the one observed in *Is*PETase, supporting the authors’ thesis that SM14est is likely a PET degrading enzyme. However, besides the obvious need for experimental confirmation, the absence of any disulfide bond is a never-seen feature in PET hydrolases, and might greatly hinder thermal stability and, consequently, activity.

Huang et al. [257] reported an esterase from *Thermobifida fusca* named *Tf*AXE, with high similarity with *Tf*HCut and other highly active PET degrading enzymes. Even though specific activity against PET or other polyester polymers has not yet been described for this enzyme, it is consensually regarded as a likely PETase-like enzyme and considered of major interest in state-of-the-art PET bioremediation studies.

### 4.1. Thermobifida alba Esterase 119 (TaEst119)

This enzyme is closely related with TaEst1, a confirmed PET hydrolase, and is therefore a highly promising PET degrading enzyme.

#### 4.1.1. Discovery

*Ta*Est119 is an esterase from *Thermobifida alba*, a mesophilic bacterium [247]. The strain AHK119 possesses two tandem genes codifying two highly similar enzymes—*Ta*Est119 and *Ta*Est1. These enzymes share 95% identity and 98% similarity [45,210].

*Ta*Est119 has a molecular weight of 30 kDa, 300 amino acids, and was firstly identified in 2010 by Hu et al. [258] that described its potential as a PETase-like enzyme, even though no specific assays with PET related substrates were reported. However, Thumarat et al. [57] confirmed *Ta*Est119 activity on agar plates containing the polyesters PCL, PBSA, PBS, PDLA, PLLA, and PHB. The molecular docking of PET with a *Ta*Est119 three-dimensional structure was also reported by Thuramat, showing a good fitting of the docked PET unit in the active site for optimal catalyses.

#### 4.1.2. Structure

In 2012, Thuramat et al. [57] predicted the *Ta*Est119 structure from its sequence by modeling a 3D structure based on the well characterized homologous lipase, *S. exfoliatus* M11. The group reported that the enzyme displays an α/β-hydrolase fold with central nine stranded parallel β-sheets flanked by seven α-helices on both sides. Existent crystallographic structures for *Ta*Est119 are summarized in Table 12. Later in the same year, the first crystallographic structure was reported by Kitadokoro et al. [57] with a resolution of 1.76 Å. The structure confirmed that this enzyme exists as a monomer and exhibits an α/β-hydrolase fold with a central twisted β-sheet of nine β-strands, flanked by nine α-helices on both sides. The catalytic triad, which is located in the loops between the β-sheets and helices, is composed of Ser169, His247, and Asp215. The oxyanion hole is made up of amino acid residues Met170 and Tyr99. However, in a later report by Thumarat et al. [210], Met166 and Tyr99 are suggested as the residues comprising the oxyanion hole. A disulfide bond (Cys280–Cys298) is displayed in the C-terminal region.

A crystallographic structure was also developed by Kawai et al. [45] with a resolution of 1.68 Å. Two Ca^2+^ binding sites were shown to occupy a position in the loop regions α7–β9 and β9–C-terminal. Lastly, Kitadokoro et al. [260] determined the structure complexed with ethyl lactate (EL) with a resolution of 1.3 Å, in 2018. Here, the group confirmed the existence of the previously reported Ca^2+^ binding sites, also reporting two other Ca^2+^ binding sites, amounting to a total of 4 Ca^2+^ binding sites. Studies on the active site revealed it is formed by a long narrow groove. The terminal side of the groove is comprised of Tyr99, Met170, Trp194, and Ile217, which involve lactate (LAC). Tyr99, Ile217, and Phe248 also form hydrophobic interactions with EL.

#### 4.1.3. Activity

The optimal conditions for *Ta*Est119 activity have been reported to be around 45 to 55 °C and pH 6.0, where the enzyme loses 50% of its activity at 60 °C [258].

Among the *p*-nitrophenyl acyl esters, *Ta*Est119 revealed higher enzymatic activity towards *p*NPB substrate, resulting in a k_cat_ of 4.48 ± 0.21 s^−1^ [57], and a specific activity of 2.30 ± 0.02 U/mg in the absence of Ca^2+^ and 8.29 ± 0.03 U/mg in the presence of 300 mg of Ca^2+^ [210].

The substitution of the N-terminus residues correlated to higher enzymatic activity towards *p*NPB [57]. Replacing Ala68 with isoleucine, valine, or leucine results in an increase in hydrophobicity; however, the opposite is observed when the residue is replaced by tyrosine, tryptophan, or proline. The variant A68V resulted in the highest activity. The thermostability was significantly increased when Ser219, located in the substrate-docking loop, was replaced with proline (S219P). The double mutant (A68V/S219) resulted in a specific activity of 115 ± 3.5 and 299 ± 10.1 U/mg in the absence and presence of Ca^2+^, respectively [210]. In the presence of Ca^2+^ the enzymatic activity and thermostability were remarkably enhanced, suggesting that even though the secondary structure is not affected by Ca^2+^, the cations stabilize the tertiary protein structure [45,57,210,260].

#### 4.1.4. Proposed Mechanism

No catalytic mechanism towards PET degradation has been reported. However, Kitadokoro et al. [260] proposed the mechanism for ethyl lactate, which is the analogue of another plastic polymer, polylactic acid (PLA).

According to their proposal, His247 deprotonates Ser169, which becomes a nucleophile able to attack the carbonyl carbon atom of EL, resulting in the movement of a pair of electrons from the double bond of the carbonyl oxygen towards the oxygen, which are stabilized by the main-chain nitrogen atoms comprising the oxyanion hole (Tyr99 and Met170). This process results in the formation of a tetrahedral intermediate. The covalent electrons of the ester bond between the oxygen and the carbon in EL attack the hydrogen of Hys247, breaking the ester bond. The electrons that previously moved from the carbonyl oxygen double bond restore the double bond, leading to the collapse of the tetrahedral intermediate, which forms an acyl-enzyme intermediate and ethanol. The same pair of electrons move again to the oxygen and a water molecule bonds to the carbon atom of the acyl-enzyme. The hydrolytic attack by the water molecule towards the nitrogen of His247 leads to another intermediate and the release of ethanol. Lastly, the proton originated from the hydrolytic attack is transferred to Ser169, regenerating the catalytic triad, and thus releasing lactate.

#### 4.1.5. Future Perspectives

To implement *Ta*Est119 in the plastic-degrading industry for catalyses of PET substrates, enzymatic activity and thermostability need to be enhanced. For a better understanding of the enzymatic activity, studies of *Ta*Est119 complexed with PET substrates should be developed.

## 5. Summary and Outlook

As awareness increases about how plastics affect human health and the environment, so do the efforts by the scientific community in finding solutions. The evolution of bioengineering and bioremediation technology and research has allowed for the progressive discovery of several enzymes with plastic degradation ability, mainly for PET, as one of the most produced, used, and discarded plastics.

Although several of these enzymes are highly promising, their activities and thermal stabilities are still far from the desirable thresholds for efficient and large-scale applicability. A summary of the best engineering efforts for the described enzymes is presented in Table 13. Many of the enzymes with highly characterized structures are yet to be the target of mutagenic studies, and their potential activities remain to be explored. More studies are required to unharness the full potential of these enzymes for PET degradation.

## Figures and Tables

**Table 1:** Crystallographic structures of IsPETase.

PDB Code	Resolution (Å)	Ligand	Mutations	Year of Deposition	Ref.
5XG0	1.58	Free	-	2017	[76]
5XFY	1.40	Free	S131A	2017	[76]
5XFZ	1.55	Free	R103G/S131A	2017	[76]
5ZH3	1.30	HEMT	R103G/S131A	2017	[76]
5XH2	1.20	*p*NP	R103G/S131A	2017	[76]
5XJH	1.54	Free	-	2017	[74]
5YNS	1.36	Free	R280A	2017	[74]
6EQD	1.70	Free	-	2017	[75]
6EQH	1.58	Free	-	2017	[75]
6EQG	1.799	Free	-	2017	[75]
6EQF	1.70	Free	-	2017	[75]
6EQE	0.92	Free	-	2017	[75]
6ANE	2.02	Free	-	2017	[79]
5YFE	1.39	Free	-	2017	[78]
6ILW	1.575	Free	-	2018	[34]
6ILX	1.45	Free	W159F	2018	[34]
6QGC	2.0	Free	-	2019	[80]
6IJ3	1.40	Free	S121D/D186H	2019	[81]
6IJ4	1.86	Free	S121D/D186H	2019	[81]
6IJ5	1.72	Free	P181A	2019	[81]
6IJ6	1.95	Free	S121E/D186H/R280A	2019	[81]
6KY5	1.63	Free	S214H/I168R/W159H/S188Q/R280A/A180I/G165A/Q119Y/L117F/T140D	2019	[82]
6KUO	1.90	Free	N246D	2019	[83]
6KUQ	1.91	Free	A248D/R280K	2019	[83]
6KUS	2.00	Free	S121E/D186H/S242T/N246D	2019	[83]

**Table 2:** Crystallographic structures of IsMHETase.

PDB Code	Resolution (Å)	Ligand	Mutations	Year of Deposition	Ref
6QGA	2.1	MHETA	-	2019	[80]
6QGB	2.2	Benzoic acid	-	2019	[80]
6QG9	2.05	Free	-	2019	[80]
GJTU	2.1	Free	-	2019	[95]
6JTT	2.51	BHET	-	2019	[95]
6QZ1	1.7	Benzoic acid	-	2019	[89]
6QZ2	1.9	Benzoic acid	-	2019	[89]
6QZ3	1.6	Free	-	2019	[89]
6QZ4	1.8	Free	-	2019	[89]

**Table 3:** Crystallographic structures of PaPETase.

PDB Code	Resolution (Å)	Ligand	Mutation	Year of Deposition	Ref.
6SBN	1.09	-	-	2019	[97]
6SCD	1.35	-	Y250S	2019	[97]

**Table 4:** Crystallographic structures of LCC.

PDB Code	Resolution (Å)	Ligand	Mutations	Year of Deposition	Ref.
4EB0	1.5	Free	-	2012	[105]
6THS	1.10	Free	S165A	2019	[106]
6THT	1.14	Free	Y127G/S165A/D238C/F243I/S283C	2019	[106]

**Table 5:** Crystallographic structures of SvCut190.

PDB Code	Resolution (Å)	Ligand	Mutation	Year of Deposition	Ref.
4WFK	2.35	Free	S226P Ca^2+^ bound	2014	[132]
4WFI	1.45	Free	S226P Ca^2+^ free	2014	[132]
4WFJ	1.75	Free	S226P Ca^2+^ bound	2014	[132]
5ZNO	1.6	Free	S176A/S226P/R228S	2018	[133]
5ZRQ	1.12	Free	S176A/S226P/R228S	2018	[133]
5ZRR	1.34	Monoethyl succinate	S176A/S226P/R228S	2018	[133]
5ZRS	1.4	Monoethyl adipate	S176A/S226P/R228S	2018	[133]
7CEH	1.09	Free	S176A/S226P/R228S with C-terminal three residues deletion	2020	[135]
7CEF	1.6	Free	S226P/R228S with C-terminal three residues deletion	2020	[135]
7CTR	1.20	Free	S226P/R228S/Q138A/D250C–E296C/Q123H/N202H	2020	[136]
7CTS	1.10	Free	S176A/S226P/R228S/Q138A/D250C–E296C/Q123H/N202H	2020	[136]

**Table 6:** Structures of Cut1 and Cut2.

PDB Code	Enzyme	Resolution (Å)	Ligand	Mutation	Year of Deposition	Ref.
4CG1	*Tf*Cut2	1.4	Free	-	2013	[141]
4CG2	*Tf*Cut2	1.44	PMSF	-	2013	[141]
4CG3	*Tf*Cut2	4.55	Free	-	2013	[141]
5LUK	*Tc*Cut2	1.45	Free	R28N/A30V	2016	[142]
5LUJ	*Tc*Cut2	2.2	Free	-	2016	[142]
5LUL	*Tc*Cut2	1.9	Free	R19S/R29N/A30V	2016	[142]
5LUI	*Tc*Cut1	1.5	Free	-	2016	[142]

**Table 7:** Crystallographic structures of HiCut.

PDB Code	Resolution (Å)	Ligand	Mutation	Year of Deposition	Ref.
4OYY	3	-	-	2014	[159]
4OYL	2.05	Mono-ethyl phosphate	-	2014	[159]

**Table 8:** Crystallographic structures of FsCut.

PDB Code	Resolution (Å)	Ligand	Mutations	Year of Deposition	Ref.
1CUS	1.25	-	-	1994	[174]
2CUT	1.90	Diethyl Phosphonate	-	1994	[177]
1FFA	1.69	-	N84A	1995	[178]
1FFB	1.75	-	N84D	1995	[178]
1FFC	1.75	-	N84L	1995	[178]
1FFD	1.69	-	N84W	1995	[178]
1FFE	1.69	-	S42A	1995	[178]
1XZA	1.80	-	S129C	1995	[179]
1XZB	1.75	Mercury Acetate	S129C	1995	[179]
1XZC	1.75	Para-Sulfurous phenyl mercury	S129C	1995	[179]
1XZD	2.70	-	S213C	1995	[179]
1XZE	1.75	-	S92C	1995	[179]
1XZF	1.69	-	T144C	1995	[179]
1XZG	1.69	-	T45A	1995	[179]
1XZH	1.69	-	T80P	1995	[179]
1XZI	1.69	-	Y119H	1995	[179]
1XZJ	1.69	-	T38F	1995	[179]
1CUA	1.80	-	N172K	1995	[179]
1CUB	1.75	-	N172K/R196D	1995	[179]
1CUC	1.75	-	N172K/R196D	1995	[179]
1CUD	2.70	-	N172K/R196D	1995	[179]
1CUE	2.10	-	Q121L	1995	[179]
1CUF	1.75	-	R156L	1995	[179]
1CUG	1.75	-	R17E/N172K	1995	[179]
1CUH	1.75	-	R196E	1995	[179]
1CUI	2.50	-	S120A	1995	[179]
1CUJ	1.60	-	S120C	1995	[179]
1CUU	1.69	-	A199C	1995	[179]
1CUV	2.01	-	A85F	1995	[179]
1CUW	2.70	-	G82A/A85F/V184I/L189F	1995	[179]
1CUX	1.75	-	L114Y	1995	[179]
1CUY	1.69	-	L189F	1995	[179]
1CUZ	2.10	-	L81G/L182G	1995	[179]
1XZK	2.01		-	1995	[179]
1XZL	1.69	N-hexylphosphonate ethyl ester	-	1995	[179]
1XZM	1.75	N-undecyl o-methyl chloro Phosphonate ester	-	1995	[179]
1OXM	2.30	triglyceride analogue ([(2*R*)-2-(butylcarbamoyloxy)-3-butylphosphonoyloxypropyl] *N*-butylcarbamate)	-	1996	[180]
1AGY	1.15	-	-	1997	[181]
1CEX	1.00	-	-	1997	[181]
3EF3	1.50	-	N172K	2008	[182]
3ESA	2.00	-	N172K	2008	[182]
3ESB	2.30	-	N172K	2008	[182]
3ESC	1.20	triglyceride analogue (ethyl 4-nitrophenyl P-[3-(4-(bromopallado)-1,3-bis[(methylthio)methyl]-phenyl)propyl]phosphonate)	N172K	2008	[182]
3ESD	1.22	triglyceride analogue (ethyl 4-nitrophenyl P-[3-(4-(bromopallado)-1,3-bis[(methylthio)methyl]-phenyl)propyl]phosphonate)	N172K	2008	[182]
3QPA	0.85	-	-	2011	N/A
3QPC	0.98	-	-	2011	N/A

**Table 9:** Crystallographic structures of CaLipB.

PDB Code	Resolution (Å)	Ligand	Mutations	Year of Deposition	Ref.
1TCA	1.55	-	-	1994	[192]
1TCB	2.10	-	-	1994	[192]
1TCC	2.5	-	-	1994	[192]
1LBS	2.60	Phosphonate inhibitor	-	1995	[188]
1LBT	2.50	Tween 80	-	1995	[188]
3ICV	1.49	-	-	2009	[194]
3ICW	1.69	Inhibitor (methyl hydrogen (R)-hexylphosphonate)	-	2009	[194]
3W9B	2.90	-	-	2013	N/A
4K5Q	1.49	-	D223G/L278M	2013	[195]
4K6K	1.60	-	D223G	2013	[195]
4K6H	1.60	-	L278M	2013	[195]
4K6G	1.50	-	-	2013	[195]
4ZV7	2.00	-	-	2015	[195]
5A6V	2.28	-	-	2015	[191]
5A71	0.91	-	-	2015	[191]
5GV5	2.89	-	-	2016	[193]
6ISP	1.88	-	W104V/A281Y/A282Y/V149G	2018	[196]
6ISQ	1.86	-	W104V/S105C/A281Y/A282Y/V149G	2018	[196]
6ISR	2.6	-	W104V/S105C/A281Y/A282Y/V149G	2018	[196]
6J1P	1.76	-	A281G/A282V/V190C	2018	[197]
6J1R	1.6	-	Q157L/I189A	2018	[197]
6J1Q	1.6	-	W104A/I189V	2018	[197]
6J1T	1.78	Synthesized product 3a’ ((2S)-2-phenyl-N-[(1R)-1-phenylethyl]propanamide)	A281G/A282V/V190C	2018	[197]
6J1S	1.83	-	W104A/I189M/V190C/D134L	2018	[197]
6TP8	1.55	-	-	2019	[198]

**Table 10:** Crystallographic structures of TlLip.

PDB Code	Resolution (Å)	Ligand	Mutation	Year of Deposition	Ref.
1TIB	1.84	Free	-	1993	[220]
1DT3	2.6	Free	-	2000	[224]
1DT5	2.4	Free	-	2000	[224]
1DTE	2.35	Free	-	2000	[224]
1DU4	2.5	Free	-	2000	[224]
1EIN	3	didodecyl phosphatidylcholine	-	2000	[224]
1GT6	2.2	Oleic acid	S146A	2002	[225]
4GYH	2	Free	-	2012	N/A
4EA6	2.3	Free	-	2012	N/A
4FLF	2.15	TPP	-	2012	[226]
4GBG	2.9	Free	-	2012	N/A
4GHW	2.6	decanoic acid	-	2012	N/A
4GI1	2.43	16-hydroxypalmitic acid	-	2012	N/A
4GLB	2.69	p-nitrobenzaldehyde	-	2012	N/A
4GWL	2.55	Free	-	2012	N/A
4KJX	2.1	p-nitrobenzaldehyde and lauric acid	-	2013	N/A
4N8S	2.3	p-nitrobenzaldehyde and ethyl acetoacetate	-	2013	N/A
4S0X	2.1	Lauric acid	-	2015	N/A
4ZGB	2.3	Free	-	2015	[226]
5AP9	1.8	Free	I186C/I255C	2015	[227]
6HW1	2.5	Free	-	2018	[228]
6OR3	1.45	Palmitic acid	-	2020	[229]
6XOK	1.3	2-hydroxy-3-(octadecanoyloxy)propyl pentacosanoate	-	2020	[229]
6XRV	1.43	2-hydroxy-3-(octadecanoyloxy)propyl pentacosanoate and caprylic acid	-	2020	[229]
6XS3	2.48	2-hydroxy-3-(octadecanoyloxy)propyl pentacosanoate and caprylic acid	-	2020	[229]

**Table 11:** Summary of information known of enzymes PET 1–13.

Pet No.	UniProt Code	Sequence Length	Organism
1	E8U721	315	*Deinococcus maricopensis*
2	C3RYL0	308	uncultured bacterium
3	A0A0F9X315	300	marine sediment metagenome
4	N6VY44	295	*Marinobacter nanhaiticus*
5	R4YKL9	310	*Oleispira antarctica*
6	A0A1Z2SIQ1	298	*Vibrio gazogenes*
7	Q8RR62	304	*Acidovorax delafieldii*
8	P19833	319	*Moraxella* sp.
9	A0A0D4L7E6	313	*Psychrobacter* sp.
10	UPI00064655D2	292	*Methylibium* sp.
11	UPI0003660256	292	*Caldimonas manganoxidans*
12	A0A0G3BI90	298	*[Polyangium] brachysporum*
13	A0A1F4G492	283	*Burkholderiales bacterium*

**Table 12:** Crystallographic structures of TaEst119.

PDB Code	Resolution (Å)	Ligand	Mutation	Year of Deposition	Ref.
3VIS	1.76	-	-	2011	[259]
3WYN	1.68	-	-	2014	[45]
6AID	1.3	Ethyl lactate	-	2018	[260]

**Table 13:** Mutations corresponding to the highest activity and best performance for several of the main PET degrading enzymes described.

Enzyme	Best Performing Mutation
*Is*PETase	S214H/I168R/W159H/S188Q/R280A/A180I/G165A/Q119Y/L117F/T140D
*Is*MHETase	W397A
*Pa*PETase	Y250S
*LCC*	F243I/D238C/S283D/Y127G
	F243I/D238C/S283C/T96M
	F243W/D238C/S283C/Y127G
	F243W/D238C/S283C/T96M
*Tf*HCut	Q132A/T101A
*Sv*Cut190	S226P/R228S/Q138A/D250C–E296C/Q123H/N202H
*Tf*Cut2	D204C/E253C/D174R
*Tc*Cut2	R29N/A30V
*Fs*Cut	L182A
	L81A
	V184A
*Ta*Est1	A68V/T253P

## Supplementary Materials

The following are available online at https://www.mdpi.com/article/10.3390/ijms222011257/s1, Table S1: Mutagenesis assays with *Is*PETase, Table S2: Mutagenesis assays with *Is*MHETase, Table S3: Mutagenesis assays with *Pa*MHETase, Table S4: Mutagenesis assays with LCC, Table S5: Mutagenesis assays with *Tf*HCut, Table S6: Mutagenesis assays with *Tc*Cut, Table S7: Mutagenesis assays with *Tl*Lip, Table S8: Mutagenesis assays with *Sv*Cut190, Table S9: Mutagenesis assays with *Tf*Ca, Table S10: Mutagenesis assays with *Ta*Est1.
