# Supplementary material for: Perspectives on the Role of Enzymatic Biocatalysis for the Degradation of Plastic PET
Source: Int J Mol Sci. 2021 Oct 19;22(20):11257. doi: 10.3390/ijms222011257 (PMC8540959; doi:10.3390/ijms222011257)
Supplement: Supplementary file 1 [file ijms-22-11257-s001.zip › ijms-1414419-supplementary.pdf]

International Journal of *Molecular Sciences*

MDPI

Supplementary Information

Perspectives on the role of Enzymatic Biocatalysis for the Degradation of Plastic PET

Rita P. Magalhães [1,2], Jorge M. Cunha [1,2] and Sérgio F. Sousa [1,2,*]

1 UCIBIO – Applied Molecular Biosciences Unit, BioSIM – Departamento de Biomedicina, Faculdade de Medicina da Universidade do Porto;
2 Associate Laboratory i4HB – Institute for Health and Bioeconomy, Faculdade de Medicina, Universidade do Porto, Portugal;
* Correspondence: sergiofsousa@med.up.pt

Table S1: Mutagenesis assays with *Is*PETase

Mutation	Rationale	Assay Results	Substrate	Assay Conditions	REF
C203S	DS1 disrupting mutation	Very low activity	PET Film	pH=9.0 T=30 ºC t=42 h	[1]
C239S	DS1 disrupting mutation	Very low activity	PET Film	pH=9.0 T=30 ºC t=42 h	[1]
W185A	Confirm residue role in catalysis/activity	Very low activity	PET Film	pH=9.0 T=30 ºC t=42 h	[1]
S214H	Confirm residue role in catalysis/activity	Diminished activity	PET Film	pH=9.0 T=30 ºC t=42 h	[1]
I208A	Substrate Interacting Residue	Diminished activity	PET Film	pH=9.0 T=30 ºC t=42 h	[1]
W159A	Substrate Interacting Residue	Diminished activity	PET Film	pH=9.0 T=30 ºC t=42 h	[1]
M161A	Substrate Interacting Residue	Diminished activity	PET Film	pH=9.0 T=30 ºC t=42 h	[1]
Y87A	Substrate Interacting Residue	Diminished activity	PET Film	pH=9.0 T=30 ºC t=42 h	[1]
T88A	Substrate Interacting Residue	Diminished activity	PET Film	pH=9.0 T=30 ºC t=42 h	[1]
W168H	Consistent Histidine residue in similar enzymes	Significantly diminished activity	PET Film	pH=9.0 T=30 ºC t=42 h	[1]
S160A	Confirm role as catalytic triad residue	Complete loss of activity	BHET	pH=7.0 T=30 ºC	[2]
D206A	Confirm role as catalytic triad residue	Complete loss of activity	BHET	pH=7.0 T=30 ºC	[2]
H237A	Confirm role as catalytic triad residue	Complete loss of activity	BHET	pH=7.0 T=30 ºC	[2]
S160A	Confirm role as catalytic triad residue	Complete loss of activity	PET Film	pH=9.0 T=30 ºC	[2]
D206A	Confirm role as catalytic triad residue	Complete loss of activity	PET Film	pH=9.0 T=30 ºC	[2]
H237A	Confirm role as catalytic triad residue	Complete loss of activity	PET Film	pH=9.0 T=30 ºC	[2]
Y87A	Subsite I residue / Oxanion Hole	5% of WT hydrolytic activity	BHET	pH=7.0 T=30 ºC	[2]
Y87A	Subsite I residue / Oxanion Hole	Diminished activity	PET Film	pH=9.0 T=30 ºC	[2]
M161A	Subsite I residue / Oxanion Hole	52% of WT hydrolytic activity	BHET	pH=7.0 T=30 ºC	[2]
M161A	Subsite I residue / Oxanion Hole	Diminished activity	PET Film	pH=9.0 T=30 ºC	[2]
W185A	Subsite I residue	5% of WT hydrolytic activity	BHET	pH=7.0 T=30 ºC	[2]
W185A	Subsite I residue	Diminished activity	PET Film	pH=9.0 T=30 ºC	[2]
I208A	Subsite I residue	46% of WT hydrolytic activity	BHET	pH=7.0 T=30 ºC	[2]
I208A	Subsite I residue	Diminished activity	PET Film	pH=9.0 T=30 ºC	[2]
W159A	Subsite II residue	8% of WT hydrolytic activity	BHET	pH=7.0 T=30 ºC	[2]
W159A	Subsite II residue	Diminished activity	PET Film	pH=9.0 T=30 ºC	[2]
S238A	Subsite II residue	Activity similar to WT	BHET	pH=7.0 T=30 ºC	[2]
S238A	Subsite II residue	Diminished activity	PET Film	pH=9.0 T=30 ºC	[2]
N241A	Subsite II residue	18% of WT hydrolytic activity	BHET	pH=7.0 T=30 ºC	[2]

N241A	Subsite II residue	Diminished activity	PET Film	pH=9.0 T=30 ºC	[2]
R280A	Subsite II residue	Activity similar to WT	BHET	pH=7.0 T=30 ºC	[2]
R280A	Subsite II residue	22.4% increased activity in 18h and 32.4% increased activity in 36h	PET Film	pH=9.0 T=30 ºC	[2]
C203S	DS1 disrupting mutation	Very low activity	BHET	pH=7.0 T=30 ºC	[2]
C239S	DS1 disrupting mutation	Very low activity	BHET	pH=7.0 T=30 ºC	[2]
C203S	DS1 disrupting mutation	Very low activity	PET Film	pH=9.0 T=30 ºC	[2]
C239S	DS1 disrupting mutation	Very low activity	PET Film	pH=9.0 T=30 ºC	[2]
S238F	Introduce residue present in TfCut2	Significantly diminished activity	BHET	pH=7.0 T=30 ºC	[2]
W159H	Introduce residue present in TfCut2	Significantly diminished activity	BHET	pH=7.0 T=30 ºC	[2]
S238F	Introduce residue present in TfCut2	Significantly diminished activity	PET Film	pH=9.0 T=30 ºC	[2]
W159H	Introduce residue present in TfCut2	Significantly diminished activity	PET Film	pH=9.0 T=30 ºC	[2]
S238F/W159H	Introduce residues present in TfCut2	Outperformed WT in crystallinity reduction and product release	PET Film	pH=7.2	[3]
W185A	Subsite I residue	Significantly diminished activity	PET Film	pH=7.2	[3]
S160A	Confirm role as catalytic triad residue	Complete loss of activity	BHET	pH=7.5 T=30 ºC	[4]
S160A	Confirm role as catalytic triad residue	Complete loss of activity	PET bottle	pH=9.0 T=30 ºC	[4]
D206A	Confirm role as catalytic triad residue	Complete loss of activity	BHET	pH=7.5 T=30 ºC	[4]
D206A	Confirm role as catalytic triad residue	Complete loss of activity	PET bottle	pH=9.0 T=30 ºC	[4]
H237A	Confirm role as catalytic triad residue	Complete loss of activity	BHET	pH=7.5 T=30 ºC	[4]
H237A	Confirm role as catalytic triad residue	Complete loss of activity	PET bottle	pH=9.0 T=30 ºC	[4]
W159A	Substrate Interacting Residue	Increased Activity	BHET	pH=7.5 T=30 ºC	[4]
W159A	Substrate Interacting Residue	Increased Activity	PET bottle	pH=9.0 T=30 ºC	[4]
W159H	Substrate Interacting Residue	Increased Activity	BHET	pH=7.5 T=30 ºC	[4]
W159H	Substrate Interacting Residue	Increased Activity	PET bottle	pH=9.0 T=30 ºC	[4]
M161A	Substrate Interacting Residue	Diminished activity	BHET	pH=7.5 T=30 ºC	[4]
M161A	Substrate Interacting Residue	Diminished activity	PET bottle	pH=9.0 T=30 ºC	[4]
W185A	Substrate Interacting Residue	Diminished activity	BHET	pH=7.5 T=30 ºC	[4]
W185A	Substrate Interacting Residue	Diminished activity	PET bottle	pH=9.0 T=30 ºC	[4]
A209I	Substrate Interacting Residue	Increased Activity	BHET	pH=7.5 T=30 ºC	[4]
A209I	Substrate Interacting Residue	Increased Activity	PET bottle	pH=9.0 T=30 ºC	[4]
Q119A	Substrate Interacting Residue	Diminished activity	BHET	pH=7.5 T=30 ºC	[4]

Q119A	Substrate Interacting Residue	Diminished activity	PET bottle	pH=9.0 T=30 ºC	[4]
S214H	Substrate Interacting Residue	Increased Activity	BHET	pH=7.5 T=30 ºC	[4]
S214H	Substrate Interacting Residue	Increased Activity	PET bottle	pH=9.0 T=30 ºC	[4]
S238F	Substrate Interacting Residue	Activity similar to WT	BHET	pH=7.5 T=30 ºC	[4]
S238F	Substrate Interacting Residue	Significantly diminished activity	PET bottle	pH=9.0 T=30 ºC	[4]
Y87A	Substrate Interacting Residue	Increased Activity	BHET	pH=7.5 T=30 ºC	[4]
Y87A	Substrate Interacting Residue	Increased Activity	PET bottle	pH=9.0 T=30 ºC	[4]
W97L	Active Site Residue (stabilizes regidity)	Significantly diminished activity	BHET	pH=7.5 T=30 ºC	[4]
W97L	Active Site Residue (stabilizes regidity)	Significantly diminished activity	PET bottle	pH=9.0 T=30 ºC	[4]
Q182L	Active Site Residue (stabilizes regidity)	Significantly diminished activity	BHET	pH=7.5 T=30 ºC	[4]
Q182L	Active Site Residue (stabilizes regidity)	Significantly diminished activity	PET bottle	pH=9.0 T=30 ºC	[4]
R123A	Active Site Residue (stabilizes regidity)	Significantly diminished activity	BHET	pH=7.5 T=30 ºC	[4]
R123A	Active Site Residue (stabilizes regidity)	Significantly diminished activity	PET bottle	pH=9.0 T=30 ºC	[4]
N241A	Active Site Residue (stabilizes regidity)	Significantly diminished activity	BHET	pH=7.5 T=30 ºC	[4]
N241A	Active Site Residue (stabilizes regidity)	Significantly diminished activity	PET bottle	pH=9.0 T=30 ºC	[4]
S93F	Reduce substrate specificity - attain degradation of naphtyl esters	Increased Activity agains 1-NP	1-Naphthyl butyrate	-	[5]
W159F	Reduce substrate specificity - attain degradation of naphtyl esters	Increased Activity agains 1-NP	1-Naphthyl butyrate	-	[5]
N242F	Reduce substrate specificity - attain degradation of naphtyl esters	Increased Activity agains 1-NP	1-Naphthyl butyrate	-	[5]
P181A	Residue disrupting secondary structure	Tm=49.25 ºC / Diminished activity	PET film	30 ºC and 40 ºC	[6]
S121D/D186H	Introduce residue present in TfCut2	Tm=54.85 ºC / Increased Activity by 2 fold after 24 h and 72 h	PET film	30 ºC	[6]
S121D/D186H	Introduce residue present in TfCut2	Tm=54.85 ºC / Increased Activity by 3.4 fold after 24 h and 4.4 fold at72 h	PET film	40 ºC	[6]
S121E/D186H	Improve previously discovered variant	Tm=56.02 ºC / Increased Activity by 2.9 fold after 24 h and 2.6 fold after 72 h	PET film	30 ºC	[6]
S121E/D186H	Improve previously discovered variant	Tm=54.85 ºC / Increased Activity by 4.5 fold after 24 h and 6.0fold at 72 h	PET film	40 ºC	[6]
S121D	Improve previously discovered variant	Slight activity increase at 30 ºC but lower at 40 ºC	PET film	30 ºC and 40 ºC	[6]
S121E	Improve previously discovered variant	Slight activity increase at 30 ºC but lower at 40 ºC	PET film	30 ºC and 40 ºC	[6]
D186H	Improve previously discovered variant	Slight activity increase	PET film	30 ºC and 40 ºC	[6]
D186F	Improve previously discovered variant	Slight activity increase	PET film	30 ºC and 40 ºC	[6]

P181G	Residue disrupting secondary structure	Diminished activity	PET film	30 ºC and 40 ºC	[6]
P181S	Residue disrupting secondary structure	Diminished activity	PET film	30 ºC and 40 ºC	[6]
P181A/S121D/D186H	Improve previously discovered variant	Tm=52.69 ºC / Increased Activity by 2 fold after 24 h and 1.6 after 72 h	PET film	30 ºC	[6]
P181A/S121D/D186H	Improve previously discovered variant	Tm=52.69 ºC / Increased Activity by 2.3 fold after 24 h and 2.9 after 72 h	PET film	40 ºC	[6]
P181A/S121E/D186H	Improve previously discovered variant	Tm=53.56 ºC / Increased Activity by 1.6 fold after 24 h and 1.2 after 72 h	PET film	30 ºC	[6]
P181A/S121E/D186H	Improve previously discovered variant	Tm=53.56 ºC / Increased Activity by 1.6 fold after 24 h and 2.3 after 72 h	PET film	40 ºC	[6]
S121D/D186H/R280A	Improve previously discovered variant	Tm=56.41 ºC / Increased Activity by 3.4 fold after 24 h and 5.0 after 72 h	PET film	30 ºC	[6]
S121D/D186H/R280A	Improve previously discovered variant	Tm=56.41 ºC / Increased Activity by 8.1 fold after 24 h and 10.8 after 72 h	PET film	40 ºC	[6]
S121E/D186H/R280A	Improve previously discovered variant	Tm=57.62 ºC / Increased Activity by 4.3 fold after 24 h and 5.2 after 72 h	PET film	30 ºC	[6]
S121E/D186H/R280A	Improve previously discovered variant	Tm=57.62 ºC / Increased Activity by 9.1 fold after 24 h and 13.9 after 72 h	PET film	40 ºC	[6]
S214H, I168R, W159H, S188Q, R280A, A180I, G165A, Q119Y, L117F, T140D	Consensus mutatiosn predicted through GRAPE strategy	Increased activity by over 300 fold	PET Film	37 ºC 10 days	[7]
Y87F	Substrate interacting residue replaced by residue present in other PETase candidates	Slightly diminished activity	PET bottle	pH=90 T=25 ºC and 37 ºC	[8]
T88L	Substrate interacting residue replaced by residue present in other PETase candidates	Highly diminished activity	PET bottle	pH=90 T=25 ºC and 37 ºC	[8]
R90S	Substrate interacting residue replaced by residue present in other PETase candidates	Highly diminished activity	PET bottle	pH=90 T=25 ºC and 37 ºC	[8]
I208V	Substrate interacting residue replaced by residue present in other PETase candidates	Slightly diminished activity	PET bottle	pH=90 T=25 ºC and 37 ºC	[8]
I208T	Substrate interacting residue replaced by residue present in other PETase candidates	Highly diminished activity	PET bottle	pH=90 T=25 ºC and 37 ºC	[8]
G234N	Substrate interacting residue replaced by residue present in other PETase candidates	Highly diminished activity	PET bottle	pH=90 T=25 ºC and 37 ºC	[8]
S238T	Substrate interacting residue replaced by residue present in other PETase candidates	Slightly diminished activity	PET bottle	pH=90 T=25 ºC and 37 ºC	[8]
S242T	Substrate interacting residue replaced by residue present in other PETase candidates	Significantly increased activity	PET bottle	pH=90 T=25 ºC and 37 ºC	[8]
N246D	Substrate interacting residue replaced by residue present in other PETase candidates	Significantly increased activity	PET bottle	pH=90 T=25 ºC and 37 ºC	[8]
S121E/D186H/N246D/R280A	Inserting N246D mutation into previously discovered	Increased activity compared to WT but lower than S121E/D186/R280A mutation	PET bottle	pH=90 T=25 ºC and 37 ºC	[8]

	high activity mutated enzyme				
S121E/D186H/S242T/N246D	Inserting N246D and S242T mutations into previously discovered high activity mutated enzyme	Increased activity by 58 fold at 37 ºC compared to WT	PET bottle	pH=90 T=25 ºC and 37 ºC	[8]
R61A	Substrate Interacting Residue	1.6 times greater product release compared to WT in same conditions	PET Film	pH=8.5 30 ºC 48h	[9]
L88F	Substrate Interacting Residue	2.1 times greater product release compared to WT in same conditions	PET Film	pH=8.5 30 ºC 48h	[9]
S178T	Substrate Interacting Residue	Diminished activity	PET Film	pH=8.5 30 ºC 48h	[9]
I179F	Substrate Interacting Residue	2.5 times greater product release compared to WT in same conditions	PET Film	pH=8.5 30 ºC 48h	[9]
I179V	Substrate Interacting Residue	Similar activity to WT	PET Film	pH=8.5 30 ºC 48h	[9]
S209V	Substrate Interacting Residue	Diminished activity	PET Film	pH=8.5 30 ºC 48h	[9]
S209F	Substrate Interacting Residue	Similar activity to WT	PET Film	pH=8.5 30 ºC 48h	[9]
A211P	Substrate Interacting Residue	Similar activity to WT	PET Film	pH=8.5 30 ºC 48h	[9]

Table S2: Mutagenesis assays with *Is*MHETase

Mutations	Rationale	Assay result	Substrate	Assay condition	REF
R411K	Active Site Residue	Significantly decreases activity	MHET	pH=7.5 T=30ºC	[10]
R411K	Active Site Residue	Significantly increases activity	BHET	pH=7.5 T=30ºC	[10]
F415S	Active Site Residue	Significantly decreases activity	MHET	pH=7.5 T=30ºC	[10]
F424D	Substrate Binding Hinderer	Very low activity	MHET	pH=7.5 T=30ºC	[10]
F424D	Substrate Binding Hinderer	Small increase in activity	BHET	pH=7.5 T=30ºC	[10]
F424E	Substrate Binding Hinderer	Very low activity	MHET	pH=7.5 T=30ºC	[10]
F424E	Substrate Binding Hinderer	Small increase in activity	BHET	pH=7.5 T=30ºC	[10]
F424H	Substrate Binding Hinderer	Very low activity	MHET	pH=7.5 T=30ºC	[10]
F424H	Substrate Binding Hinderer	Small increase in activity	BHET	pH=7.5 T=30ºC	[10]
F424I	Substrate Binding Hinderer	Significantly decreases activity	MHET	pH=7.5 T=30ºC	[10]
F424I	Substrate Binding Hinderer	Significantly increases activity	BHET	pH=7.5 T=30ºC	[10]
F424L	Substrate Binding Hinderer	Very low activity	MHET	pH=7.5 T=30ºC	[10]
F424L	Substrate Binding Hinderer	Small increase in activity	BHET	pH=7.5 T=30ºC	[10]
F424N	Substrate Binding Hinderer	Significantly decreases activity	MHET	pH=7.5 T=30ºC	[10]
F424N	Substrate Binding Hinderer	Significantly increases activity	BHET	pH=7.5 T=30ºC	[10]
F424T	Substrate Binding Hinderer	Very low activity	MHET	pH=7.5 T=30ºC	[10]
F424T	Substrate Binding Hinderer	Small increase in activity	BHET	pH=7.5 T=30ºC	[10]
F424V	Substrate Binding Hinderer	Significantly decreases activity	MHET	pH=7.5 T=30ºC	[10]
F424V	Substrate Binding Hinderer	Significantly increases activity	BHET	pH=7.5 T=30ºC	[10]
R411K/F424N	Double-mutant of previously described variants	Significantly decreases activity	MHET	pH=7.5 T=30ºC	[10]

R411K/F424N	Double-mutant of previously described variants	Significantly increases activity	BHET	pH=7.5 T=30ºC	[10]
R411K/F424V	Double-mutant of previously described variants	Significantly decreases activity	MHET	pH=7.5 T=30ºC	[10]
R411K/F424V	Double-mutant of previously described variants	Significantly increases activity	BHET	pH=7.5 T=30ºC	[10]
R411K/F424I	Double-mutant of previously described variants	Significantly decreases activity	MHET	pH=7.5 T=30ºC	[10]
R411K/F424I	Double-mutant of previously described variants	Significantly increases activity	BHET	pH=7.5 T=30ºC	[10]
F415H/F424N	Double-mutant of previously described variants	Significantly decreases activity	MHET	pH=7.5 T=30ºC	[10]
F415H/F424N	Double-mutant of previously described variants	Significantly increases activity	BHET	pH=7.5 T=30ºC	[10]
R411K/S416A/F424N	Triple-mutant of previously described variants	Significantly decreases activity	MHET	pH=7.5 T=30ºC	[10]
R411K/S416A/F424N	Triple-mutant of previously described variants	Significantly increases activity	BHET	pH=7.5 T=30ºC	[10]
S225A	Confirm role as catalytic triad residue	Complete loss of activity	MHET	pH=7.5 T=30ºC	[11]
S225A	Confirm role as catalytic triad residue	Complete loss of activity	MpNPT	pH=7.5 T=30ºC	[11]
S225A	Confirm role as catalytic triad residue	Complete loss of activity	BHET	pH=7.5 T=30ºC	[11]
D492A	Confirm role as catalytic triad residue	Complete loss of activity	MHET	pH=7.5 T=30ºC	[11]
D492A	Confirm role as catalytic triad residue	Complete loss of activity	MpNPT	pH=7.5 T=30ºC	[11]
H528A	Confirm role as catalytic triad residue	Complete loss of activity	MHET	pH=7.5 T=30ºC	[11]
H528A	Confirm role as catalytic triad residue	Complete loss of activity	MpNPT	pH=7.5 T=30ºC	[11]
W397A	Active Site Residue	Significantly increases activity	MHET	pH=7.5 T=30ºC	[11]
W397A	Active Site Residue	Small increase in activity	MpNPT	pH=7.5 T=30ºC	[11]
R411A	Active Site Residue	Very low activity	MHET	pH=7.5 T=30ºC	[11]
R411A	Active Site Residue	Slightly lower activity	MpNPT	pH=7.5 T=30ºC	[11]
R411A	Active Site Residue	Small increase in activity	BHET	pH=7.5 T=30ºC	[11]

R411Q	Active Site Residue	Very low activity	MHET	pH=7.5 T=30ºC	[11]
R411Q	Active Site Residue	Significantly decreases activity	MpNPT	pH=7.5 T=30ºC	[11]
R411Q	Active Site Residue	Small increase in activity	BHET	pH=7.5 T=30ºC	[11]
F415A	Active Site Residue	Slightly lower activity	MHET	pH=7.5 T=30ºC	[11]
F415A	Active Site Residue	Significantly decreases activity	MpNPT	pH=7.5 T=30ºC	[11]
F415H	Active Site Residue	Significantly increases activity	MHET	pH=7.5 T=30ºC	[11]
F415H	Active Site Residue	No difference	MpNPT	pH=7.5 T=30ºC	[11]
S416A	Substrate Interacting Residue	Significantly increases activity	MHET	pH=7.5 T=30ºC	[11]
S416A	Substrate Interacting Residue	Slightly lower activity	MpNPT	pH=7.5 T=30ºC	[11]
S416A	Substrate Interacting Residue	Significantly increases activity	BHET	pH=7.5 T=30ºC	[11]
S416G	Substrate Interacting Residue	Slightly lower activity	MHET	pH=7.5 T=30ºC	[11]
S416G	Substrate Interacting Residue	No difference	MpNPT	pH=7.5 T=30ºC	[11]
S416G	Substrate Interacting Residue	No difference	BHET	pH=7.5 T=30ºC	[11]
S419G	Substrate Interacting Residue	Slightly lower activity	MHET	pH=7.5 T=30ºC	[11]
S419G	Substrate Interacting Residue	Significantly increases activity	MpNPT	pH=7.5 T=30ºC	[11]
S419G	Substrate Interacting Residue	Significantly increases activity	BHET	pH=7.5 T=30ºC	[11]
F424A	Substrate Binding Hinderer	Significantly increases activity	BHET	pH=7.5 T=30ºC	[11]
F424N	Substrate Binding Hinderer	Significantly decreases activity	MHET	pH=7.5 T=30ºC	[11]
F424N	Substrate Binding Hinderer	Significantly decreases activity	MpNPT	pH=7.5 T=30ºC	[11]
F424N	Substrate Binding Hinderer	Significantly increases activity	BHET	pH=7.5 T=30ºC	[11]
F424Q	Substrate Binding Hinderer	Significantly decreases activity	MHET	pH=7.5 T=30ºC	[11]

F424Q	Substrate Binding Hinderer	Significantly decreases activity	MpNPT	pH=7.5 T=30ºC	[11]
F424Q	Substrate Binding Hinderer	Significantly increases activity	BHET	pH=7.5 T=30ºC	[11]
F424S	Substrate Binding Hinderer	Significantly increases activity	BHET	pH=7.5 T=30ºC	[11]
H488A	Substrate Interacting Residue	No difference	MHET	pH=7.5 T=30ºC	[11]
H488A	Substrate Interacting Residue	No difference	MpNPT	pH=7.5 T=30ºC	[11]
H488A	Active Site Residue	Significantly decreases activity	MHET	pH=7.5 T=30ºC	[11]
H488A	Active Site Residue	Significantly decreases activity	MpNPT	pH=7.5 T=30ºC	[11]
L254N/F424N	Double-mutant of previously described variants	Significantly decreases activity	MpNPT	pH=7.5 T=30ºC	[11]
L254N/F424N	Double-mutant of previously described variants	Small increase in activity	BHET	pH=7.5 T=30ºC	[11]
R411A/S416A	Double-mutant of previously described variants	Inactive	MHET	pH=7.5 T=30ºC	[11]
R411A/S416A	Double-mutant of previously described variants	Significantly decreases activity	MpNPT	pH=7.5 T=30ºC	[11]
R411A/S416A	Double-mutant of previously described variants	Significantly increases activity	BHET	pH=7.5 T=30ºC	[11]
R411A/S416G	Double-mutant of previously described variants	Inactive	MHET	pH=7.5 T=30ºC	[11]
R411A/S416G	Double-mutant of previously described variants	Significantly decreases activity	MpNPT	pH=7.5 T=30ºC	[11]
R411A/S416G	Double-mutant of previously described variants	Significantly increases activity	BHET	pH=7.5 T=30ºC	[11]
R411A/S419G	Double-mutant of previously described variants	Inactive	MHET	pH=7.5 T=30ºC	[11]
R411A/S419G	Double-mutant of previously described variants	Significantly decreases activity	MpNPT	pH=7.5 T=30ºC	[11]
R411A/S419G	Double-mutant of previously described variants	Significantly increases activity	BHET	pH=7.5 T=30ºC	[11]
R411Q/S416A	Double-mutant of previously described variants	Inactive	MHET	pH=7.5 T=30ºC	[11]
R411Q/S416A	Double-mutant of previously described variants	Significantly decreases activity	MpNPT	pH=7.5 T=30ºC	[11]
R411Q/S416A	Double-mutant of previously described variants	Significantly increases activity	BHET	pH=7.5 T=30ºC	[11]

R411Q/S416G	Double-mutant of previously described variants	Inactive	MHET	pH=7.5 T=30ºC	[11]
R411Q/S416G	Double-mutant of previously described variants	Significantly decreases activity	MpNPT	pH=7.5 T=30ºC	[11]
R411Q/S416G	Double-mutant of previously described variants	Significantly increases activity	BHET	pH=7.5 T=30ºC	[11]
R411A/S419G	Double-mutant of previously described variants	Inactive	MHET	pH=7.5 T=30ºC	[11]
R411A/S419G	Double-mutant of previously described variants	Significantly increases activity	MpNPT	pH=7.5 T=30ºC	[11]
R411A/S419G	Double-mutant of previously described variants	Significantly increases activity	BHET	pH=7.5 T=30ºC	[11]
F415H/F424N	Double-mutant of previously described variants	Significantly decreases activity	MpNPT	pH=7.5 T=30ºC	[11]
F415H/F424N	Double-mutant of previously described variants	Small increase in activity	BHET	pH=7.5 T=30ºC	[11]
F415H/F424N	Double-mutant of previously described variants	Significantly decreases activity	MpNPT	pH=7.5 T=30ºC	[11]
F415H/F424N	Double-mutant of previously described variants	Significantly increases activity	BHET	pH=7.5 T=30ºC	[11]
S416A/S419G	Double-mutant of previously described variants	Significantly decreases activity	MpNPT	pH=7.5 T=30ºC	[11]
S416A/S419G	Double-mutant of previously described variants	Significantly increases activity	BHET	pH=7.5 T=30ºC	[11]
S419G/F424N	Double-mutant of previously described variants	Significantly decreases activity	MpNPT	pH=7.5 T=30ºC	[11]
S419G/F424N	Double-mutant of previously described variants	Significantly increases activity	BHET	pH=7.5 T=30ºC	[11]
F424N/H467N	Double-mutant of previously described variants	Slightly lower activity	MpNPT	pH=7.5 T=30ºC	[11]
F424N/H467N	Double-mutant of previously described variants	Significantly increases activity	BHET	pH=7.5 T=30ºC	[11]
R411A/S416G/S419G	Triple-mutant of previously described variants	Significantly decreases activity	MpNPT	pH=7.5 T=30ºC	[11]
R411A/S416G/S419G	Triple-mutant of previously described variants	Significantly increases activity	BHET	pH=7.5 T=30ºC	[11]
R411A/S416G/S419G	Triple-mutant of previously described variants	Significantly decreases activity	MpNPT	pH=7.5 T=30ºC	[11]
R411A/S416G/S419G	Triple-mutant of previously described variants	Significantly increases activity	BHET	pH=7.5 T=30ºC	[11]
R411A/S416G/S419G	Triple-mutant of previously described variants	Significantly decreases activity	MpNPT	pH=7.5 T=30ºC	[11]

R411A/S416G/S419G	Triple-mutant of previously described variants	Significantly increases activity	BHET	pH=7.5 T=30ºC	[11]
S225A	Confirm role as catalytic triad residue	Complete loss of activity	MHET	pH=8 T=30ºC	[11]
S131G	Substrate accommodation residue	Km= 184.10 μM ; Significantly higher than WT	MHET	pH=8 T=30ºC	[11]
E226T	Oxyanion hole	Significantly decreases activity	MHET	pH=8 T=30ºC	[11]
F495I	Active Site Residue	Significantly decreases activity; Kcat= 1.3 ± 0.7 s-1	MHET	pH=8 T=30ºC	[11]
Lidless MHETase	Removed Lid domain	Very low activity	MHET	pH=8 T=30ºC	[11]
Lidless MHETase C224W/C529S	Removed Lid domain and disulfide bond	Very low activity	MHET	pH=8 T=30ºC	[11]
Lidless MHETase C224H/C529F	Removed Lid domain and disulfide bond	Very low activity	MHET	pH=8 T=30ºC	[11]
MHETase with 7 disulfides	Additional 2 disulfide bonds	Very low activity	MHET	pH=8 T=30ºC	[11]

Table S3: Mutagenesis assays with *Pa*PETase

Mutation	Rationale	Assay Results	Substrate	Assay Conditions	REF
G254S	Replace with PETase residues	Diminished Activity	*p*NPB	pH=7.4 T= 30 ºC	[12]
S256N	Replace with PETase residues	Diminished Activity	*p*NPB	pH=7.4 T= 30 ºC	[12]
I257S	Replace with PETase residues	Diminished Activity	*p*NPB	pH=7.4 T= 30 ºC	[12]
Y258N	Replace with PETase residues	Diminished Activity	*p*NPB	pH=7.4 T= 30 ºC	[12]
N259Q	Replace with PETase residues	Diminished Activity	*p*NPB	pH=7.4 T= 30 ºC	[12]
Y250S	Replace with PETase residues	Increased Activity	*p*NPB	pH=7.4 T= 30 ºC	[12]
G254S, S256N, I257S, Y258N, N259Q	Replace with PETase residues	Diminished Activity	*p*NPB	pH=7.4 T= 30 ºC	[12]
G254S	Replace with PETase residues	Diminished Activity	BHET	pH=7.4 T= 30 ºC, 24 h	[12]
S256N	Replace with PETase residues	Diminished Activity	BHET	pH=7.4 T= 30 ºC, 24 h	[12]
I257S	Replace with PETase residues	Diminished Activity	BHET	pH=7.4 T= 30 ºC, 24 h	[12]
Y258N	Replace with PETase residues	Diminished Activity	BHET	pH=7.4 T= 30 ºC, 24 h	[12]
N259Q	Replace with PETase residues	Diminished Activity	BHET	pH=7.4 T= 30 ºC, 24 h	[12]
Y250S	Replace with PETase residues	Increased Activity	BHET	pH=7.4 T= 30 ºC, 24 h	[12]
G254S, S256N, I257S, Y258N, N259Q	Replace with PETase residues	Diminished Activity	BHET	pH=7.4 T= 30 ºC, 24 h	[12]
G254S	Replace with PETase residues	Diminished Activity	Amorphous PET	pH=7.4 T= 30 ºC, 48 h	[12]
S256N	Replace with PETase residues	Diminished Activity	Amorphous PET	pH=7.4 T= 30 ºC, 48 h	[12]
I257S	Replace with PETase residues	Diminished Activity	Amorphous PET	pH=7.4 T= 30 ºC, 48 h	[12]
Y258N	Replace with PETase residues	Diminished Activity	Amorphous PET	pH=7.4 T= 30 ºC, 48 h	[12]
N259Q	Replace with PETase residues	Diminished Activity	Amorphous PET	pH=7.4 T= 30 ºC, 48 h	[12]
Y250S	Replace with PETase residues	Increased Activity	Amorphous PET	pH=7.4 T= 30 ºC, 48 h	[12]
G254S, S256N, I257S, Y258N, N259Q	Replace with PETase residues	Diminished Activity	Amorphous PET	pH=7.4 T= 30 ºC, 48 h	[12]
G254S	Replace with PETase residues	No activity	Commercial PET	pH=7.4 T= 30 ºC, 48 h	[12]
S256N	Replace with PETase residues	No activity	Commercial PET	pH=7.4 T= 30 ºC, 48 h	[12]
I257S	Replace with PETase residues	No activity	Commercial PET	pH=7.4 T= 30 ºC, 48 h	[12]
Y258N	Replace with PETase residues	No activity	Commercial PET	pH=7.4 T= 30 ºC, 48 h	[12]

N259Q	Replace with PETase residues	Increased Activity	Commercial PET	pH=7.4 T= 30 ºC, 48 h	[12]
Y250S	Replace with PETase residues	Increased Activity	Commercial PET	pH=7.4 T= 30 ºC, 48 h	[12]
G254S, S256N, I257S, Y258N, N259Q	Replace with PETase residues	No activity	Commercial PET	pH=7.4 T= 30 ºC, 48 h	[12]

Table S4: Mutagenesis assays with LCC

Mutation	Rationale	Assay Results	Substrate	Assay Conditions	REF
C275A/C292A	Disrupting disulfide bridge	Tm 15 ºC lower than WT	PET film	pH = 8.0	[13]
F243I	Binding site residues	27 % increase in activity	PET film	pH = 8.0	[14]
F243W	Binding site residues	17 % increase in activity	PET film	pH = 8.0	[14]
T96M	Binding site residues	Similar acitivy and higher Tm than WT	PET film	pH = 8.0	[14]
Y127G	Binding site residues	Similar acitivy and higher Tm than WT	PET film	pH = 8.0	[14]
N246D	Binding site residues	Similar acitivy and higher Tm than WT	PET film	pH = 8.0	[14]
N246M	Binding site residues	Similar acitivy and higher Tm than WT	PET film	pH = 8.0	[14]
D238C/S283C	Additional disulfide bond	Tm increased by 9.8 ºC compared with WT; 28 % activity loss	PET film	pH = 8.0	[14]
F243I/D238C/S283C	Combination of tested variants	Similar acitivy and higher Tm than WT	PET film	pH = 8.0	[14]
F243W/D238C/S283C	Combination of tested variants	Similar acitivy and higher Tm than WT	PET film	pH = 8.0	[14]
F243I/D238C/S283C/Y127G	Combination of tested variants	Similar acitivy and higher Tm than WT	PET film	pH = 8.0	[14]
F243I/D238C/S283C/N246M	Combination of tested variants	Similar acitivy and higher Tm than WT	PET film	pH = 8.0	[14]
F243W/D238C/S283C/Y127G	Combination of tested variants	Similar acitivy and higher Tm than WT	PET film	pH = 8.0	[14]
F243W/D238C/S283C/N246M	Combination of tested variants	Similar acitivy and higher Tm than WT	PET film	pH = 8.0	[14]

Table S5: Mutagenesis assays with *Tf*HCut

Mutation	Rationale	Assay Results	Substrate	Assay Conditions	REF
I218A	Active site enlargement	two-fold activity increase compared to WT	PET fabric	60 ºC pH 7.5	[15]
Q132A/T101A	Increase active site hydrophobicity	two-fold activity increase compared to WT	PET fabric	60 ºC pH 7.5	[15]
Y100A	Residues near catalytic triad	Activity increase	*p*NPB	pH 8.0	[16]
T101A	Residues near catalytic triad	Activity decrease	*p*NPB	pH 8.0	[16]
H169A	Residues near catalytic triad	Activity decrease	*p*NPB	pH 8.0	[16]
M171A	Residues near catalytic triad	Activity decrease	*p*NPB	pH 8.0	[16]
T247A	Residues near catalytic triad	Activity increase	*p*NPB	pH 8.0	[16]
F249A	Residues near catalytic triad	Activity increase	*p*NPB	pH 8.0	[16]
S106A	Residues near substrate binding site	Activity decrease	*p*NPB	pH 8.0	[16]
L130A	Residues near substrate binding site	Activity increase	*p*NPB	pH 8.0	[16]
Q132A	Residues near substrate binding site	Activity decrease	*p*NPB	pH 8.0	[16]
N252A	Residues near substrate binding site	Activity decrease	*p*NPB	pH 8.0	[16]
I253A	Residues near substrate binding site	Activity increase	*p*NPB	pH 8.0	[16]

Table S6: Mutagenesis assays with *Sv*Cut190.

Mutations	Rationale	Assay result	Substrate	Assay condition	REF
S226P/R228S/S176	Substrate recognition mechanism analyses	Inactive	Et- succinate	-	[17]
S226P/R228S/S176	Substrate recognition mechanism analyses	Inactive	Et-adipate	-	[17]
S226P/R228S/Q138A/D250C-296C/Q123H/N202H/S176A	Metal binding in the inactive form	Inactive	-	70 ºC, pH 7.0, 24%glycerol	[18]
/S226P/R228S/S176A with deletion of the three C-terminal residues	Metal binding in the inactive form	Inactive	-	37 ºC, 7.0 pH, 300 mM CaCl2	[19]
S226P	Increased thermostability in other enzyme	Increased thermostability by 3.7 ºC	-	37, ºC pH 7.0, [Ca2+] free	[20]
S226P	Increased thermostability in other enzyme	Increased thermostability in presence of Ca2+ by 4.9 ºC	-	37, ºC pH 7.0, 300 mM [Ca2+]	[20]
S226P/R228S	Adding a variant that potentially influences the reaction	Kcat=7.1 s-1; Km= 0.68 mM	PNPB	37 ºC, pH 7.0, 300 mM [Ca2+]	[20]
S226P/R228S	Adding a variant that potentially influences the reaction	Kcat=8.0 s-1; Km= 0.04 mM	PBSA	37 ºC, pH 7.0, 300 mM [Ca2+]	[20]
S226P/R228S	Adding a variant that potentially influences the reaction	Kcat 8.0 s-1; Km= 10.8 mM	PBSA Unit	37, ºC pH 7.0, 300 mM [Ca2+]	[20]
S226P/R228S	Influence activity	Increased thermostability in presence of Ca2+ by 6.2 ºC	-	37, ºC pH 7.0, 300 mM [Ca2+]	[20]
S226P/R228S/T262K	Adding a common residue in homologous cutinases	Diminishes thermostability when compared to the double mutant	-	37, ºC pH 7.0, 300 mM [Ca2+]	[20]
S226P/R228S/W201A	Vicinity of the oxyanion hole	Kcat= 2.9 ± 0.07 s-1; Km= 0.65 ± 0.01 mM	PBSA	37 ºC, pH 8.2 , 2.5 mM [Ca2+]	[21]
S226P/R228S/F106A	Oxyanion hole reisude	Kcat= 0.65 ± 0.03 s-1; Km= 0.016 ± 0.001 mM	PBSA	37 ºC, pH 8.2 , 2.5 mM [Ca2+]	[21]

S226P/R228S/F106Y	Oxyanion hole reisude	Kcat= 20 ± 0.05 s-1; Km= 0.080 ± 0.005mM	PBSA	37 ºC, pH 8.2 , 2.5 mM [Ca2+]	[21]
S226P/R228S/M177A	Oxyanion hole reisude	No activity	PBSA	37 ºC, pH 8.2 , 2.5 mM [Ca2+]	[21]
S226P/R228S/Q138A	Vicinity of the oxyanion hole	Kcat= 65 ± 0.4 s-1; Km= 0.048 ± 0.001 mM	PBSA	37 ºC, pH 8.2 , 2.5 mM [Ca2+]	[21]
S226P/R228S/T107A	Vicinity of the oxyanion hole	Kcat= 8.7 ± 0.05 s-1; Km= 0.036 ± 0.001 mM	PBSA	37 ºC, pH 8.2 , 2.5 mM [Ca2+]	[21]
S226P/R228S/Q138D	Vicinity of the oxyanion hole	Kcat= 61 ± 0.3 s-1; Km= 0.21 ± 0.001 mM	PBSA	37 ºC, pH 8.2 , 2.5 mM [Ca2+]	[21]
S226P/R228S/I224A	Active site residue	Kcat= 150 ± 0.2 s-1; Km= 0.15 ± 0.003 mM	PBSA	37 ºC, pH 8.2 , 2.5 mM [Ca2+]	[21]
S226P/R228S/I224/Q138A	Active site and vicinity of the oxyanion hole residues	Kcat= 150 ± 0.2 s-1; Km= 0.24 ± 0.002 mM	PBSA	37 ºC, pH 8.2 , 2.5 mM [Ca2+]	[21]
S226P/R228S/H175A	Ca2+ binding-site residue	No activity	PBSA	37 ºC, pH 8.2 , 2.5 mM [Ca2+]	[21]
S226P/R228S/N258A	Ca2+ binding-site residue	Kcat= 26 ± 0.01 s-1; Km= 0.065 ± 0.001 mM	PBSA	37 ºC, pH 8.2 , 2.5 mM [Ca2+]	[21]
S226P/R228S /S112A	Ca2+ binding-site residue	Kcat= 30 ± 0.1 s-1; Km= 0.071 ± 0.001 mM	PBSA	37 ºC, pH 8.2 , 2.5 mM [Ca2+]	[21]
S226P/R228S/N258A/I224A	Active site and vicinity of the oxyanion hole residues	Kcat= 30 ± 0.05 s-1; Km= 0.15 ± 0.001 mM	PBSA	37 ºC, pH 8.2 , 2.5 mM [Ca2+]	[21]
S226P/R228S with deletion of the three C-terminal residues	C-terminal residues deletion	Kcat= 100 s-1; Km= 0.282 mM	PBSA	37 ºC, pH 7.0 , 300 mM CaCl2	[19]
S226P/R228S/Q138A	Vicinity of the oxyanion hole	Kcat= 64 s-1 ; Km= 0.048 mM ; Tm= 55.9 ºC	PBSA	37 ºC, pH 8.2 , [Ca2+] free	[22]
S226P/R228S/Q138A	Vicinity of the oxyanion hole	Kcat= 64 s-1 ; Km= 0.048 mM ; Tm= N/A ºC	PBSA	37 ºC, pH 8.2, 2.5 mM [Ca2+]	[22]
S226P/R228S/78S	Ca2+ binding-site residue introduction	Kcat= 151 s-1 ; Km= 0.12 mM ; Tm= 55.8 ºC	PBSA	37 ºC, pH 8.2 , [Ca2+] free	[22]
S226P/R228S/78S	Ca2+ binding-site residue introduction	Kcat= 151 s-1 ; Km= 0.12 mM ; Tm= 66.7 ºC	PBSA	37 ºC, pH 8.2, 2.5 mM [Ca2+]	[22]

S226P/R228S/D250C-296C	Disulfide bond introduction	Kcat= 50 s-1 ; Km= 0.15 mM ; Tm= 79.0 ºC	PBSA	37 ºC, pH 8.2 , [Ca2+] free	[22]
S226P/R228S/D250C-296C	Disulfide bond introduction	Kcat= 50 s-1 ; Km= 0.15 mM ; Tm= 78.9 ºC	PBSA	37 ºC, pH 8.2, 2.5 mM [Ca2+]	[22]
S226P/R228S/Q138A/D250C-296C	Ca2+ binding-site reisude and disulfide bond introduction	Kcat= 150 s-1 ; Km= 0.39 mM ; Tm= 79.2 ºC	PBSA	37 ºC, pH 8.2 , [Ca2+] free	[22]
S226P/R228S/Q138A/D250C-296C	Ca2+ binding-site reisude and disulfide bond introduction	Kcat= 150 s-1 ; Km= 0.39 mM ; Tm= 77.9 ºC	PBSA	37 ºC, pH 8.2, 2.5 mM [Ca2+]	[22]
S226P/R228S/Q138A/D250C-296C/E220R	Ca2+ binding-site reisude and disulfide bond introduction	Kcat= 150 s-1 ; Km= 0.48 mM ; Tm= 78.3 ºC	PBSA	37 ºC, pH 8.2 , [Ca2+] free	[22]
S226P/R228S/Q138A/D250C-296C/E220R	Ca2+ binding-site reisude and disulfide bond introduction	Kcat= 150 s-1 ; Km= 0.48 mM ; Tm= 72.3 ºC	PBSA	37 ºC, pH 8.2, 2.5 mM [Ca2+]	[22]
S226P/R228S/Q138A/D250C-296C/E220R-G251D	Ca2+ binding-site reisude and disulfide bond introduction	Kcat= 75 s-1 ; Km= 0.18 mM ; Tm= 75.8 ºC	PBSA	37 ºC, pH 8.2 , [Ca2+] free	[22]
S226P/R228S/Q138A/D250C-296C/E220R-G251D	Ca2+ binding-site reisude and disulfide bond introduction	Kcat= 75 s-1 ; Km= 0.18 mM ; Tm= 75.3 ºC	PBSA	37 ºC, pH 8.2, 2.5 mM [Ca2+]	[22]
S226P/R228S/Q138A/D250C-296C/Q123H/N202H	Ca2+ binding-site reisude and disulfide bond introduction and surface modification	Kcat= 100 s-1 ; Km= 0.32 mM ; Tm= 85.7 ºC	PBSA	37 ºC, pH 8.2 , [Ca2+] free	[22]
S226P/R228S/Q138A/D250C-296C/Q123H/N202H	Ca2+ binding-site reisude and disulfide bond introduction and surface modification	Kcat= 100 s-1 ; Km= 0.32 mM ; Tm= 82.6 ºC	PBSA	37 ºC, pH 8.2, 2.5 mM [Ca2+]	[22]
S226P/R228S/E184R	Ca2+ binding-site residue	Kcat= 60 s-1 ; Km= 0.17 mM ; Tm= 53.9 ºC	PBSA	37 ºC, pH 8.2 , [Ca2+] free	[22]
S226P/R228S/E184R	Surface modification	Kcat= 60 s-1 ; Km= 0.17 mM ; Tm= 64.1 ºC	PBSA	37 ºC, pH 8.2, 2.5 mM [Ca2+]	[22]
S226P/R228S/Q110E	Surface modification	Kcat= 27 s-1 ; Km= 0.070 mM ; Tm= 59. ºC	PBSA	37 ºC, pH 8.2 , [Ca2+] free	[22]
S226P/R228S/N133D	Surface modification	Kcat= 25 s-1 ; Km= 0.079 mM ; Tm= 60.4 ºC	PBSA	37 ºC, pH 8.2 , [Ca2+] free	[22]
S226P/R228S/Q141E	Surface modification	Kcat= 50 s-1 ; Km= 0.11 mM ; Tm= 60.7 ºC	PBSA	37 ºC, pH 8.2 , [Ca2+] free	[22]

S226P/R228S/Q138A/Q141E	Ca2+ binding-site reisude and surface modification	Kcat= 19 s-1 ; Km= 0.045 mM ; Tm= 57.9 ºC	PBSA	37 ºC, pH 8.2 , [Ca2+] free	[22]
S226P/R228S/N168D	Surface modification	Kcat= 30 s-1 ; Km= 0.091 mM ; Tm= 56.0 ºC	PBSA	37 ºC, pH 8.2 , [Ca2+] free	[22]
S226P/R228S/N168A	Surface modification	Kcat= 43 s-1 ; Km= 0.10 mM ; Tm= 56.0 ºC	PBSA	37 ºC, pH 8.2 , [Ca2+] free	[22]
S226P/R228S/Q209E	Surface modification	Kcat= 75 s-1 ; Km= 0.20 mM ; Tm= 58.6 ºC	PBSA	37 ºC, pH 8.2 , [Ca2+] free	[22]
S226P/R228S/Q123H	Surface modification	Kcat= 61 s-1 ; Km= 0.14 mM ; Tm= 58.6 ºC	PBSA	37 ºC, pH 8.2 , [Ca2+] free	[22]
S226P/R228S/N202H	Surface modification	Kcat= 60 s-1 ; Km= 0.16 mM ; Tm= 62.4 ºC	PBSA	37 ºC, pH 8.2 , [Ca2+] free	[22]
S226P/R228S	Previously studied double mutant	Degrade rate= 80.2 ± 4.55 ; Amount of total products= 12.5 ± 0.72 mM	Microfiber PET	65 ºC, pH 8.5, 2.5 µmol CaCl2, 24% glycerol, 2 nmol enzyme and 2 mg microfiber PET	[22]
S226P/R228S/Q138A	Vicinity of the oxyanion hole	Degrade rate= 104 ± 7.00 ; Amount of total products= 16.2 ± 1.1 mM	Microfiber PET	65 ºC, pH 8.5 , 2.5 µmol CaCl2, 24% glycerol, 2 nmol enzyme and 2 mg microfiber PET	[22]
S226P/R228S/I224A	Active site residue	Degrade rate= 44.9 ± 5.60 ; Amount of total products= 6.96 ± 0.87 mM	Microfiber PET	65 ºC, pH 8.5 , 2.5 µmol CaCl2, 24% glycerol, 2 nmol enzyme and 2 mg microfiber PET	[22]
S226P/R228S	Previously studied double mutant	Degradation rate products= 4.53 ± 0.29 µmol/cm2	APEXA 4027TM	50 ºC, pH 8.2-8.5, 2.5 µmol CaCl2, 24% glycerol, 2 µM Cut190 S226P/R228S	[22]
S226P/R228S	Previously studied double mutant	Degradation rate products= 46.9 ± 1.6 µmol/cm2	PET-S	60 ºC, pH 8.2-8.5, 2.5 µmol CaCl2, 24% glycerol, 2 µM Cut190 S226P/R228S	[22]
S226P/R228S	Previously studied double mutant	Degradation rate products= 12.3 ± 1.2 µmol/cm2	PET-GF	65 ºC, pH 8.2-8.5, 2.5 µmol CaCl2, 24% glycerol, 2 µM Cut190 S226P/R228S	[22]
S226P/R228S/Q138A/D250C-E296C	Ca2+ binding-site reisude and disulfide bond introduction	Degradation rate products= 19.2 ± 1.9 µmol/cm2	PET-GF	70 ºC, pH 8.2-8.5, 2.5 µmol CaCl2, 24% glycerol, 2 µM Cut190 S226P/R228S	[22]

S226P/R228S/Q138A/D250C-E296C/E-220R-G251D	Ca2+ binding-site reisude and disulfide bond introduction	Degradation rate products= 11.8 ± 1.8 μmol/cm2	PET-GF	70 ºC, pH 8.2-8.5, 2.5 μmol CaCl2, 24% glycerol, 2 μM Cut190 S226P/R228S	[22]
S226P/R228S/Q138A/D250C-E296C/E-220R-G251D/Q123H/N202H	Ca2+ binding-site reisude and disulfide bond introduction and surface modification	Degradation rate products= 13.9 ± 2.3 μmol/cm2	PET-GF	70 ºC, pH 8.2-8.5, 2.5 μmol CaCl2, 24% glycerol, 2 μM Cut190 S226P/R228S	[22]
S226P/R228S/Q138A/D250C-E296C//Q123H/N202H	Ca2+ binding-site reisude and disulfide bond introduction and surface modification	Degradation rate products= 28.6 ± 2.3 μmol/cm2	PET-GF	70 ºC, pH 8.2-8.5, 2.5 μmol CaCl2, 24% glycerol, 2 μM Cut190 S226P/R228S	[22]
S226P/R228S/Q138A/D250C-296C/Q123H/N202H	Ca2+ binding-site reisude, disulfide bond introduction and surface modification	Tm= 82.1 ºC	PBSA	37 ºC, pH 8.2, [Ca2+] free	[18]
S226P/R228S/Q138A/D250C-296C/Q123H/N202H	Ca2+ binding-site reisude, disulfide bond introduction and surface modification	Kcat= 26.7 s-1 ; Km= 0.079 mM ; Tm= 83.3 ºC	PBSA	37 ºC, pH 8.2 , 2.5 mM [Ca2+]	[18]
S226P/R228S/Q138A/D250C-296C/Q123H/N202H	Ca2+ binding-site reisude, disulfide bond introduction and surface modification	Kcat= 39.2 s-1 ; Km= 0.171 mM ; Tm= 84.8 ºC	PBSA	37 ºC, pH 8.2, 25 mM [Ca2+]	[18]

Table S7: Mutagenesis assays with *Tc*Cut2

Mutations	Rationale	Assay result	Substrate	Assay condition	REF
S131A	Catalytic triad residue substitution	Inactive	PBA	20 ºC , pH 7.0	[23]
R19S	Substitution by the residue located in the same position in Thc_Cut1	Kcat= 36 s-1 ; Km= 1.2 ± 0.1 mM	pNPA	25 ºC , pH 7.0	[24]
R19S	Substitution by the residue located in the same position in Thc_Cut1	Kcat= 80 s-1 ; Km= 2.2 ± 0.4 mM	pNPB	25 ºC , pH 7.0	[24]
R29D	Substitution by the residue located in the same position in Thc_Cut1	Kcat= 207 s-1 ; Km= 1.3 ± 0.4 mM	pNPA	25 ºC , pH 7.0	[24]
R29D	Substitution by the residue located in the same position in Thc_Cut1	Kcat= 191 s-1 ; Km= 2.0 ± 0.4 mM	pNPB	25 ºC , pH 7.0	[24]
A30V	Substitution by the residue located in the same position in Thc_Cut1	Kcat= 260 s-1 ; Km= 1.7 ± 0.2 mM	pNPA	25 ºC , pH 7.0	[24]
A30V	Substitution by the residue located in the same position in Thc_Cut1	Kcat= 183 s-1 ; Km= 1.1 ± 0.3 mM	pNPB	25 ºC , pH 7.0	[24]
Q65E	Substitution by the residue located in the same position in Thc_Cut1	Kcat= 7 s-1 ; Km= 1.5 ± 0.2 mM	pNPA	25 ºC , pH 7.0	[24]
Q65E	Substitution by the residue located in the same position in Thc_Cut1	Kcat= 15 s-1 ; Km= 2.6 ± 0.8 mM	pNPB	25 ºC , pH 7.0	[24]
L83A	Substitution by the residue located in the same position in Thc_Cut1	Kcat= 25 s-1 ; Km= 1.9 ± 0.1 mM	pNPA	25 ºC , pH 7.0	[24]
L83A	Substitution by the residue located in the same position in Thc_Cut1	Kcat= 32 s-1 ; Km= 2.1 ± 0.6 mM	pNPB	25 ºC , pH 7.0	[24]
R187K	Substitution by the residue located in the same position in Thc_Cut1	Kcat= 35 s-1 ; Km= 0.8 ± 0.1 mM	pNPA	25 ºC , pH 7.0	[24]
R187K	Substitution by the residue located in the same position in Thc_Cut1	Kcat= 79 s-1 ; Km= 4.0 ± 0.5 mM	pNPB	25 ºC , pH 7.0	[24]
R29D/A30V	Improve previously discovered variant	Kcat= 100 s-1 ; Km= 1.3 ± 0.3 mM	pNPA	25 ºC , pH 7.0	[24]
R29D/A30V	Improve previously discovered variant	Kcat= 173 s-1 ; Km= 1.0 ± 0.3 mM	pNPB	25 ºC , pH 7.0	[24]
R19S/R29D/A30V	Lower activity than the double mutant	Kcat= 76 s-1 ; Km= 1.2 ± 0.1 mM	pNPA	25 ºC , pH 7.0	[24]
R19S/R29D/A30V	Lower activity than the double mutant	Kcat= 136 s-1 ; Km= 1.4 ± 0.4 mM	pNPB	25 ºC , pH 7.0	[24]

Table S8: Mutagenesis assays with *Ta*Est1.

Mutations	Rationale	Assay result	Substrate	Assay condition	REF
A68V/T253P	Increase thermal stability	Activity and Tm increase	3PET	pH 7.0 50 ºC	[25]
A68V	Increase thermal stability	Activity increase	3PET	pH 7.0 50 ºC	[25]
A68V/M259K	Increase thermal stability	Similar activity to A68V/T253P but lowe Tm	3PET	pH 7.0 50 ºC	[25]
A68V/T253P/M259K	Increase thermal stability	Similar activity to A68V/T253P but lowe Tm	3PET	pH 7.0 50 ºC	[25]

Table S9: Mutagenesis assays with *Tl*Lip

Mutations	Rationale	Assay result	Substrate	Assay condition	REF
E109A	Important residue for stabilization	Significantly lower activity	Tributyrin	25 ºC, pH 7.5	[26]
E109A	Important residue for stabilization	Significantly lower activity	Trioctanoin	25 ºC, pH 7.5	[26]
W111L	Important residue for acylation	Significantly lower activity	Tributyrin	25 ºC, pH 7.5	[26]
W111L	Important residue for acylation	Significantly lower activity	Trioctanoin	25 ºC, pH 7.5	[26]
W111F	Important residue for acylation	Significantly lower activity	Tributyrin	25 ºC, pH 7.5	[26]
W111F	Important residue for acylation	Significantly lower activity	Trioctanoin	25 ºC, pH 7.5	[26]
W111E	Important residue for acylation	Significantly lower activity	Tributyrin	25 ºC, pH 7.5	[26]
W111E	Important residue for acylation	Significantly lower activity	Trioctanoin	25 ºC, pH 7.5	[26]
W111G	Important residue for acylation	Significantly lower activity	Tributyrin	25 ºC, pH 7.5	[26]
W111G	Important residue for acylation	Significantly lower activity	Trioctanoin	25 ºC, pH 7.5	[26]
I108C/I277C	Introducing disulfind bond	Significantly lower specific activity in the absence of TCEP but higher with 5 mM of TCEP	p-NPB	27 ºC, pH 7.5, 10 mM [Ca2+], 0-5 mM TCEP	[27]
I108C/I277C	Introducing disulfind bond	Significantly lower specific activity in the absence of TCEP but higher with 10 mM of TCEP	p-NPDecanoate	27 ºC, pH 7.5, 10 mM [Ca2+], 0-10 mM TCEP	[27]

Table S10: Mutagenesis assays with *Tf*Ca

Mutations	Rationale	Assay result	Substrate	Assay condition	REF
E184Q	Important residue for enzymatic activity	Signifincatly lower activity	p-NPB	pH 7.5, 10 μL ethanol	[28]
E319D	Important residue for enzymatic activity	Signifincatly lower activity	p-NPB	pH 7.5, 10 μL ethanol	[28]
E184Q/E319D	Important residues for enzymatic activity	Signifincatly lower activity	p-NPB	pH 7.5, 10 μL ethanol	[28]
E184H/A186M	Important residues for enzymatic activity	Signifincatly lower activity	p-NPB	pH 7.5, 10 μL ethanol	[28]
E184H/A186M/E319D	Important residues for enzymatic activity	Signifincatly lower activity	p-NPB	pH 7.5, 10 μL ethanol	[28]

[1] Han, X.; Liu, W.; Huang, J.-W.; Ma, J.; Zheng, Y.; Ko, T.-P.; Xu, L.; Cheng, Y.-S.; Chen, C.-C.; Guo, R.-T. Structural Insight into Catalytic Mechanism of PET Hydrolase. *Nat. Commun.*, **2017**, *8*, 2106.

[2] Joo, S.; Cho, I.J.; Seo, H.; Son, H.F.; Sagong, H.-Y.; Shin, T.J.; Choi, S.Y.; Lee, S.Y.; Kim, K.-J. Structural Insight into Molecular Mechanism of Poly(Ethylene Terephthalate) Degradation. *Nat. Commun.*, **2018**, *9*, 382.

[3] Austin, H.P.; Allen, M.D.; Donohoe, B.S.; Rorrer, N.A.; Kearns, F.L.; Silveira, R.L.; Pollard, B.C.; Dominick, G.; Duman, R.; El Omari, K.; Mykhaylyk, V.; Wagner, A.; Michener, W.E.; Amore, A.; Skaf, M.S.; Crowley, M.F.; Thorne, A.W.; Johnson, C.W.; Woodcock, H.L.; McGeehan, J.E.; Beckham, G.T. Characterization and Engineering of a Plastic-Degrading Aromatic Polyesterase. *Proc. Natl. Acad. Sci. U. S. A.*, **2018**, *115*, E4350–E4357.

[4] Liu, B.; He, L.; Wang, L.; Li, T.; Li, C.; Liu, H.; Luo, Y.; Bao, R. Protein Crystallography and Site-Direct Mutagenesis Analysis of the Poly(Ethylene Terephthalate) Hydrolase PETase from Ideonella Sakaiensis. *Chembiochem*, **2018**, *19*, 1471–1475.

[5] Liu, C.; Shi, C.; Zhu, S.; Wei, R.; Yin, C.-C. Structural and Functional Characterization of Polyethylene Terephthalate Hydrolase from Ideonella Sakaiensis. *Biochem. Biophys. Res. Commun.*, **2019**, *508*, 289–294.

[6] Son, H.F.; Cho, I.J.; Joo, S.; Seo, H.; Sagong, H.-Y.; Choi, S.Y.; Lee, S.Y.; Kim, K.-J. Rational Protein Engineering of Thermo-Stable PETase from Ideonella Sakaiensis for Highly Efficient PET Degradation. *ACS Catal.*, **2019**, *9*, 3519–3526.

[7] Cui, Y.; Chen, Y.; Liu, X.; Dong, S.; Tian, Y.; Qiao, Y.; Mitra, R.; Han, J.; Li, C.; Han, X.; Liu, W.; Chen, Q.; Du, W.; Tang, S.; Xiang, H.; Liu, H.; Wu, B. Computational Redesign of a PETase for Plastic Biodegradation by the GRAPE Strategy. *bioRxiv*, **2019**.

[8] Son, H.F.; Joo, S.; Seo, H.; Sagong, H.Y.; Lee, S.H.; Hong, H.; Kim, K.J. Structural Bioinformatics-Based Protein Engineering of Thermo-Stable PETase from Ideonella Sakaiensis. *Enzyme Microb. Technol.*, **2020**, *141*, 109656.

[9] Ma, Y.; Yao, M.; Li, B.; Ding, M.; He, B.; Chen, S.; Zhou, X.; Yuan, Y. Enhanced Poly(Ethylene Terephthalate) Hydrolase Activity by Protein Engineering. *Engineering*, **2018**, *4*, 888–893.

[10] Sagong, H.-Y.; Seo, H.; Kim, T.; Son, H.F.; Joo, S.; Lee, S.H.; Kim, S.; Woo, J.-S.; Hwang, S.Y.; Kim, K.-J. Decomposition of the PET Film by MHETase Using Exo-PETase Function. *ACS Catal.*, **2020**, *10*, 4805–4812.

[11] Palm, G.J.; Reisky, L.; Böttcher, D.; Müller, H.; Michels, E.A.P.; Walczak, M.C.; Berndt, L.; Weiss, M.S.; Bornscheuer, U.T.; Weber, G. Structure of the Plastic-Degrading Ideonella Sakaiensis MHETase Bound to a Substrate. *Nat. Commun.*, **2019**, *10*, 1717.

[12] Bollinger, A.; Thies, S.; Knieps-Grünhagen, E.; Gertzen, C.; Kobus, S.; Höppner, A.; Ferrer, M.; Gohlke, H.; Smits, S.H.J.; Jaeger, K.E. A Novel Polyester Hydrolase From the Marine Bacterium Pseudomonas Aestusnigri – Structural and Functional Insights. *Front. Microbiol.*, **2020**, *11*, 1–16.

[13] Sulaiman, S.; You, D.-J.; Kanaya, E.; Koga, Y.; Kanaya, S. Crystal Structure and Thermodynamic and Kinetic Stability of Metagenome-Derived LC-Cutinase. *Biochemistry*, **2014**, *53*, 1858–1869.

[14] Tournier, V.; Topham, C.M.; Gilles, A.; David, B.; Folgoas, C.; Moya-Leclair, E.; Kamionka, E.; Desrousseaux, M.-L.; Texier, H.; Gavalda, S.; Cot, M.; Guémard, E.; Dalibey, M.; Nomme, J.;

Cioci, G.; Barbe, S.; Chateau, M.; André, I.; Duquesne, S.; Marty, A. An Engineered PET Depolymerase to Break down and Recycle Plastic Bottles. *Nature*, **2020**, *580*, 216–219.

[15] Silva, C.; Da, S.; Silva, N.; Matamá, T.; Araújo, R.; Martins, M.; Chen, S.; Chen, J.; Wu, J.; Casal, M.; Cavaco-Paulo, A. Engineered Thermobifida Fusca Cutinase with Increased Activity on Polyester Substrates. *Biotechnol. J.*, **2011**, *6*, 1230–1239.

[16] Dong, Q.; Yuan, S.; Wu, L.; Su, L.; Zhao, Q.; Wu, J.; Huang, W.; Zhou, J. Structure-Guided Engineering of a Thermobifida Fusca Cutinase for Enhanced Hydrolysis on Natural Polyester Substrate. *Bioresour. Bioprocess.*, **2020**, *7*, 37.

[17] Numoto, N.; Kamiya, N.; Bekker, G.-J.; Yamagami, Y.; Inaba, S.; Ishii, K.; Uchiyama, S.; Kawai, F.; Ito, N.; Oda, M. Structural Dynamics of the PET-Degrading Cutinase-like Enzyme from Saccharomonospora Viridis AHK190 in Substrate-Bound States Elucidates the Ca2+-Driven Catalytic Cycle. *Biochemistry*, **2018**, *57*, 5289–5300.

[18] Emori, M.; Numoto, N.; Senga, A.; Bekker, G.-J.; Kamiya, N.; Kobayashi, Y.; Ito, N.; Kawai, F.; Oda, M. Structural Basis of Mutants of PET-Degrading Enzyme from Saccharomonospora Viridis AHK190 with High Activity and Thermal Stability. *Proteins Struct. Funct. Bioinforma.*, **2021**, *89*, 502–511.

[19] Senga, A.; Numoto, N.; Yamashita, M.; Iida, A.; Ito, N.; Kawai, F.; Oda, M. Multiple Structural States of Ca2+-Regulated PET Hydrolase, Cut190, and Its Correlation with Activity and Stability. *J. Biochem.*, **2021**, *169*, 207–213.

[20] Kawai, F.; Oda, M.; Tamashiro, T.; Waku, T.; Tanaka, N.; Yamamoto, M.; Mizushima, H.; Miyakawa, T.; Tanokura, M. A Novel Ca2+-Activated, Thermostabilized Polyesterase Capable of Hydrolyzing Polyethylene Terephthalate from Saccharomonospora Viridis AHK190. *Appl. Microbiol. Biotechnol.*, **2014**, *98*, 10053–10064.

[21] Kawabata, T.; Oda, M.; Kawai, F. Mutational Analysis of Cutinase-like Enzyme, Cut190, Based on the 3D Docking Structure with Model Compounds of Polyethylene Terephthalate. *J. Biosci. Bioeng.*, **2017**, *124*, 28–35.

[22] Oda, M.; Yamagami, Y.; Inaba, S.; Oida, T.; Yamamoto, M.; Kitajima, S.; Kawai, F. Enzymatic Hydrolysis of PET: Functional Roles of Three Ca2+ Ions Bound to a Cutinase-like Enzyme, Cut190*, and Its Engineering for Improved Activity. *Appl. Microbiol. Biotechnol.*, **2018**, *102*, 10067–10077.

[23] Perz, V.; Zumstein, M.T.; Sander, M.; Zitzenbacher, S.; Ribitsch, D.; Guebitz, G.M. Biomimetic Approach to Enhance Enzymatic Hydrolysis of the Synthetic Polyester Poly(1,4-Butylene Adipate): Fusing Binding Modules to Esterases. *Biomacromolecules*, **2015**, *16*, 3889–3896.

[24] Herrero Acero, E.; Ribitsch, D.; Dellacher, A.; Zitzenbacher, S.; Marold, A.; Steinkellner, G.; Gruber, K.; Schwab, H.; Guebitz, G.M. Surface Engineering of a Cutinase from Thermobifida Cellulosilytica for Improved Polyester Hydrolysis. *Biotechnol. Bioeng.*, **2013**, *110*, 2581–2590.

[25] Thumarat, U.; Kawabata, T.; Nakajima, M.; Nakajima, H.; Sugiyama, A.; Yazaki, K.; Tada, T.; Waku, T.; Tanaka, N.; Kawai, F. Comparison of Genetic Structures and Biochemical Properties of Tandem Cutinase-Type Polyesterases from Thermobifida Alba AHK119. *J. Biosci. Bioeng.*, **2015**, *120*, 491–497.

[26] Martinelle, M.; Holmquist, M.; Clausen, I.G.; Patkar, S.; Svendsen, A.; Hult, K. The Role of Glu87 and Trp89 in the Lid of Humicola Lanuginosa Lipase. *Protein Eng. Des. Sel.*, **1996**, *9*, 519–524.

[27] Skjold-Jørgensen, J.; Vind, J.; Moroz, O. V; Blagova, E.; Bhatia, V.K.; Svendsen, A.; Wilson, K.S.; Bjerrum, M.J. Controlled Lid-Opening in Thermomyces Lanuginosus Lipase- An Engineered Switch for Studying Lipase Function. *Biochim. Biophys. acta. Proteins proteomics*, **2017**, *1865*, 20–27.

[28] Billig, S.; Oeser, T.; Birkemeyer, C.; Zimmermann, W. Hydrolysis of Cyclic Poly(Ethylene Terephthalate) Trimers by a Carboxylesterase from Thermobifida Fusca KW3. *Appl. Microbiol. Biotechnol.*, **2010**, *87*, 1753–1764.
